# Recent Advances in the Lithium Recovery from Water Resources: From Passive to Electrochemical Methods

**DOI:** 10.1002/advs.202201380

**Published:** 2022-07-27

**Authors:** Luisa Baudino, Cleis Santos, Candido F. Pirri, Fabio La Mantia, Andrea Lamberti

**Affiliations:** ^1^ DISAT Dipartimento di Scienza Applicata e Tecnologia Politecnico di Torino corso Duca degli Abruzzi 24 Torino 10129 Italy; ^2^ Istituto Italiano di Tecnologia Center for Sustainable Future Technologies Via Livorno 60 Torino 10144 Italy; ^3^ Energiespeicher‐ und Energiewandlersysteme Universität Bremen Bibliothekstraße 1 28359 Bremen Germany

**Keywords:** brine/seawater, electrochemical processes, ion sieve, lithium recovery, membrane

## Abstract

The ever‐increasing amount of batteries used in today's society has led to an increase in the demand of lithium in the last few decades. While mining resources of this element have been steadily exploited and are rapidly depleting, water resources constitute an interesting reservoir just out of reach of current technologies. Several techniques are being explored and novel materials engineered. While evaporation is very time‐consuming and has large footprints, ion sieves and supramolecular systems can be suitably tailored and even integrated into membrane and electrochemical techniques. This review gives a comprehensive overview of the available solutions to recover lithium from water resources both by passive and electrically enhanced techniques. Accordingly, this work aims to provide in a single document a rational comparison of outstanding strategies to remove lithium from aqueous sources. To this end, practical figures of merit of both main groups of techniques are provided. An absence of a common experimental protocol and the resulting variability of data and experimental methods are identified. The need for a shared methodology and a common agreement to report performance metrics are underlined.

## Graphical abstract

The demand of lithium in the last decade has grown exponentially, mainly driven by the electromobility and stationary energy storage sectors. In this review, a comprehensive overview of the available solutions to recover lithium from water resources both by passive and electrochemical techniques is given. Accordingly, practical figures of merit of both groups of techniques are identified and suggested.

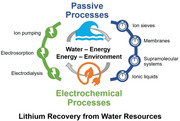

## 1 Introduction

**Figure 1:**
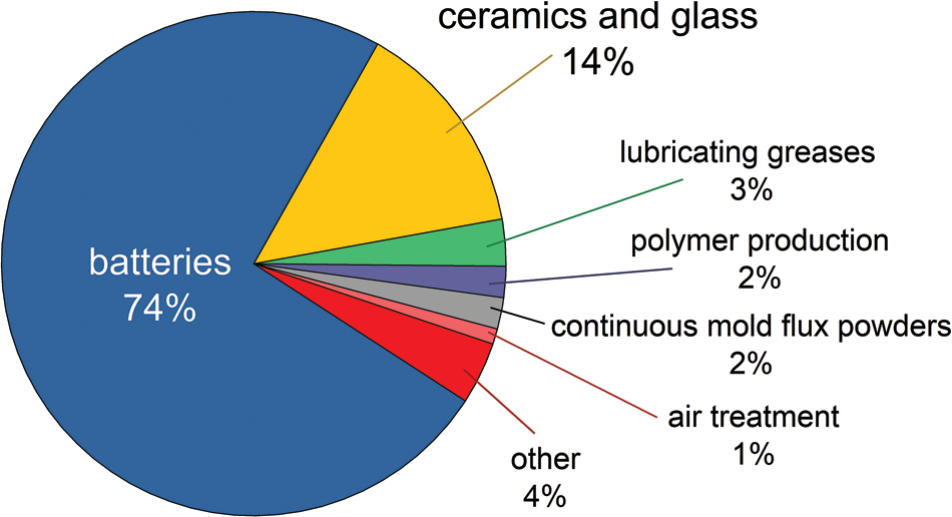
Global end‐use market of Lithium, according to.^[^ ^10^ ^]^

Lithium has been playing a vital role in the energy production economy in the past decades. Twenty‐fifth element on earth for abundancy, lithium is widely known for its low density (0.534 g cm^−3^), its low electrode potential in the electrochemical scale (−3.045 V)^[^ ^1^, ^2^ ^]^ and its high specific heat capacity.^[^ ^3^ ^]^ The combination of such interesting characteristics makes lithium the ideal element to produce both primary (single‐discharge) and secondary (rechargeable) batteries that are now extensively used in several applications. In particular, lithium‐ion batteries are invaluable candidates for both portable electronic devices and precision electronics thanks to their high specific energy density (100–265 Wh kg^−1^), specific power (250–340 W kg^−1^), and a lifetime of 400–1200 cycles.^[^ ^2^, ^4^, ^5^, ^6^ ^]^ One can also find them in electric and hybrid vehicles^[^ ^7^ ^]^ and even in smart grids for energy storage.^[^ ^8^ ^]^ The lithium‐ion batteries (LIBs) production represents nowadays around 37% of the world market of batteries and the previsions are that this share will only increase in the upcoming years.^[^ ^3^ ^]^ However, the batteries market, although having risen in the past few years from 35% to 74% of the lithium global end‐use market, and being the first sector one generally thinks of when talking about lithium, is not the only field of application in constant need of lithium.^[^ ^3^, ^9^, ^10^ ^]^ Before the advent of portable batteries, lithium was already employed in several fields (**Figure** **1**) that are still using it. Among those, the most important one is the production of ceramics and glass materials, where lithium is used to lower the melting point and the viscosity of the process, but also to increase the hardness of glasses and reduce their thermal expansion when added in the form of lithium oxide. Its application in the aluminum electrolytic refinery process produces aluminum alloys suitable for aerospace applications, and most lubricants and greases used in high‐performance sectors contain some percent of lithium. Further uses less known are in air cooling systems in the form of salts to adsorb humidity, as additives in polymer production, and in the cure of bipolar disorders. Finally, one of the lithium isotopes is also used as fuel for nuclear energy reactors when transformed into tritium.^[^ ^1^, ^2^, ^3^, ^9^, ^10^, ^11^, ^12^, ^13^, ^14^, ^15^ ^]^

As the importance of lithium in today's economy is ever‐growing, its price is also evolving, doubling in the last two years. The United States Geological Survey (USGS) Report of Mineral Commodities 2022 indeed reports that the annual average price in dollars per metric ton^2^ for battery‐grade lithium carbonate (Li_2_CO_3_) went from 8000 in 2020 to 17000 in 2021.^[^ ^10^ ^]^ The high cost of production for such a material mainly depends on the complexity of the processes involved in its extraction. Lithium is not found in nature in its elemental form due to its high reactivity. Therefore, successive steps of clarification and filtration to remove solid contaminants, and concentration and purification to obtain the final product are necessary. The energy and time‐consuming processes, the treatment of wastewater, and the use of strong chemical agents are also factors that need to be taken into consideration when talking about the cost of the end product.^[^ ^5^, ^12^, ^16^ ^]^ It is therefore necessary to conduct an accurate analysis of lithium availability, the state of its resources, and its production techniques. Furthermore, life cycle assessment analyses can be of great help when determining the sustainability and suitability of a process.^[^ ^17^, ^18^, ^19^ ^]^

### 1.1 Lithium Availability: Resources & Reserves

When talking about a mineral availability, it is important to note that the values reported in literature may greatly differ depending on the terminology and the methodology used to account for the deposits. A first distinction must be made between the terms resource and reserve. As previously explained and stated in the USGS Reports, a resource is the geologically amount of an element in any form available for exploitation, whereas a reserve is the part of a resource that possesses the quality criteria to be recovered from a socioeconomic and technological point of view.^[^ ^6^, ^10^, ^12^, ^14^, ^16^, ^20^ ^]^ Based on these definitions, while the resource figure becomes more appealing in the academic world, the reserve one is more functional in the industrial world, depending on the technologies available and the quality criteria of production.

Lithium resources can be divided into primary and secondary resources: the primary ones comprehend minerals, seawater, and brines, that is where lithium can be naturally found.^[^ ^1^, ^3^ ^]^ Conversely, all artificial deposits of lithium are comprised in secondary resources.

From a historical point of view, the first lithium resources are pegmatite districts and related magmatic deposits from which Li_2_CO_3_ can be extracted. Those are geologically widespread, differently from the brines, but rare‐metals pegmatites are uncommon and often have already exploited in the past for the extraction of tin and tantalum.^[^ ^4^, ^7^, ^12^, ^15^ ^]^ Although lithium is present in over 145 minerals, it can be extracted only from five of them: spodumene, lepidolite, petalite, amblygonite, and eucryptite.^[^ ^21^ ^]^ This is mostly due to the combination of their hardness, the difficult accessibility of the belt‐like deposits and their limited content of lithium.^[^ ^20^ ^]^ Nonetheless, spodumene mining in Australia accounts for 85% of pegmatite extractions and petalite is commonly used in glass production thanks to its high iron content.^[^ ^5^, ^21^ ^]^ Lepidolite, on the other hand, has lost appeal for lithium extraction due to its high fluorine content.^[^ ^4^, ^20^ ^]^ As far as sedimentary rocks are concerned, hectorite and jadarite are the lithium‐rich clay‐like minerals worth mentioning.^[^ ^5^, ^20^ ^]^

Secondary resources, as was previously mentioned, are artificial deposits of lithium. Among those can be found spent LIBs, oilfield wastewaters from oil platforms, as they usually contain high concentration of metals and rare earth elements after being extracted from deep wells during oil drilling, and even microorganisms that are able to bioaccumulate lithium.^[^ ^13^, ^16^, ^21^, ^22^, ^23^ ^]^ As the market share of LIBs and in particular of secondary batteries is growing by the day, the importance of managing and discarding wastes equally rises. Although the recycling processes of spent LIBs generally recover high valuable metals like nickel and cobalt, whereas lithium is left in the final slag, it is expected that 90% of lithium could be recovered from this kind of secondary resources.^[^ ^2^, ^16^, ^24^ ^]^ Furthermore, the governments' directives to increase the recycling rate to prevent environmental pollution make the extraction of lithium from spent LIBs a topic worth mentioning.^[^ ^23^ ^]^ The usual processes to recycle batteries count pyrometallurgical, hydrometallurgical, and cryogenical methods. Whereas pyrometallurgical methods are generally used for large‐scale metal recovery and are thus unsuitable for lithium, hydrometallurgical methods, consisting of leaching, metal separation from the solution and metal recovery at the solid‐state are generally employed.^[^ ^23^ ^]^ The environmental impact and energy consumption of a hydrometallurgical process recovering lithium as a salt, an intermediate physical recycling process recovering Li_2_CO_3_, and a direct recycling process yielding LiMn_2_O_4_ were analyzed by Dunn et al. highlighting how the latter can significantly reduce the energy consumption.^[^ ^24^, ^25^ ^]^ Finally, electrochemical methods were recently proposed to recover lithium from spent LIBs due to the small electrolyte volumes involved.^[^ ^25^, ^26^ ^]^

**Figure 2:**
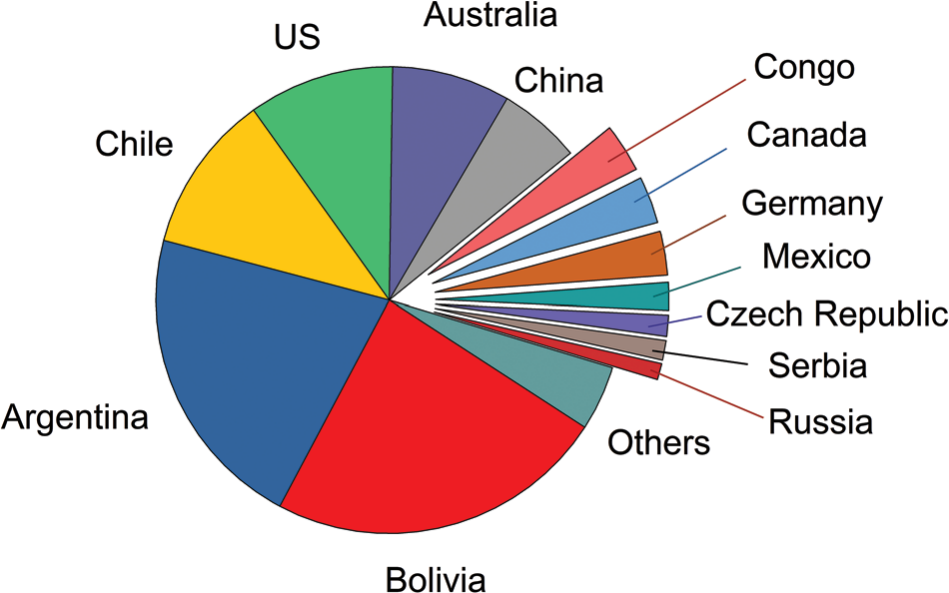
Division of world lithium resources worldwide according to the USGS 2022.^[^ ^10^ ^]^

The amount of lithium resources reported in literature is highly variable and depends on the methodology used, since it is conditioned by the amount of deposits considered. Gruber et al. reported around 38.7 Megatons (Mt) in 2011, Kesler et al. 30.9 Mt in 2012, Grosjean et al. between 37.1 and 43.6 Mt in 2012, Vikström et al. 65 Mt in 2013, Flexer et al. 54.1 Mt in 2018. ^[^ ^4^, ^6^, ^12^, ^16^, ^20^ ^]^ The latest data from the USGS 2022 account for 89 Mt of lithium worldwide, spread among different countries as shown in **Table** **1** and **Figure** **2**.^[^ ^10^ ^]^

**Table 1:** World lithium resources according to the USGS 2022.^[^ ^10^ ^]^ Only the countries with more than 1 Million tons of lithium are reported

Country	Resources [Mt]	Mining reserves [Mt]
Bolivia	21.0	–
Argentina	19.0	2.20
Chile	9.8	9.20
US	9.0	0.75
Australia	7.3	5.70
China	5.1	1.50
Congo	3.0	–
Canada	2.9	0.37
Germany	2.7	–
Mexico	1.7	–
Czech Republic	1.3	–
Serbia	1.2	–
Russia	1.0	–
Others	4.0	3.07

When looking at the worldwide deposits, the first thing that is to be noticed is the uneven distribution of these resources. Gruber et al. report that around 83% of the world's resources are contained in the top ten lithium resources, of which six are brines, two pegmatite, and two sedimentary rocks deposits, but of them only three were producing lithium in 2011: the brines Atacama (in Bolivia), Qaidam and Zabuye (in China).^[^ ^16^ ^]^ This disparity of availability of an important element such as lithium, which is expected to play a vital role in the new green technologies, highlights the need for sustainable governance of such geochemically scarce metal.^[^ ^27^ ^]^

Although studies may differ on the estimated amount of resources, they all agree on the fact that water resources account for 2/3 of lithium available. Both Gruber et al. and Swain report that about 65–66% of lithium is present in brines, whereas only 25–6% is in minerals (pegmatites) and 8% in sedimentary rocks.^[^ ^3^, ^16^ ^]^

### 1.2 Availability & Composition of Water Resources

Lithium extraction from aqueous resources has become increasingly attractive in the past years due to the increasing demand of the energy industry. The intensive mining of high‐grade ores is rapidly forcing the mining industry to move to lower grade ones. Although these ores may have a theoretically high amount of lithium, they also entail higher costs for energy and water consumption thus lowering their economic interest.^[^ ^28^, ^29^ ^]^ Conversely, lithium harvesting from aqueous resources presents many advantages. Focusing first on seawater, although lithium concentration is very low, with an average of 0.17 ppm, it is the most abundant metal ion after the four already commercially extracted (sodium, magnesium, calcium, and potassium). Its total amount is estimated to be of 231 Gigatons and both Bardi and more recently Can Sener et al. deemed its recovery from seawater economically feasible.^[^ ^28^, ^30^ ^]^ Moreover, although seawater composition varies due to tides, temperature, and geographical factors, its composition is fairly homogeneous and would not result in a degradation of the purity grade of the mineral harvested. Although high amounts of energy would be required, a significant decrease of cost can be obtained by coupling this kind of extraction with desalination plants and using the retentate of such industrial plants.^[^ ^29^ ^]^

Lithium extraction from brines, on the other hand, has been exploited successfully for the past years ever since the first extraction as by‐product of sodium carbonate in 1938 at Searles Lake (USA).^[^ ^15^ ^]^ Brines are water solutions with a salinity higher than seawater, typically more than 50 g L^−1^, and can be divided into geothermal brines, oilfield brines and continental brines. It is estimated that close to two third of lithium commercially extracted today comes from continental brines.^[^ ^5^, ^6^ ^]^ This kind of extraction is acknowledged to be more advantageous than from ores, not only because of a greener and less intensive approach, but also because of its cost‐effectiveness. According to Grosjean et al. the lithium production cost from brines was estimated to be less than half of that from spodumene ores (around 2–3 US$ kg^−1^ vs 6–8 US$ kg^−1^).^[^ ^4^ ^]^ The brine resources are mostly concentrated in the Puna Plateau in South America, a high altitude Andean region also called Lithium Triangle. It is estimated that the salars of Bolivia, Chile, and Argentina of the Puna Plateau account for circa 80% of the world lithium brine resources. China bears the second largest concentration of high salinity lake, in the Qinghai‐Tibet Plateau. Both of these regions share the characteristics of being in high altitude areas, having climates that allow high evaporation rates, i.e., arid climates, and being associated with geothermal and igneous activity.^[^ ^6^, ^31^ ^]^ However, even though there may be similarities between different salars, the estimation of those resources is a very complex work and intrinsically needs a multidisciplinary approach. The main factors to take into account when attempting to assess the situation of a salar are seasonal fluctuations, geological and hydrological characteristics such as interactions between solid salts and brines, or with non‐evaporite minerals, and the inherent composition inhomogeneity due to the genesis of these deposits.^[^ ^31^ ^]^

The extraction of lithium from both mineral ores and aqueous resources requires several preprocessing steps. Whereas mineral ores are based on mature technologies and are currently well exploited at an industrial stage, the latter still require the development of novel processes, which would allow untapping their larger portion. Therefore, the aim of this paper is reviewing lithium harvesting from water‐based resources.

After a brief excursus on lithium worldwide availability and the different types of deposits one can encounter, we focus our attention on seawater and other water resources. To have a complete frame of reference, we compiled a table of the composition of water basins containing lithium (**Table** **2**). We considered the most common Li‐rich brines and closed water basins. The Mediterranean Sea and the Arabian Gulf were also included in this list, although not properly closed basins, as their composition has been previously analyzed for desalination purposes. We will now proceed to analyze the different processes available nowadays to harvest lithium. This research field has caught a lot of attention in the last years and although several different techniques have been studied and developed a common protocol is still missing. The aim of our work is therefore to give a comprehensive overlook at the recovering methods available and to suggest some guidelines to make the comparisons of different techniques and materials possible. In order to do so, we propose some figures of merits to compare the different techniques.

**Table 2:** Composition of different water resources. Ion compositions are given in wt%

Water resource	Country	Li [wt%]	Mg [wt%]	Na [wt%]	K [wt%]	B [wt%]	Ca [wt%]	Cl [wt%]	SO_4_ [wt%]	Mg:Li	Refs.
Salar de Atacama	Chile	0.157	0.965	9.10	2.36	0.04	0.045	18.95	1.59	6.14	^[^ ^32^ ^]^
Hombre Muerto	Argentina	0.068–0.121	0.018–0.140	9.90–10.30	0.24–0.97	N.A.	0.019–0.090	15.80–16.80	0.53–1.14	0.26–1.15	^[^ ^32^ ^]^
Salar de Olaroz	Argentina	0.033	0.323	9.46	0.66	0.04	0.059	14.06	1.01	9.79	^[^ ^25^ ^]^
Zabuye	China	0.049	0.003	7.29	1.66	N.A.	0.011	9.53	N.A.	0.05	^[^ ^32^ ^]^
Salar de Uyuni	Bolivia	0.032	0.650	7.06	1.17	0.07	0.031	5.00	N.A.	20.31	^[^ ^32^ ^]^
Taijinar	China	0.031	2.020	5.63	0.44	N.A.	0.020	13.42	3.41	65.16	^[^ ^32^ ^]^
Chaerhan Lake	China	0.001–0.120	0.70–11	N.A.	N.A.	N.A.	N.A.	N.A.	N.A.	87–1837	^[^ ^33^ ^]^
Clayton Valley	USA	0.016	0.019	4.69	0.40	0.005	0.045	7.26	0.34	1.16	^[^ ^32^ ^]^
Salton Sea	USA	0.010–0.040	0.070–0.570	5.00–7.00	1.30–2.40	0.04	2.260–3.900	14.20–20.90	42–50	7.00–14.25	^[^ ^32^ ^]^
Searles Lake	USA	0.005	N.A.	11.08	2.53	N.A.	0.002	12.30	4.61	N.A.	^[^ ^32^ ^]^
Great Salt Lake	USA	0.002	0.500–0.970	3.70–8.70	0.26–0.72	0.007	0.026–0.036	7.00–15.60	0.94–2.00	278–539	^[^ ^32^ ^]^
Mediterranean Sea	Algeria & Gulf of Cádiz	1.7E‐5	0.130	1.80	0.004	4E‐4	0.004	2.20	0.27	7.6E+3	^[^ ^34^, ^35^ ^]^
Geothermal	Western Carpathian Region	0.067	0.220	44.00	0.43	0.66	0.270	49.00	N.A.	3.28	^[^ ^36^, ^37^ ^]^
Dead Sea	Israel	0.001	3.090	3.01	0.56	3E‐4	1.290	16.10	0.06	2.5E+3	^[^ ^32^ ^]^
Red Sea	Saudi Arabia	N.A.	0.070	1.43	0.02	N.A.	0.020	2.22	0.31	N.A.	^[^ ^38^ ^]^
Arabian Gulf	Kuwait	N.A.	0.180	1.58	0.05	N.A.	0.050	2.30	0.32	N.A.	^[^ ^38^ ^]^
Typical seawater		1.8E‐5	0.130	1.05	0.04	4E‐4	0.040	1.90	0.26	7.2E+3	^[^ ^38^ ^]^

**Figure 3:**
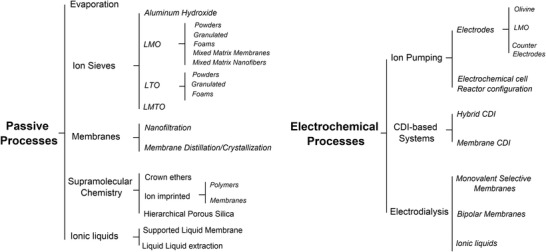
Schematic overview of the processes analyzed in this study.

In this study we divide the processes into two main groups, called passive adsorption and electrochemical adsorption, following the nomenclature previously proposed by LiVecchi for seawater mining.^[^ ^39^ ^]^ As their names suggest, the first group is characterized by the absence of an external electric voltage, which is instead used in electrochemical processes. These two categories are further divided based on the specific technique or driving mechanism employed, as can be seen in **Figure** **3**, which can act as a roadmap of this review. Among passive processes the main techniques here analyzed are evaporation, and adsorption using ion sieves, membrane, supramolecular chemistry, and liquid–liquid extraction with ionic liquids. Ion pumping, ion electrosorption, and electrodialysis techniques will be instead reviewed among the electrochemical processes. This review therefore has the unique feature of containing not only an overview of the different techniques and materials used in lithium recovery, but also a form of critical comparison among different experimental procedures (figures of merits) and a section with recommendations for good experimental practice.

## 2 Processes for Li Extraction: Passive Processes

### 2.1 Evaporation

The evaporation technology is the only one to the best of our knowledge that currently allows an industrial exploitation from brines. According to Flexer et al. in 2018 there were worldwide eight active facilities, of which two were in Argentina, exploiting Salar de Olaroz and Salar de Hombre Muerto, two in Chile, at the Salar de Atacama, three in China that at that time were still at a piloting stage, at Chaerhan Salt Lake, West Taijinar and Zabuye, and lastly one in the USA, at Clayton Valley.^[^ ^6^ ^]^ The small number of evaporation plants compared to the high amount of theoretical exploitable sites can be easily understood when looking at the sequence of steps that this process comprises.

**Figure 4:**
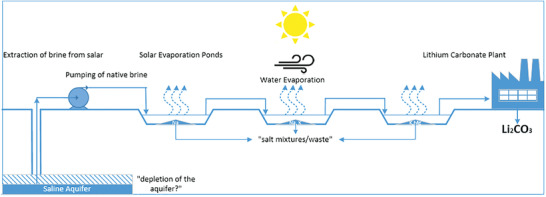
Schematic of the evaporitic technique. Adapted with permission.^[^ ^6^ ^]^ Copyright 2018, Elsevier.

The evaporation process is generally composed by a first stage in which the brine is concentrated by solar and wind evaporation in large ponds. This takes place until a concentration of around 6000 mg_Li_ L^−1^ is achieved, after which the brine is generally pumped into a recovery plant (see **Figure** **4**).^[^ ^6^ ^]^ The successive steps can differ depending on the type of brine processed, i.e., the anion most present in the brine (sulfate, chloride, or carbonate), and the specific plant. After the preconcentration step, the other ions that did not spontaneously precipitate are to be removed by chemical treatments: calcium and boron can be removed by adding CaCl_2_; residual borates can be eliminated by solvent extraction; magnesium ions with the addition of lime. Then the “clean brine” can be treated with Na_2_CO_3_ to salt out Li_2_CO_3_. Once Li_2_CO_3_ is obtained, it can be re‐dissolved and re‐precipitated in order to obtain higher grades of purity.^[^ ^6^, ^11^ ^]^ A detailed description of the industrial steps of the process of precipitation of high purity grades of Li_2_CO_3_ from brine with 6 wt% of lithium after the removal of magnesium can be found in patents from Boryta and co‐workers.^[^ ^40^, ^41^, ^42^ ^]^

Although the evaporation technology proves to be very cost effective by exploiting the climatic conditions, it also presents many shortcomings, and it is not as straightforward as it could seem. Several conditions must be met with for a successful recovery of lithium, the climatic and geological ones being the most important. Consistently arid and windy atmospheres are fundamental since the process can take up to 24 months. The combined use of solar and wind evaporation is one of the main factors that can limit the cost of this kind of operation. The geological requisites are instead the presence of a closed basin containing the brine, an associated geothermal activity, and the presence of one or more aquifers.^[^ ^6^, ^15^, ^31^ ^]^ However, the occurrence of all these conditions is not met in several basins outside of the Puna Plateau and China, resulting in high lithium basins in the US becoming unviable.^[^ ^43^ ^]^ Furthermore, the evaporation technique presents a large footprint,^[^ ^6^, ^44^ ^]^ with half a million liters of water evaporated to recover a ton of lithium carbonate, thus requiring an upgrade of the setup normally used in order not to deplete the aquifers used. The application of this technique on oilfield wastewater to recover both lithium, beryllium, and strontium was also studied by adding plants with a high transpiration rate in the wetland.^[^ ^45^ ^]^

Lastly, due to the broad differences in the composition of the basins (see Table 2), the development of a single industrial process capable of exploiting the water‐based lithium resources is challenging. Accurate examples of this are the Uyuni brine, in Bolivia, and the Chaerhan Salt Lake, in China.^[^ ^32^, ^33^ ^]^ The former, although hosting one of the biggest deposits of lithium in the world, also presents a very high magnesium content. In this case, as the pilot scale reported by Flexer in 2018 demonstrated, a high grade final product is difficult to obtain.^[^ ^6^ ^]^ On the contrary, in the case of Chaerhan Salt Lake, the brine can be treated in a plant that also recovers KCl and the treatment only needs implementation to efficiently recover lithium. The combination of a low lithium content and Mg:Li ratio of over 20 is very detrimental to the recovery of high‐quality lithium because of the precipitation of mixed compounds like lithium carnallite, a hydrated mixed chloride of lithium and magnesium, before lithium precipitation. For this purpose, An et al. designed a hydrometallurgical process comprising two stages of precipitation to remove said element.^[^ ^32^ ^]^ In the first stage the removal of magnesium and boron in the form of Mg(OH)_2_, gypsum, and boron occurs by adding lime. In the second stage, sodium oxalate is used to remove residual calcium and magnesium. The process then culminated with solar evaporation and the precipitation of lithium by carbonation at 80–90 °C with the addition of Na_2_CO_3_.

### 2.2 Ion Sieves

Among other passive methods, adsorption might be the most promising in terms of efficiency, quality of the product, and cleanliness of the process. This can also be used in streams that are less concentrated than brines. The most common adsorbents are lithium ion sieves (LIS), first prepared by Volkhin in 1971.^[^ ^46^ ^]^ LIS are inorganic compounds in which template ions are introduced by redox or ion‐exchange reactions and afterward eluted from the structure. The vacancies thus resulting will be ion‐specific for the target ions due to ion screening phenomena and memory effect.^[^ ^47^, ^48^ ^]^ The main families of LIS are aluminum hydroxide ion sieves, lithium manganese oxide ion sieves (LMO), and lithium titanium oxide ion sieves (LTO), as lithium can be adsorbed into or can easily penetrate nonstoichiometric crystalline networks of Mn and Ti oxides or Al hydroxide. An intermediate version of mixed lithium manganese and titanium oxide (LMTO) is also possible.

The superior selectivity of LIS results in high purity lithium products and high lithium separation efficiency. This also entails the fact that no further purification is required, and that from a theoretical point of view a high uptake capacity is paired with small losses of material during regeneration. Additionally, LIS is said to be more environmentally friendly and less energy consuming than other technologies such as solvent extraction or precipitation methods.^[^ ^47^, ^48^ ^]^ However, LIS technology is not exempt of drawbacks, both specific to the general process and the used stoichiometry. The first and most obvious drawback of ion sieves in general is that while less energy consuming, LIS technologies often require longer spans of time to operate as they generally need to reach a thermodynamic equilibrium.

The preparation method of the precursors heavily influences the degree of crystallinity and the regeneration ability of ion sieves. The processes used to synthesize them are very similar from one type of LIS to another and can be divided into two main classes: solid‐state methods and soft chemical methods. The first one consists in calcination and microwave combustion, i.e., methods in which the reactants are in a solid state. Instead, soft chemical methods include those in which the compounds are dissolved in aqueous solutions: hydrothermal method, sol‐gel, molten‐salt synthesis, coprecipitation. The advantages and disadvantages of each method and their use for each LIS are listed in **Table** **3**.

**Table 3:** Comparison between the different preparation methods of each LIS type and their forming process^[^ ^47^, ^48^ ^]^

		Advantages	Disadvantages	Refs.	Best performance reported
Preparation method	Calcination	Simple operation	Uneven properties, time and energy consuming	LMO^[^ ^50^, ^51^, ^52^, ^53^, ^54^, ^55^, ^56^ ^]^ LTO^[^ ^57^, ^58^, ^59^, ^60^, ^61^, ^62^, ^63^, ^64^, ^65^, ^66^, ^67^, ^68^, ^69^ ^]^ LMTO^[^ ^70^ ^]^	LMO: 11.4 mg_Li_ g^−1^ after 24h in 25.78 mg_Li_ L^−1[^ ^55^ ^]^ LTO: 24.57 mg_Li_ g^−1^ after 2 h in 25.78 mg_Li_ L^−1[^ ^61^ ^]^
	Microwave combustion	More homogeneous properties, less time consuming	Setup more complicated		
	Hydrothermal	Simple operation, cost effective, evenly mixing of raw materials, different morphologies possible	Non uniform temperatures, time consuming	LMO^[^ ^50^, ^71^, ^72^, ^73^, ^74^, ^75^ ^]^ LTO^[^ ^64^, ^76^, ^77^, ^78^, ^79^ ^]^	LMO: 40 mg_Li_ g^−1^ after 3 d in seawater^[^ ^71^ ^]^ LTO: 39.43 mg_Li_ g^−1^ after 120h in 120 mg_Li_ L^−1 [^ ^76^ ^]^
	Sol‐gel	Good homogeneity, less time consuming, low calcination temperature	Mostly suitable for nanoparticles	LMO^[^ ^74^ ^]^ LTO^[^ ^62^, ^80^ ^]^	LMO: 10 mg_Li_ g^−1^ after 48 h in 6 mg_Li_ L^−1 [^ ^74^ ^]^ LTO: 39.20 mg_Li_ g^−1^ after 24 h in 2 g_Li_ L^−1[^ ^62^ ^]^
	Molten‐salt synthesis	High purity, good for high concentrations, simple operation	Safety risks for potential metallic fluorides, corrosive acidic salts, heavy metal salts		
	Co‐precipitation	Simple operation, cost effective, can be used for doped LMO‐type LIS	Uneven size distribution, local high ion concentration		
Forming process	Powders	Superior adsorption capacity	Difficult to handle	LMO^[^ ^50^, ^51^, ^71^, ^72^, ^73^, ^75^, ^81^ ^]^ LTO^[^ ^57^, ^58^, ^59^, ^60^, ^62^, ^63^, ^65^, ^67^, ^76^, ^77^, ^78^, ^82^, ^83^, ^84^, ^85^ ^]^	LMO: 40 mg_Li_ g^−1^ after 3 d in seawater^[^ ^71^ ^]^ LTO: 13 mg_Li_ g^−1^ after 24h in 10 mg_Li_ L^−1[^ ^57^ ^]^
	Granulation	Good mechanical strength, can work in industrial column operations	Lower adsorption capacity due to a binding element	LMO^[^ ^51^, ^55^, ^74^, ^86^, ^87^ ^]^ LTO^[^ ^66^, ^68^, ^69^ ^]^	LMO: 10 mg_Li_ g^−1^ after 48h in 6 mg_Li_ L^−1[^ ^74^ ^]^ LTO: 25 mg_Li_ g^−1^ after 3 h in 200 mg_Li_ L^−1[^ ^66^ ^]^
	Foaming	Superior mechanical properties, good regeneration performance, more adaptable in terms of shape and recovery processes	Lower adsorption capacity due to a foaming element, looser internal skeleton can degrade more rapidly, hazardous substances, expensive	LMO^[^ ^54^, ^75^, ^88^ ^]^ LTO^[^ ^64^, ^80^, ^89^ ^]^	LMO: 7.77 mg_Li_ g^−1^ after 24 h in 7 mg_Li_ L^−1[^ ^88^ ^]^ LTO: 14 mg_Li_ g^−1^ after 24h in 7 mg_Li_ L^−1[^ ^89^ ^]^
	Membrane	Easily scalable and modular	Expensive, complex fabrication	LMO^[^ ^90^, ^91^, ^92^, ^93^ ^]^	9.5 mg_Li_ g^−1^ after 15 d in seawater^[^ ^92^ ^]^
	Nanofibers	Good tunability of properties	May require further forming	LMO ^[^ ^94^, ^95^, ^96^ ^]^ LTO ^[^ ^61^ ^]^	LMO: 12 mg_Li_ g^−1^ after 24 h in 7 mg_Li_ L^−1[^ ^95^ ^]^ LTO: 24.57 mg_Li_ g^−1^ after 2h in 25.78 mg_Li_ L^−1[^ ^61^ ^]^

Unfortunately, all these processes provide the LIS in the powder form, which can be difficult to use when processing aqueous solutions. Therefore their use is generally still relegated to the laboratory scale whereas electrochemical methods have been implemented in pilot‐scale facilities.^[^ ^49^ ^]^ Although this kind of morphology is the one most used for laboratory‐scale experiments and often the reference for the other morphologies efficiencies, recovering powders can be an energy‐consuming task and losses of active material are frequent. For this purpose, several forming processes have been employed: granulation, foaming, and membrane preparation are just the main ones. In the case of granulation, the addition of a binder results in the formation of agglomerates, similar to pellets, easier to handle, but this can also result in lower adsorption values due to death volumes. A similar approach is used during foaming, but in this case the structure is highly permeable and presents a wider adaptability in terms of materials, though the products are also more expensive and easier to degrade. On the other hand, membranes are easily scalable and suitable to industrial applications but their production can be of difficult means. Each of these methods has, as always, its own pros and cons and there is no unique material or forming process that can solve all challenges. An overview of these forming processes in relation with the analyzed works can be found in Table 3.

Furthermore, in order to overcome the intrinsic limitations of each kind of LIS, several attempts of creating either hybrid LIS, like mixed manganese‐oxide and titanium‐oxide LIS (LMTO), or doping canonical ones to improve the adsorption capacity were and are still pursued.^[^ ^47^, ^65^ ^]^ An example of this can be seen in the work from Chitrakar et al. in which they synthesized several magnesium‐doped lithium manganese oxides and reported that both the chemical stability and the lithium adsorption capacity increased with the Mg:Mn ratio.^[^ ^97^ ^]^ In particular, when the ratio was equal to 0.33 the lithium capacity of the sorbent reached 23–25 mg_Li_ g^−1^ at pH 6.5 and the dissolution of manganese decreased from 5.8 wt% to 0.25 wt%. The same authors also studied the adsorption properties of iron‐doped LMOs.^[^ ^98^ ^]^ The doping confirmed the trend previously observed of better stability and performance of the adsorbent when increasing the ratio Fe:Mn. Optimal properties were found for a ratio Fe:Mn equal to 0.1 and an uptake of 18.1 mg_Li_ g^−1^ was attained from a brine of composition similar to the one from Salar de Uyuni, reaching even 28 mg_Li_ g^−1^ when buffering the brine with 1 m NaOH. More recently, Dai et al. presented an aluminum‐doped lithium titanium oxide which was able to adsorb 32.12 mg_Li_ g^−1^ from a LiCl solution of 252 mg_Li_ L^−1^, while Zhou et al. reported a granulated zirconium‐doped lithium titanium ion sieve that showed high selectivity in a real case scenario of Qaidam lake brine.^[^ ^65^, ^68^ ^]^ In the following, the main families of LIS will be studied, pointing out relevant case studies and any shortcomings of the technologies proposed. More detailed information concerning ion sieves can be found in Xu et al. and Safari et al. ^[^ ^47^, ^99^ ^]^

#### 2.2.1 Aluminum Hydroxide Ion Sieves

Although less popular than LMO and LTO due to a theoretical smaller uptake capacity, aluminum hydroxide ion sieves have been known for several decades.^[^ ^100^ ^]^ The aluminum hydroxide capacity of adsorbing lithium and its use for precipitating and recovering lithium from brines has been known since the 1960s when Goodenough patented this recovery method.^[^ ^101^ ^]^ However, it was mainly used in the 1980s in the form of suspended hydrous alumina in ion exchange resins, which form crystalline double hydroxides when in contact with lithium ions.^[^ ^102^, ^103^, ^104^, ^105^, ^106^ ^]^

**Figure 5:**
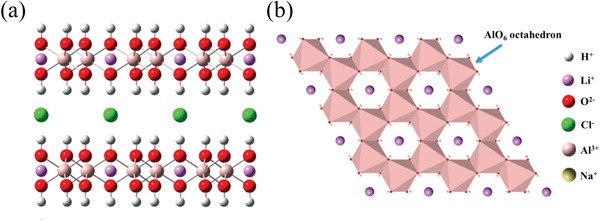
a) Front view and b) top view of LDHs. Reproduced under the terms of the CC‐BY license.^[^ ^118^ ^]^ Copyright 2019, The Authors, published by MDPI.

From a crystallographic point of view, the layered structure of lithium dialuminate has been known as the product of lithium insertion in aluminum hydroxide (bayerite) from the clay industry and has been since used for its catalytic properties.^[^ ^100^ ^]^ Of particular interest is the insertion of lithium salts into the structure of gibbsite (or *γ*‐Al(OH)_3_) that results in compounds with the composition [LiAl_2_(OH)_6_]X*yH_2_O with {X = Cl, Br, NO_3_}. When dehydrated by heating the compounds to around 200 °C, the materials present a highly crystalline structure with highly ordered lamellar phases.^[^ ^107^, ^108^ ^]^ These layered double hydroxides (LDHs, see **Figure** **5**) have since been used in a variety of applications, e.g., catalyst, drug delivery, and ion scavenging. A few examples of LDHs used to selectively extract lithium from complex chloride salts systems can be found in Isupov's study. The sorption capacity of LDHs is reported to be around 8–10 mg_Li_ g^−1^ Al_2_O_3_ in a working range of pH between 3 and 8 (when lower the adsorbent will dissolve, when higher the sorption properties are no longer present).^[^ ^109^ ^]^

LDHs have also been reported to selectively extract LiCl from a geothermal brine in a three‐stage bench‐scale column process. The stripping, however, proved to be the most critical step of the process as an over‐depletion of LiCl caused an irreversible degradation of the sorbent that went back to its gibbsite structure.^[^ ^110^, ^111^ ^]^ To avoid these issues another study reported a low flow of deionized water as eluent.^[^ ^112^ ^]^ More recently, sorbents of lithium aluminate in the form of porous activated alumina infused with lithium salts have been reported together with a method to recover lithium from solutions brine‐like.^[^ ^113^, ^114^ ^]^ A comprehensive overview of the patents and methods to recover lithium from geothermal waters with AlOH sorbents can be found in Stringfellow and Dobson.^[^ ^115^ ^]^

In other cases, aluminum‐based powders were not used as sorbents but as a mean to make lithium precipitate as lithium aluminate reaching a precipitation rate of 78.3%.^[^ ^116^ ^]^ A similar idea was investigated by Xiang and co‐workers first to remove Mg from brines with a high Mg:Li ratio and then to recover lithium directly from the brine through the nucleation and successive crystallization of LDHs.^[^ ^117^, ^118^ ^]^ In the first case AlCl_3_ hexahydrate was added to the brine solution and was mixed with an alkaline solution in a colloid mill in order to start the nucleation process. The slurry was then transferred into a crystallization reactor and afterwards was filtered, washed, and dried to perform the solid‐liquid separation. This way, whereas Mg was retained in the solid phase as hydrotalcite, lithium was left in the liquid solution to undergo further recovery. Hydrotalcite presents a lamellar structure in which Mg^2+^ and Al^3+^ are alternating in the layer and carbonate anions equilibrate the charges in the interlayer and cannot host lithium ions, hence more than 90% of it remained in the liquid phase. The second study started where the first finished. AlCl_3_ hexahydrate was added to the Mg‐depleted solution and then put in the crystallization reactor together with the NaOH buffer. The slurry underwent the same procedure as the previous one. The LiAl‐LDHs were then dispersed in deionized water (DIW) at temperature of 85 °C in order to recover LiCl as an aqueous solution and Al(OH)_3_ as a solid. Ultimately, a lithium recovery of 86.2% from the LiAl‐LDHs could be reached resulting in a filtrate with 141.6 mg_Li_ L^−1^.

#### 2.2.2 LMO‐Type LIS

**Figure 6:**
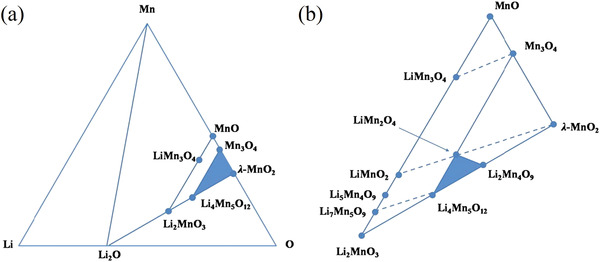
a) Isothermal section of the ternary diagram Li‐Mn‐O at 25 °C and b) inset of the spinel region of interest. Reproduced with permission.^[^ ^47^ ^]^ Copyright 2016, Elsevier.

Lithium manganese oxide ion sieves are, to the best of our knowledge, the most common lithium adsorbents. Not only are they used as proper ion sieves, either in powder or suitably formed for the desired experimental setup, but they are also often used as active additive in membrane processes or in electrodes of active processes. Their versatility mostly depends on the variety of stoichiometric formulas they can present. **Figure** **6** shows an isothermal cross‐section of the ternary phase diagram of Li–Mn–O at 25 °C. The blue area in Figure 6a corresponds to the stoichiometric defect spinel phase, whereas the blue area in Figure 6b represents the active area to prepare precursors of LMO‐type LIS. Discovered by Hunter in 1981, *λ*‐MnO_2_ was the first LMO‐type LIS to be prepared.^[^ ^119^ ^]^ Hunter showed that when treated with an aqueous acid solution, the LiMn_2_O_4_ spinel structure was maintained even though nearly all of the lithium had been removed from its tetrahedral sites. The remaining manganese atoms were still in their original octahedral sites of the ccp oxygen framework and the lattice constant underwent only a small reduction. This structure was then called *λ*‐MnO_2_ in a similar way to the *γ*‐MnO_2_ cathodic reduction. The other most common LMO‐type LIS precursors are Li_1.33_Mn_1.67_O_4_ (or Li_4_Mn_5_O_12_), and Li_1.6_Mn_1.6_O_4_.^[^ ^120^, ^121^ ^]^

The main characteristics that make LIS the most common ones in the lithium recovery field are their superior lithium selectivity, their high lithium adsorption capacities, and an excellent regeneration performance. LTO‐type LIS may present a higher bond energy and are therefore more stable, not undergoing any dissolution along the process. However, LMO‐type LIS has a faster adsorption and are consequently of more interest to potential industrial applications.

Two different mechanisms of lithium adsorption are generally proposed for LMO‐type LIS: ion exchange and redox.^[^ ^47^, ^48^ ^]^ In the first case, lithium is supposedly replaced by protons in the lattice structure. There are no changes in Mn(III) and Mn(IV) sites and the adsorption is pH dependent, as experimentally observed. However, no manganese dissolution is predicted by this model and therefore the spinel structure should maintain its performance along with its structure throughout the operations, which is not in accordance with the observed results. In the case of redox adsorption, on the other hand, the driving force of lithium intercalation and de‐intercalation is the disproportionation of Mn(III) and Mn(IV) under acidic conditions. In this case, Mn(III) is predicted to diffuse to the surface and dissolve in the aqueous solution, whereas Mn(IV) will remain in the crystal structure. This explains the Mn dissolution experimentally observed and the performance decrease, but not the pH dependence. As evidenced by the shortcomings of both models, none of them can properly explain the empirical observations. A combination of the two models has since been proposed in order to explain the experimental evidences.^[^ ^48^ ^]^ It is interesting to note that the lithium extraction from the structure can be performed also electrochemically, as will be discussed in Section 3.1.1.

By conducting pH titration studies on *λ*‐MnO_2_ in both Li‐containing aqueous solutions and solutions of other salts and analyzing the value of pKc, Ooi and co‐workers concluded that three different types of sites exist in LMO:^[^ ^122^, ^123^ ^]^ redox sites, where Mn(III) is oxidized in Mn(IV); Li^+^‐specific insertion sites; nonspecific insertion sites. The authors also concluded that the ratio between the different types of sites depended on the preparation conditions of the spinel. Other studies reflected on the sources of redox and insertion sites and concluded that the presence of Mn(III) in the precursor led to redox sites, whereas the presence of Mn(IV) was responsible for insertion sites.^[^ ^120^ ^]^ Calculations were also performed to model the sites in two different spinel structures: from a theoretical point of view it was concluded that all sites of LiMn_2_O4 were redox sites whereas those in Li_1.33_Mn_1.67_O_4_ were insertion ones. From a more practical point of view a solid solution of the two types of sites was proposed, with the relative quantities depending on the preparation conditions of the samples. In particular, it was found out that at low temperatures, i.e., below 500 °C, insertion sites were mainly formed, whereas redox sites were peculiar of higher temperatures.^[^ ^120^ ^]^

##### LMO‐Type LIS in Powder Form

As previously stated, although less suitable to industrial applications, ion sieves in the powder form are still frequently used in the research field because they are easy to obtain, useful to demonstrate whether a material can act as a sorbent or not and to investigate their properties and characteristics. Chitrakar et al.,^[^ ^71^ ^]^ for example, used the powder form as an experiment bench to evaluate the differences between sorbents prepared in two different ways. The ion sieve synthesized by the hydrothermal method in that study is still to date the most performant LMO in powder form, reaching the equilibrium after 2 d and showing an uptake of circa 35–40 mg_Li_ g^−1^ in seawater. Adsorbents in powder form are also frequently used to conduct fundamental experiments as were the ones linking the adsorption capacity to the pH of the feed solution conducted by Park et al.^[^ ^72^ ^]^ and Wang et al.^[^ ^73^ ^]^

Powdered adsorbents are generally used in a spherical shape but other geometries have also been explored. An example of this can be found in Zhang et al., where nanorods of *β*‐MnO_2_ were prepared via a combination of hydrothermal reaction and low‐temperature calcination of the precursor Li_4_Mn_5_O_12_.^[^ ^50^ ^]^ The samples obtained were mainly monodispersed nanorods of 20–140 nm of diameter and 0.8–4 µm in length. Their adsorption capacity resulted to be 34.7 mg_Li_ g^−1^ after 72 h at 30 °C in a solution of ≈35 mg_Li_ L^−1^.

Due to their superior adsorption capacity, the powder form is also usually employed as a comparison standard when testing ion sieves in other forms to assess their loss of efficiency, as will be seen later.

##### Granulated LMO Adsorbents

Although highly performant, the impracticability of fine powders had already been highlighted in 1990 by Onodera.^[^ ^52^ ^]^ Namely, the nature of this kind of adsorbent makes their handling in successive recovery from sea water difficult. Two possible answers to these problems were proposed in the past years. The first one would be designing suitable containers that present high water permeability but avoid the adsorbent leakage. In this prototype, the inorganic adsorbent was not loaded into the membrane but rather contained into a membrane reservoir.^[^ ^81^ ^]^ However, the most common option is the use of forming technologies to make the powders more manageable and to give them mechanical stability. In the case of granulated adsorbents, the powders are mixed with a binder in order to form agglomerates. Several polymers can be used as binders to create granulates, both biobased and not. The most common biobased binder is the chitin/chitosan couple but cellulose was also investigated. The choice of using them is often motivated by the need to reduce the environmental footprint of the process and their high water permeability to reduce the dead volume.^[^ ^51^, ^55^, ^86^, ^87^, ^124^ ^]^ Although potentially highly performant, this kind of process often requires higher times to reach a thermodynamic equilibrium. On the other hand, binders like PVC can also be used.^[^ ^74^ ^]^

These granulated ion sieves have been widely used in column adsorption setups. A different modus operandi was also proposed in which the active material was loaded as an additive on other structures instead of a binder being added to it. These hybrid beads consisted mostly of silicon spheres modified with ion sieves and could serve different recoveries at the same time. However, the efficiencies still remain quite smaller than the powder adsorbents.^[^ ^52^, ^53^ ^]^

##### Foam‐Type LMO Adsorbents

Although granulation can reach good adsorption performances, the problem of the aqueous solution not being able to reach all the active material is still present. This limitation could be avoided using foams, which present a 3D interpenetrated network. Foams present several advantages compared to granulated adsorbents, namely the possibility of being shaped more freely following the setup requirements and being more easily handled. However, some of the disadvantages of foams are the sometimes hazardous substances that can be released or generated during their production and a higher manufacturing cost with respect to the powders.^[^ ^75^, ^88^ ^]^ A few examples of different binders and techniques can be found in literature for foam adsorbents. While the use of a polyurethane template only allowed small amounts of the active area of the ion sieves to be reached by the solution,^[^ ^75^ ^]^ more environmentally friendly binders such as aqueous agar solutions were investigated and gave more encouraging results.^[^ ^54^, ^125^ ^]^ The use of PVA also allowed to incorporate higher amounts of LMO in the structure, reaching 250 wt% of ion sieves as the best ratio for adsorption purposes. By using cryo‐desiccation and cross‐linking techniques, macroporous flexible foams were realized but their cycling abilities were inversely proportional to the loading amount of ion sieves into them.^[^ ^88^ ^]^ Dried crosslinked alginate composites incorporated with HMO ion sieves were also recently reported by Park et al.^[^ ^56^, ^126^ ^]^ In this case the selectivity of both the Al^3+^ crosslinked alginated and the ion sieve powders were combined to recover lithium from concentrated seawater.

##### LMO Mixed‐Matrix Membranes

Membranes adsorbents present themselves as promising candidates for industrial applications due to their modularity and possibility of being set in parallel in cells by stacking or coiling them. In the case of composite membranes made by a polymeric phase loaded with ion sieves adsorbents as fillers, the membranes are called “mixed matrix membranes.” The properties of this kind of adsorbents, being composites materials, will then depend both on the properties of each phase and on their relative ratio.^[^ ^127^ ^]^

Up until recent years, PVC and PVDF were the most used polymeric phase for membrane adsorbents.^[^ ^90^, ^92^ ^]^ Those were obtained by using the solvent exchange method and ion sieves contents lower than in the case of foams were used to avoid surface defects and poor crosslinking. Graphene oxide (GO) was also recently used as a binder in a GO‐*β*‐CD/MnO_2_ membrane. In this case, the hydrothermally grown nanotubes showed promising enrichment properties for diluted lithium solutions.^[^ ^128^ ^]^ More recently, sustainable and recyclable materials like cellulose and bioinspired adsorbents started to raise to attention in lithium recovery applications.^[^ ^91^, ^129^ ^]^ An example of this can be found in Tang et al. who modified a cellulose film prepared as aerogel in ionic liquid and loaded it with thermally prepared H_1.33_Mn_1.67_O_4_ particles.^[^ ^91^ ^]^ The film thus prepared presented superior mechanical and adsorption properties when compared to other adsorbents and a better versatility due to its design.

##### LMO Mixed‐Matrix Nanofiber Networks

Like the mixed‐matrix membranes, mixed‐matrix nanofiber networks present electrospun nanofibers where the initial dispersion was loaded with inorganic ion sieves. The choice of using nanofibers as support for the inorganic adsorbent originates from the need to minimize the dead volume in the binder, responsible for the performance loss with respect to its unbinded form. Furthermore, the electrospinning technique is a convenient technology that allows for a good tunability of the fibers properties. In this case, typical supports for both LMO and LTO ion sieves are polyacrylonitrile (PAN)^[^ ^93^, ^96^ ^]^ and polysulfone (PSf).^[^ ^61^, ^95^ ^]^

#### 2.2.3 LTO‐Type LIS

Lithium titanium oxide ion sieves are, with the LMO‐type, the most common and widely used selective adsorbents. Titanium dioxide has long been used as electrode material in lithium‐ion batteries and solar cells and is one of the transition‐metal oxides most investigated in the field of material sciences due to its many interesting properties. Among the most notable ones there are its nontoxicity and biocompatibility, its environmental friendliness, and corrosion resistance, whereas as far as ionic and electronics properties are concerned, it is worth mentioning that all of its crystal forms are wide‐gap semiconductors with a bandgap of ≈3 eV.^[^ ^130^, ^131^ ^]^ TiO_2_ presents itself in different polymorphs, namely anatase, brookite, rutile, and TiO_2_‐B, discovered by Marchand et al. in 1980.^[^ ^132^ ^]^ The latter takes its name from the similarities that exist between its covalent framework and the one of Wadsley bronze Na*_x_* TiO_2_ and presents more common features with titanates structures than titania itself.^[^ ^131^, ^133^ ^]^ By being the least dense of the four polymorphs, it is considered to be the most stable of them all.^[^ ^134^ ^]^ However, the concept of stability is strictly size‐dependent due to the similar surface energy of the different phases. For instance, although in the bulk form rutile is thermodynamically the most stable form, when going to the nanoscale other polymorphs become more stable. Both ab initio density‐functional and experimental investigations reported that at the nanoscale, the thermodynamically stable phase is anatase if the size is less than 11 nm, brookite between 11 and 35 nm and rutile over 35 nm.^[^ ^135^, ^136^ ^]^

**Figure 7:**
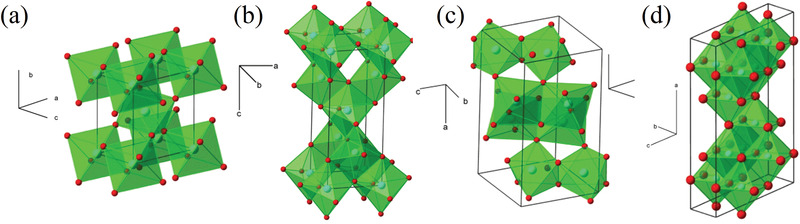
Crystallographic structure of TiO_2_ polymorphs: a) rutile, b) anatase, c) brookite, d) TiO_2_ bronze. Each polymorph has been depicted with its unit cell and crystallographic axes. Titanium atoms are represented in red, Oxygen in light blue, the coordination octahedron is highlighted in green. Adapted with permission.^[^ ^138^ ^]^ Copyright 2009, Elsevier.

Lithium intercalation into the tunnel systems of TiO_2_ frameworks is by now a well‐known phenomenon, as is the fact that the different polymorphs can accommodate the monovalent cation in different stoichiometries. Lithium can be more easily inserted into anatase or TiO_2_‐B than in rutile, thanks to their unit cell being less distorted by the intercalation.^[^ ^137^, ^138^, ^139^ ^]^ Several differences exist when talking about lithium insertion into the different polymorphs of titanium dioxide. First, the different structural unit cell (see **Figure** **7**) must be considered in order to understand the insertion mechanisms. As previously said, anatase presents a tetragonal unit cell as does rutile, brookite an orthorhombic one and TiO_2_‐B has a monoclinic structure characterized by 1D infinite channels. Its open structure allows the TiO_2_‐B to undergo volume changes related to lithium intercalation without distorting its structure. This same distortion resulting by the insertion of a foreign atom into the native structure is the reason for which rutile can accommodate less lithium than the other polymorphs.^[^ ^140^ ^]^

Lithium insertion is a diffusion controlled process depending on the diffusion coefficient and length of TiO_2_ when one considers anatase or rutile phases, but it appears as a pseudocapacitive faradaic process in TiO_2_‐B because of the nanosized network of channels that enhance the local conductivity.^[^ ^138^, ^140^ ^]^ In a similar way to the stability of the various polymorphs, the insertion capacity of lithium into the various structures is both size‐dependent and temperature‐dependent. In his study, Wagemaker demonstrated that lithium solubility in anatase increased when decreasing the size of the hosting particles and that amorphous structures presented higher insertion capacity with respect to the crystalline ones.^[^ ^141^ ^]^ In particular, he showed that a theoretical Li_1_TiO_2_ phase was reachable when the nanoparticles were of 7 nm, greatly increasing lithium insertion with respect to canonical electrochemical insertion into bulk materials that only reach a ratio of Li:Ti of 0.5 in anatase at room temperature.^[^ ^142^, ^143^ ^]^ This kind of behavior was also reported in titania nanotubes where Li_0.98_TiO_2_ was obtained in hydrothermally grown nanotubes and in hollow spheres of anatase.^[^ ^144^, ^145^ ^]^

Differently from the case of LMO‐type ion sieves, there are only two main LTO‐type structures that have been reported for lithium recovery: the spinel phase Li_4_Ti_5_O_12_, that present excellent cyclability and has been widely used in battery applications, and the layered titanate Li_2_TiO_3_.^[^ ^121^, ^146^ ^]^ This is probably due to the fact that the research of LTO‐type adsorbents is much younger than the one on LMO‐type adsorbents. In the following, several case studies of LTO‐type adsorbents will be analyzed based on their forming technique. The different advantages and disadvantages of each technique will not be discussed again since they already have been highlighted when illustrating the LMO‐type adsorbents.

##### LTO‐Type LIS in Powder Form and Granulated

Although one of the morphologies most studied for and most used in the field of lithium batteries is one of nanotubes, whether they are preferentially oriented or disordered, self‐organized or not, grown by anodic oxidation or hydrothermal treatment,^[^ ^130^, ^147^, ^148^, ^149^ ^]^ in the case of adsorption the powder form is still the most common one. Calcination conditions were extensively studied, as were the polymorphs of titania and their suitability for lithium recovery purposes.^[^ ^57^, ^58^, ^60^, ^62^, ^82^, ^150^ ^]^ The influence of the exposed facets has also been investigated.^[^ ^79^ ^]^ Although this kind of adsorbent can reach performances similar to the LMO ones, its processes are generally slower. Lower adsorptions were reported for adsorbents prepared by sol‐gel methods, which were ascribed to the finer size of the particles produced. This modified the adsorption process, becoming then a mix of surface adsorption and ion exchange.^[^ ^62^, ^151^ ^]^ Granulated adsorbents of calcinated Li_2_TiO_3_ and polyvinyl butyral (PVB) were recently reported by Zhang et al. reaching values of 25 mg_Li_ g^−1^ from 200 mg_Li_ L^−1^ LiOH solutions.^[^ ^66^, ^69^ ^]^ Lower adsorption values were also found when working with brines and the trend of higher adsorption in case of higher pH values, highlighted by Ooi et al.^[^ ^122^ ^]^ for LMO adsorbents, was confirmed for LTO adsorbents too.^[^ ^59^, ^60^ ^]^ Granulated adsorbents with either PVC or agar as binders were also tested in geothermal waters at higher temperatures. In this case, slightly higher adsorption capacities were reached with agar as binder.^[^ ^152^, ^153^ ^]^

Although widely used, hydrochloric acid is not the only eluent that can be employed. Various works reported the use of nitric acid, or persulfates of sodium and potassium to have a less aggressive environment for the ion sieve structure.^[^ ^77^, ^84^, ^154^ ^]^ Other powder morphologies were also explored, such as nanoribbons and nanotubes hydrothermally prepared and lithiated, that were able to reach impressive adsorption values of 160 mg_Li_ g^−1^ from a 2000 mg_Li_ L^−1^.^[^ ^63^, ^76^, ^78^ ^]^ Carbon microspheres with sea urchin‐like Li_4_Ti_5_O_12_ shell were also investigated. This yolk–shell structured ion sieve was reported as a very fast and performing adsorbent for lithium from aqueous resources.^[^ ^83^ ^]^ The particularity of such adsorbent mainly resides in the high equilibrium adsorption reached within 2 h, 28.46 mg_Li_ g^−1^ from a 500 × 10^−3^ m LiOH solution, which was ascribed to the presence of accessible surficial voids.

Finally, in a very recent work, Hossain et al. prepared LTO ion sieves with titania coming from dry sludge of a wastewater plant.^[^ ^67^ ^]^ These presented good adsorption and selectivity properties, reaching circa 35 mg_Li_ g^−1^ from a 115 mg_Li_ L^−1^ LiOH solution. Not only were these adsorbents prepared by sludges and therefore directly part of a circular economy frame of reference, they also present themselves as promising alternatives to commercial titania for this kind of application.

##### LTO Foam Adsorbents

Following the same concerns about the handling and recovery of adsorbents in the powder form as with LMO‐type ion sieves, forming techniques are starting to be studied for LTO‐type powders. A first study of solid‐supported metatitanic acid into a ceramic foam was conducted. However, LTOs prepared by sol‐gel method present a lower adsorption capacity, which was further decreased by the presence of the ceramic foam.^[^ ^80^ ^]^ More promising results were obtained when using PVA to obtain foams, rigid when dry but flexible when hydrated, loaded with LMO ion sieves.^[^ ^88^, ^89^ ^]^

Finally, a 3D macropourous–mesoporous foam containing the spinel Li_4_Ti_5_O_12_ was recently reported.^[^ ^64^ ^]^ The spinel phase was prepared through a combination of hydrothermal and low‐temperature calcination and formed into its inverse opal structure with a closely‐packed polystyrene microarray as hard template.^[^ ^62^ ^]^ The ion sieve thus prepared showed excellent adsorption properties, reaching 38.24 mg_Li_ g^−1^ in pure 50 × 10^−3^ m LiOH solution after 24 h, a much higher value than the one reported by the same adsorbent in its non‐porous form, which was only 7.77 mg_Li_ g^−1^.

Although easier to handle and to recover from the solutions after the batch experiments, LTO foams suffer from the same disadvantages as LMO foams. Their main advantage of having a macroscopic structure is indeed countered by a reduction in their adsorption capacity due to the presence of the binder which shields some adsorption sites. Further studies are therefore needed to investigate the role of the porosity and the choice of the binder.

#### 2.2.4 Mixed LMTO

Seeing as both LMO and LTO adsorbents present advantages and disadvantages, some researchers tried to combine them in a mixed oxide composite. An example of this was a titanium‐intercalated lithium manganese oxide composite prepared by calcination.^[^ ^70^ ^]^ The ion sieve thus obtained recorded an adsorption capacity of 15.8 mg_Li_ g^−1^ even after five cycles of adsorption in spiked seawater with 60 mg_Li_ L^−1^.

### 2.3 Membrane Techniques

Membrane techniques have attracted a lot of attention in the past years for their high separation efficiency and modular design that make them the most promising technique for industrial and large‐scale applications, but most of all for their versatility. Their adaptable nature is the main responsible for their widespread field of applications, which spaces from support for ion sieves in mixed matrix membranes (see Section 2.2.2.) to ultimately membrane‐assisted electricity‐driven processes (see Sections 3.1.1, 3.2.2, and 3.3.1).^[^ ^155^, ^156^ ^]^ The aim of this chapter is to analyze a few techniques and case studies that lie between those two extremis, namely the use of nanofiltration membranes and membrane distillation and crystallization. More information on membrane techniques can be found in Li et al. and Hou et al.^[^ ^156^, ^157^ ^]^

#### 2.3.1 Nanofiltration Membranes

**Figure 8:**
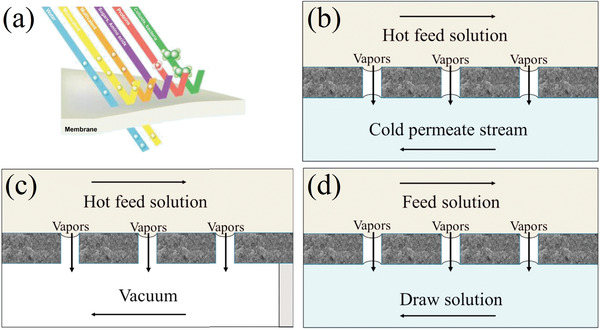
a) NF, basic configurations. Adapted with permission.^[^ ^43^ ^]^ Copyright 2020, Elsevier, b) DCMD, c) VMD, (d) OMD basic configurations. Adapted with permission.^[^ ^177^ ^]^Copyright 2016, Elsevier.

Nanofiltration (NF, **Figure** **8**a) is a pressure‐driven separation process that combines the Donnan effect, i.e., unequal distribution of permeant ions between the two sides of the boundary, and steric hindrance to selectively exclude divalent and multivalent ions.^[^ ^158^ ^]^ Up until now, NF membranes have been widely used in desalination processes as the last step before reverse osmosis,^[^ ^159^ ^]^ due to their molecular weight cut‐off ranging from 200 to 1000 Da. By combining size exclusion and electrostatic repulsion, this process has been also employed during water treatment for the retention of pollutants and pharmaceuticals.^[^ ^160^, ^161^, ^162^, ^163^ ^]^

Lately, their possible application to lithium enrichment in aqueous solutions has been investigated, the first attempt being reported in 2006 by Wen et al.^[^ ^164^ ^]^ Several works have investigated the use of charged nanofiltration membranes in different brines (Tunisian lake Chott Djerid, Chinese Taijinar lake). The results confirmed a better suitability of NF membranes with respect to reverse osmosis in terms of both permeability and selectivity. This kind of membrane was demonstrated to work well as a preprocessing step to enrich the lithium concentration of the feed stream and selectively remove magnesium ions. However, a second step of dialysis was also deemed necessary as this kind of membrane does not show selectivity between monovalent cations.^[^ ^165^, ^166^ ^]^ Positively charged NF membranes were also investigated and showed a superior attitude in the separation of Li^+^/Mg^2+^ ions in highly concentrated streams due to the Donnan exclusion phenomenon.^[^ ^167^, ^168^, ^169^, ^170^, ^171^, ^172^, ^173^, ^174^ ^]^

#### 2.3.2 Membrane Distillation and Crystallization

Membrane distillation (MD) and membrane crystallization (MCr) are two techniques that rely on the formation of a supersaturated solution by means of the vapor pressure gradient created across a microporous hydrophobic membrane.^[^ ^175^ ^]^ The main difference between the two resides in the output of the process: whereas in MD the result in a supersaturated solution, in MCr the solution, which can be the result of the previous MD, crystallizes and thus results in a solid specimen. The potentiality of MCr in seawater‐related processes such as water desalination is well known as it could theoretically reach the “zero‐liquid” discharge of the process and has been tested in Li^+^/Mg^2+^ separation from brines and seawater.^[^ ^176^ ^]^ In their study from 2016, Quist‐Jensen et al. conducted a comparative analysis of MCr conducted in different MD modes to recover LiCl:^[^ ^177^ ^]^ direct contact membrane distillation (DCMD), vacuum membrane distillation (VMD) and osmotic membrane distillation (OMD) with two polypropylene (PP) commercial membranes (basic configurations are shown in Figure 8). VMD was the only technique that reached the super‐saturation needed to make LiCl crystallize.

More recently, Park et al. proposed a process comprising a nanofiltration membrane followed by a membrane distillation step as an alternative to traditional evaporation for lithium enrichment and removal of undesirable ions.^[^ ^43^ ^]^ In particular, the NF step allowed for an easy and green removal of divalent ions that would otherwise crystallize during the MD process. By combining these two processes the brine was concentrated from 100 mg_Li_ L^−1^ to 1200 mg_Li_ L^−1^ after 140 h, not only reducing the time of the whole lithium production process from 12 to 24 months to just 1 to 2, but also decreasing the usage of chemicals and the footprint of the whole operation.

### 2.4 Supramolecular Systems

#### 2.4.1 Supramolecular Chemistry of Crown Ethers (CE)

Before its recovery from aqueous resources, lithium has been at the center of several researcher's interest as its detection and quantification in aqueous solutions was of capital importance in monitoring patients suffering from dementia‐related conditions. The field of lithium‐ion sensors has therefore been flourishing since many decades, and lithium ionophores are only part of it.^[^ ^178^ ^]^ Among ionophores, heterocycles like crown ethers are of particular interest for their ability to selectively bind with ions. Although the first synthesis of the 12‐crown‐4 ether (12C4E) dates to Steward's patent of 1957, it is not until Pedersen's ground‐breaking study of 1967 that these heterocycles were appropriately studied and named.^[^ ^179^, ^180^ ^]^ In particular, Pedersen noticed the correlation between the cavity of the cyclic structure and the cation to whom it could selectively bind. Shortly after, the first reports of selectivity of lithium ionophores either in membranes or as extracting agents started to arrive. Although the first ones use ionophores that are not crown ethers, like Kirsch et al. and Zhukov et al., they are still worth mentioning in this context as they are the first attempts of lithium selective extraction with this kind of structures.^[^ ^181^, ^182^ ^]^

From there, studies about the lithium selectivity of crown‐4 derivatives and the synthesis of compounds with added functional groups to improve the selectivity or the stability quickly followed. Kitazawa et al. reported in 1984 the synthesis of 13‐ to 16‐ crown‐4 member rings with long aliphatic chains, Kimura et al. their application for extraction photometry when presenting a phenol chromogen group in 1985 and for serum lithium assay with amide groups and/or bulky substituents in 1987.^[^ ^183^, ^184^, ^185^ ^]^ The use of lariat ethers, i.e., crown ethers presenting a branched arm responsible for 3D solvation, and their selectivity was also investigated.^[^ ^186^, ^187^ ^]^ Theoretical models were also realized to investigate the interactions between the crown‐4 ethers and the various cations.^[^ ^188^, ^189^, ^190^, ^191^, ^192^, ^193^ ^]^ The importance of this class of compounds and its versatility in the study of sensing and recovering lithium from aqueous solutions is witnessed by the amount of both theoretical, experimental. and literature works that is still performed to this day to better understand its potentialities.^[^ ^178^, ^194^, ^195^, ^196^, ^197^, ^198^, ^199^, ^200^ ^]^ In the following, several examples of different materials and morphologies of lithium adsorbents employing mostly 12‐crown‐4 ether for lithium recovery are presented.

#### 2.4.2 Ion Imprinted Polymers

Ion imprinted polymers (IIPs) can be considered the organic counterpart of ion sieves. Those selective sorbents are composed of functional monomers, template ions, and cross‐linkers, the functional monomers often being crown ethers. The similarity with ion sieves derives from the fact that the target ion can be inserted and extracted from the functional monomers without modifying the overall structure, just as it was in inorganic spinel structures.

**Figure 9:**
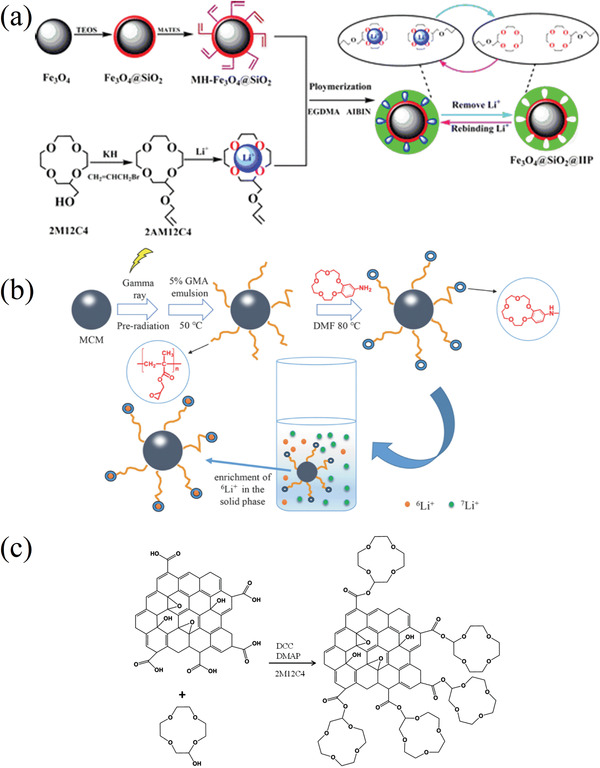
a) Synthetic route of the Fe_3_O_4_@SiO_2_@IIP. Adapted with permission.^[^ ^202^ ^]^ Copyright 2015, American Chemical Society. b) Synthetic route of cellulose microspheres functionalized with azacrown ethers. Reproduced under terms of the CC‐BY license.^[^ ^204^ ^]^ Copyright 2019, The Authors, published by MDPI. c) Functionalization of GO flakes via carbodiimide esterification. Reproduced with permission.^[^ ^213^ ^]^ Copyright 2018, Centre National de la Recherche Scientifique (CNRS) and the Royal Society of Chemistry.

In this configuration, IIPs can be grafted onto support morphologies to make their recovery from aqueous solutions easier. Adapting the procedure from Zhang et al. to synthesize core–shell magnetic Fe_3_O_4_@SiO_2_@IIP, Luo et al. realized Fe_3_O_4_@SiO_2_@IIP with 12C4E to selectively bind with lithium and recover it from wastewater of LIBs dismantle.^[^ ^201^, ^202^ ^]^ The synthetic route employed is shown in **Figure** **9**a. The adsorbents thus prepared were tested at pH 6 reporting a maximum adsorption capacity of 4.06 mg_Li_ g^−1^ from a solution with 104 mg_Li_ L^−1^. The same structure was also successfully tested in mixed solutions for a parallel recovery of two different target ions, lithium and rubidium. In this case, two different crown functionalizations were performed.^[^ ^203^ ^]^ A similar idea but with different materials was realized by Chen et al. who functionalized cellulose microspheres with azacrown ethers (Figure 9b).^[^ ^204^ ^]^ As it was already pointed out, several properties of cellulose like its nontoxicity and biodegradability have made this natural polymer a promising candidate for this kind of application.^[^ ^91^ ^]^ The azacrown‐modified cellulose was reported not only to selectively adsorb lithium but also to differentiate between the isotopes ^6^Li and ^7^Li. An uptake of 12.90 mg_Li_ g^−1^ was reached from a solution of 1 g_Li_ L^−1^ in acetonitrile, whereas lower uptakes and separation were reported for water solutions.

More recently, Zhao et al. proposed a magnetic IIP‐GO/Fe_3_O_4_@C adsorbent in which the presence of GO as framework material resulted in an increase of the adsorption sites. The crown ether was grafted onto the GO network by means of methacrylic acid and an adsorption capacity of 22.9 mg_Li_ g^−1^ was reached from a 100 mg_Li_ L^−1^ brine, with 91% of it retained after six cycles eluted with 0.5 m HNO_3_.^[^ ^205^ ^]^

#### 2.4.3 Ion Imprinted Membranes

As IIPs could be considered the organic equivalent of ion sieves, similarly ion imprinted membranes (IIMs) are the ones of mixed matrix membranes. In this case, functional monomers and target ions are embedded in the macroporous membrane in order to realize highly selective adsorbents that can be easily regenerated and requiring low‐energy processes. As with all membrane‐related processes, there is a wide choice of possible membrane materials. In the following we present some case studies reporting the use of graphene oxide (GO), PVDF, and polyethersulfone.

The possible use of GO membranes in water treatment and their exploitation in nanofiltration processes, forward osmosis and desalination is well known.^[^ ^206^, ^207^, ^208^, ^209^ ^]^ GO can either be used in this field as main material or as an additive. In the first case, its use is mostly motivated by its ionic and molecular ion sieve properties related to its porosity and layered structure.^[^ ^210^ ^]^ Its use as additive is instead generally used to improve antifouling properties, hydrophilicity and mechanical properties of the matrix.^[^ ^211^, ^212^ ^]^ The most common polymers added to GO to form membranes are PVDF, polyethersulfone, and PVA. An example of the first is the one reported by Sun et al.^[^ ^213^ ^]^ In this case, the authors functionalized pristine GO flakes with 2‐methylol‐12‐crown‐4 ether by carbodiimide esterification in dimethyl sulfoxide (DMSO, see Figure 9c) and added PVDF. They then prepared a membrane by phase inversion and tested it in H‐model‐tube setup with LiCl solution (50 mg_Li_ L^−1^), reporting a maximum adsorption capacity of 24.25 mg_Li_ g^−1^ and good stability after 10 cycles.

**Figure 10:**
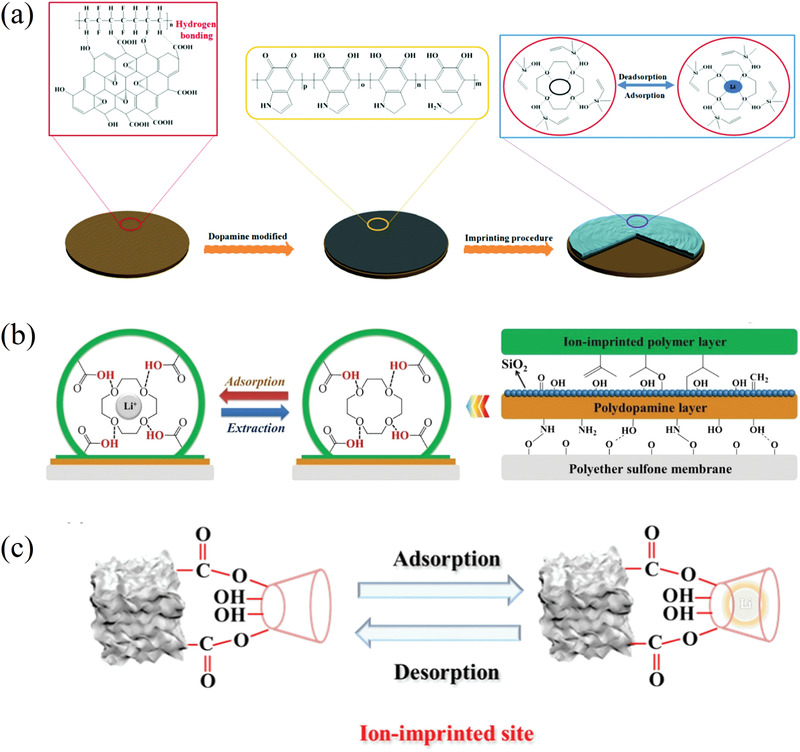
a) Synthesis of the hybrid membrane selective for lithium. Reproduced with permission.^[^ ^214^ ^]^ Copyright 2018, Centre National de la Recherche Scientifique (CNRS) and the Royal Society of Chemistry. b) Structure and synthetic route of the multi‐layered membrane. Adapted with permission.^[^ ^217^ ^]^ Copyright 2017, Elsevier. c) Adsorption mechanism of the calix[4]arene IIM. Adapted with permission.^[^ ^220^ ^]^ Copyright 2020, Elsevier.

Conversely, an example of PVDF membrane in which GO was later added is reported by Cui et al.^[^ ^214^ ^]^ In this case, the hybrid membrane was prepared via phase inversion and then polydopamine (pDA) was used as adhesion layer. The ion imprinting procedure was then carried out by hydrolysis polymerization of triethoxyvinylsilane (VTES) with the crosslinking agent tetraethyl orthosilicate (TEOS) and 12C4E as lithium complexing agent (see **Figure** **10**a). The maximum adsorption capacity resulted to be 27.10 mg_Li_ g^−1^ from a solution of 200 mg_Li_ L^−1^ LiCl and a regenerating solution of 1 m HCl. A PVDF membrane modified via pDA as adhesion layer for 12C4E was previously reported by Sun et al.^[^ ^215^ ^]^ In that case, experimental setup consisted in a H‐model tube, and the maximum adsorption reported was of 27.10 mg_Li_ g^−1^ from a solution of 200 mg_Li_ L^−1^ LiCl. Zheng et al. proposed a crown ether functionalized GO+PVA nanofiber membrane that could adsorb 97.23 mg_Li_ g^−1^ from a 1000 mg_Li_ L^−1^ solution and retained almost 90% of its ability after five cycles eluted with 500 × 10^−3^ m HCl.^[^ ^216^ ^]^

A further example of Li‐IIM is the multilayered polyether sulfone membrane proposed by Lu et al.^[^ ^217^ ^]^ The structure of this membrane can be seen in Figure 10b. Polyether sulfone was chosen as support membrane for its high porosity, stability, and pressure resistance. A pDA adhesion layer functionalized with silica nanoparticles to enhance the hydrophilicity and stability of the membrane was finally ion‐imprinted with 12C4E. The membrane was tested in a solution of 50 mg_Li_ L^−1^ LiCl and reported a maximum adsorption of 27.55 mg_Li_ g^−1^ and good regeneration capacity, keeping the rebinding capacities around 90% after five cycles. A different approach was used by Bai et al., who instead realized polymeric brushes of 2‐methylol‐12C4E and grafted them on a polymeric high internal‐phase emulsion foam by UV‐initiated surface polymerization. This allowed the crown ethers to bond more strongly and thanks to its open structure the adsorbent was able to reach equilibrium after only 45 minutes. However, its uptake resulted lower than those reported by other foams.^[^ ^218^ ^]^ In a different approach, Cheng et al. proposed a crown ether functionalization of a chitosan nanofiltration membrane. The authors reported an adsorption of 297 mg_Li_ g^−1^ from a 1000 mg_Li_ L^−1^ solution and a good reusability and selectivity in the presence of other common cations.^[^ ^219^ ^]^

Lastly, we report the system of Yu et al. who instead of using a four‐ring cyclic ether used a different macrocyclic molecule, namely a calix[4]arene (see Figure 10c).^[^ ^220^ ^]^ Similar to what happened in the other IIMs analyzed, a pDA adhesive layer was used to anchor the functional group to the matrix, in this case constituted by polydimethylsiloxane network, but a layer of carboxyl groups was also used in combination with pDA. The superior adsorption properties of the calixarene IIM were attributed to the ester bond that the ligand formed with the target ion, instead of canonical hydrogen bonds as are encountered in crown ethers. However, the use of ethylenediaminetetraacetic acid (EDTA) as eluent and of high feed concentrations does not allow one to draw accurate comparisons with the other IIMs under investigation.

#### 2.4.4 Crown Ether and Hierarchical Porous Silica

A different morphology from the ones previously discussed is the hierarchical porous silica (HPS) functionalized with crown ethers presented by Xu et al.^[^ ^221^ ^]^ The sorbent in question was realized by dual‐template technique and later modified with 2‐methylol‐12C4E by vacuum filtration. The hierarchical structure presented increased diffusivity due to the various pore size and good mechanical strength resulting from the use of silica. Its selectivity and adsorption properties were investigated, resulting in 1.79 mg_Li_ g^−1^ adsorbed from a 100 mg_Li_ L^−1^ LiCl solution. The low adsorption capacity was attributed to the low amount of 12C4E (5.74 wt%) that was binded to the HPS structure.

### 2.5 Ionic liquids

#### 2.5.1 Liquid–Liquid Extraction

Liquid–liquid extraction, or solvent extraction, has been extensively used in the past years for chemicals and metals production and is one of the most important hydrometallurgical processes. The operational principle of liquid‐liquid extraction is the following. When faced with two mutual immiscible phases, namely an aqueous one and an organic one, metal ions will present a preferential distribution. Furthermore, dissolved metal ions can be moved from one phase to the other by means of an extracting agent. In the past years, the use of ionic liquids as organic phase has been widely investigated.^[^ ^222^ ^]^ Their main advantages are their very low vapor pressure, which would entail safer and greener processes. It is also possible to substitute both phases with ionic liquids mutually immiscible, making the whole process more selective.^[^ ^223^ ^]^ Unfortunately, ionic liquids can be very expensive because their synthesis is still mostly at a laboratory scale, which makes wide industrial applications difficult.

As with other metals, liquid–liquid extraction (LLE) can be used for the recovery of lithium, with or without the use of ionic liquids. In the work of Torrejos et al. an environmentally friendly LLE system comprising a room temperature ionic liquid (RTIL) as organic diluent and a modified crown ether as extracting agent was presented.^[^ ^224^ ^]^ The dibenzo‐14‐crown‐4 ether molecules were modified with a carboxylic acid arm in order to have the necessary proton ionizable functional group, and a long C_18_ alkyl chain improved the lipophilicity of the structure. The RTIL in which the extractant was dissolved was CYPHOSIL 109, due to its low solubility in water and minimal elution of the extractant in the aqueous phase.

A more affordable process was proposed by Wang et al. using tributyl phosphate (TBP) as extractant and a fluorine‐free heteropolyacid ionic liquid as co‐extractant.^[^ ^225^ ^]^ While the first is the most common extractant for LLE of lithium, the latter was chosen in reason of its high hydrolysis, high catalytic activity and thermal stability which avoid secondary pollution of the brine. The extraction efficiency reached 99.23% after 5 cycles and decreased when increasing the length of the carbon chain, responsible of the rise of hydrophobicity and viscosity. The suitability of such a system for brines with high Mg:Li ratio was highlighted when Mg was precipitated with a washing solution of 926 × 10^−3^ m NaCl + 252 × 10^−3^ m LiCl, before the stripping with 300 × 10^−3^ m HCl to make Li_2_CO_3_ precipitate.

#### 2.5.2 Supported Liquid Membrane

Ionic liquids can also be used to impregnate support membranes, becoming then supported liquid membrane (SLMs), which have been used for pre‐concentration or selective removal of metal ions from aqueous solutions. The configuration of SLMs is different from the conventional LLE with ionic liquids, as three phases are present at the same time: the feed solution, the stripping one and the organic phase of the SLM. In the work of Zante et al. a PVDF membrane was used as support and the organic phase was 90% TBP 10% 1‐Butyl‐3‐methylimidazolium bis(trifluoromethylsulfonyl)imide ([C4mim][NTf_2_]).^[^ ^226^ ^]^ The stability of the membrane was investigated, noting how the presence of salts in the aqueous phase could enhance the stability of the SLM. The stripping phase was then chosen to be of sodium carbonate so as to combine extraction, stripping and precipitation in a single operational step.

As it was previously outlined, the efficiency and capacity rate of the passive processes, and therefore their application, are mainly limited by two factors. The first one is the relatively low diffusion rate of lithium ions in aqueous solutions. This is the reason for which a preconcentration can be considered as a complementary step to the lithium recovery process and for which several studies include the evaluation of temperature and pH effects on adsorption. The second aspect is the moderate ion‐exchange rate of lithium ion in the adsorbents. This is considered the main reason for the necessary long times of adsorption. A possible countermeasure to those inherent characteristics of the passive processes can be seen in the application of a driving force (i.e., flow of current, electrical potential difference). The response of the system to the presence of the electrical current is typically the variation of the entropy by the presence of alternative reactions (e.g., ion‐pumping, membrane hybrid capacitive deionization) and ion's migration (e.g., electrodialysis).

## 3 Processes for Li Extraction: Electrochemical Processes

The concept behind electrochemical processes such as ion‐pumping technologies is the presence of a redox couple or a redox reaction, which are driven by the changes in the electric field at the interface, and which can be coupled to the adsorption or absorption of lithium ions. Complementary, by the implementation of electrochemical‐driven processes, it is no longer necessary to use chemicals such as strong acids to elute lithium from the active materials, thus lowering the environmental impact of the whole process.^[^ ^47^, ^227^, ^228^, ^229^ ^]^ As stated above, these electrochemical processes for lithium extraction have in common the need of operating under a current flow. Concisely, they consist in the removal of lithium from a feed solution using redox reactions or polarization of the electrodes and subsequent release of the ions (i.e., regeneration of the electrodes) in a recovery solution via switching the current density direction, reversing the cell voltage or short‐circuiting the cell.^[^ ^230^ ^]^ The electrochemical methods have emerged as promising approaches to recover lithium from water resources, not only because they offer better performance metrics such as higher lithium removal capacities and efficiencies, but also thanks to the following intrinsic characteristics: 1) reduction of the chemical wastes; 2) flexible operation modes allowing a better control of lithium production rates and smaller water consumption and 3) low energy consumption, as a result of their energy efficiency and the reversibility of the processes taking place in the electrodes.

**Figure 11:**
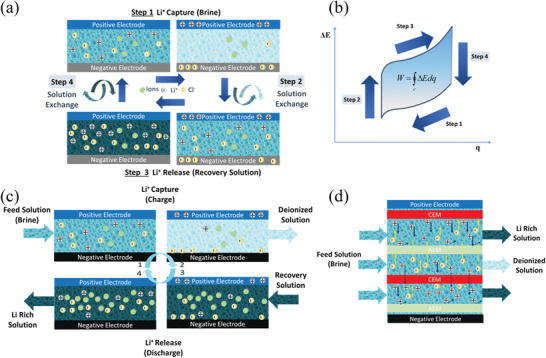
Diagrams of electrochemical methods based on a,b) ion‐pumping. c) Capacitive deionization (CDI) and d) electrodialysis (ED). a) Schematic representation of the working principle behind a complete cycle of ion‐pumping: Step 1, Li^+^ capture via battery‐based electrode material (e.g., LMO); Step 2, removal of the deionized water and inlet of recovery solution; Step 3, discharge of Li^+^ and anions in the recovery solution; Step 4, solution exchange to new brine. b) Typical form of an ion‐pumping cycle: cell voltage (Δ*E*) versus charge (*q*) in the ion‐pumping, demonstrating the energy consumed. c) Schematic representation of a CDI‐based system for Li removal where Li^+^ is removed via intercalation in the positive electrode whereas EDL formation takes place in the negative electrode (capacitive electrode such as activated carbon). d) Schematic representation of an ED system alternating ion‐exchange membranes between two polarized electrodes.

Recent critical reviews on the electrochemical methods for lithium extraction have been published. Further and more detailed information on these processes can be found in Calvo, Battistel et al., Zhao et al., Zhang et al., and Gmar.^[^ ^25^, ^231^, ^232^, ^233^, ^234^, ^235^ ^]^ In this review, we have classified the electrochemical processes based on the mechanism with which the ion is captured (see **Figure** **11**). Accordingly, the main electrochemical systems used to recover lithium from water resources based on 1) battery‐like electrodes (ion‐pumping), 2) asymmetric hybrid capacitors (HCDI, hybrid capacitive deionization) and 3) electrodialysis (ED), will be reported and discussed in the following paragraphs. It is worth to highlight that, recently, two novel electrochemical methodologies were proposed in literature: lithium extraction using ion concentration polarization and a redox‐mediated lithium removal.^[^ ^236^, ^237^ ^]^

### 3.1 Battery Based: Ion Pumping

**Figure 12:**
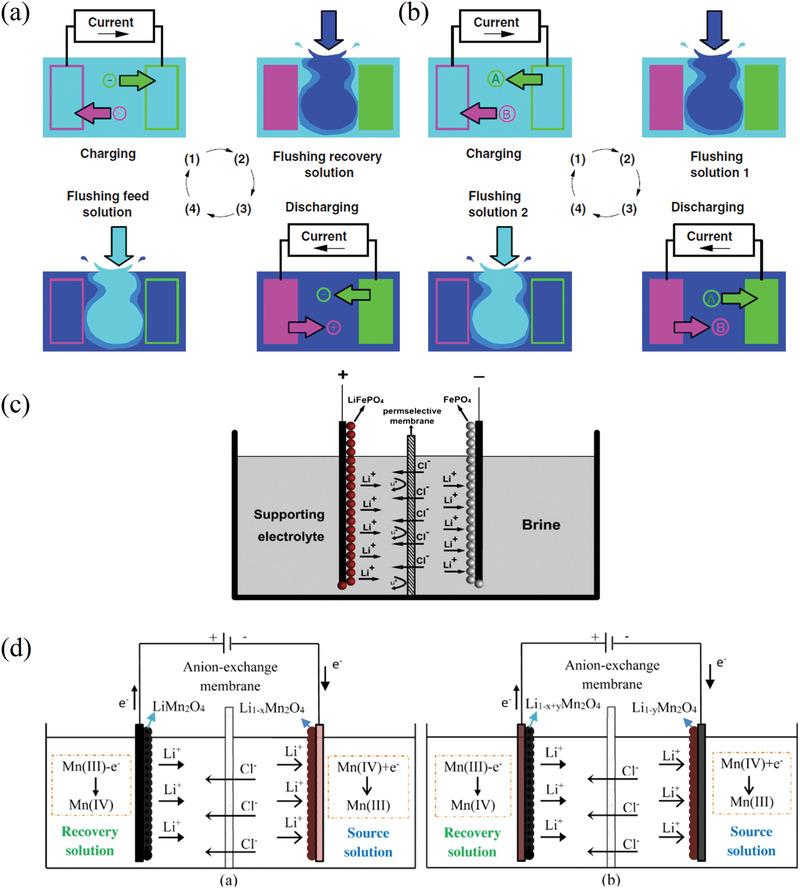
Schematic representation of electrochemical methods based on ion‐pumping depending on the charge compensation strategy a) salt capturing electrodes (e.g., Ag); b) ion exchange electrodes (e.g., NiHCF). Reproduced with permission.^[^ ^270^ ^]^ Copyright 2019, IOP Publishing. Schematic diagram of the electrolytic cells based on rocking chair ion‐pumping. c) LiFePO/FePO. Reproduced with permission.^[^ ^253^ ^]^ Copyright 2013, Elsevier. d) LMO/MO Reproduced with permission.^[^ ^257^ ^]^ Copyright 2017, Elsevier.

The first attempt of electrochemical recovery of lithium based on a battery‐like mechanism was reported by Kanoh et al. who, after studying the selective intercalation of an ion into a Pt/*λ*‐MnO_2_ electrode also performed the recovery of lithium from geothermal water with the same type of electrode.^[^ ^238^, ^239^ ^]^ Since the pioneering work of Kanoh, electrochemical extraction of lithium has attracted a lot of attention due to its environmental friendliness and its energy efficiency. In 2012, the term electrochemical ion‐pumping was established by Pasta et al. to refer to this electrochemical method.^[^ ^240^, ^241^ ^]^ Depending on the charge compensation strategy to guarantee the electrochemical neutrality while lithium is intercalated/deintercalated, several strategies have been reported in literature: a) ion capturing electrodes, where Cl^−^ ions react at the negative electrode, e.g., Ag or electroactive polymers (**Figure** **12**a), and are captured/released simultaneously to the lithium ions; b) Li exclusion electrodes such as Prussian blue analogues like nickel hexacyanoferrate (NiHCF) which release/capture other cations while lithium is captured/released (Figure 12b). Both strategies can be adopted to maintain the electroneutrality of the solution. As reported by Trócoli et al., these two strategies result in significant differences in the energy consumption and Li removal performance. While the ion exchange electrodes resulted in higher purity and efficiency, the salt capturing electrodes showed less energy consumption.^[^ ^242^ ^]^

It is interesting to notice that the energy efficiency of the process depends not only on the nature of both electrode materials, but also on the cell configuration and operational modes, as it often occurs in electrochemical systems. In the following paragraphs, some examples of the most common active materials used as electrodes will be analyzed. We will then focus our attention on some specific cases of cells with particular architectures.

#### 3.1.1 Electrodes

In ion‐pumping techniques for lithium removal both electrodes, the one responsible for Li‐intercalation and the one dedicated to the capture of negative ions, play an important role in the overall electrochemical performance. The former, i.e., the working electrode, is generally based on active materials already used either as adsorbents or in batteries. The composition of the latter, i.e., the counter electrode, is traditionally based on platinum or silver.^[^ ^238^, ^241^, ^243^ ^]^ However, the use of conductive polymers, Prussian blue analogues and bismuth has also been recently investigated.^[^ ^230^, ^244^, ^245^, ^246^, ^247^ ^]^ It is important to note that electroactive materials should be stable in water, present their electrochemical activity within the stability window of aqueous solutions, and show a high lithium selectivity toward other ions such as Na^+^, Mg^2+^ found in the brine (see Table 2).

##### Olivine Electrodes

The use of lithium iron phosphate, a phospho‐olivine, LiFePO_4_ (LFP) as a positive electrode material for lithium‐ion batteries was first proposed by Padhi.^[^ ^248^ ^]^ Differently from spinel structures, olivine ones present two octahedral sites, different both in size and from a crystallographic point of view. In the case of LiFePO_4_ it was experimentally proven, that the maximum extraction is 0.6 lithium atoms per formula unit, while being reversible at 3.5 V versus lithium with a slight increase upon cycling. The hcp unit cell of LiFePO_4_ presents a 1‐D diffusion channel that allows for an easy intercalation of lithium ions into the octahedral positions, while having only minor structural changes. The use of this material for low‐power batteries was therefore implemented as low price, non‐toxic, and environmentally friendly. In the case of electrochemical systems for lithium recovery, LFP is employed as a lithium capturing, positive electrode. The selective lithium extraction takes place through Equation 1:

(1) $$\mathrm{FeP}O_{4}+xLi^{+}+xe^{-}↔x\mathrm{LiFeP}O_{4}+\left(1-x\right)\mathrm{FeP}O_{4}$$

During the reduction from Fe(III) to Fe(II), lithium ions are intercalated forming the characteristic olivine structure of the LFP. Following during the oxidation of LFP, lithium is released in the electrolyte.

Different negative electrodes have been reported in literature: silver (Ag), nickel hexacyanoferrate (NiHCF), and FePO_4_. The former is used in the battery system proposed by Pasta et al.^[^ ^241^ ^]^ This study is the first work proposing an entropy concentration cell for lithium recovery by combining elements of mixing entropy batteries and desalination batteries.^[^ ^240^, ^249^ ^]^ The working principle of this system is shown in Figure 12a (see also Figure 11b). The capture of lithium ions during the first step of operation is thermodynamically favorable and thus the battery releases energy. In the third step, the lithium release takes place consuming energy. The even steps, instead, consist of a mechanical exchange of the solution. The setup resulted to be energy efficient, showing an energy consumption of 144 Wh kg^−1^ of lithium when converting a sodium‐rich solution of (Na:Li = 100:1) to Na:Li = 1:5 under 0.5 mA cm^−2^. However, the use of an expensive electrode like Ag was not ideal. Nevertheless, a noticeably high purity of lithium (ca. 99.9%) with other cations presented in a synthetic Atacama's brine was reported for this LFP/Ag system.^[^ ^250^ ^]^

The viability of LFP as an electrode to remove lithium was further studied in a similar setup, but replacing Ag by NiHCF.^[^ ^251^ ^]^ The use of this open‐framework material, with a crystal structure similar to Prussian blue, as electrode has already been explored in sodium and potassium ion batteries.^[^ ^252^ ^]^ The use of NiHCF as lithium exclusion electrode (Figure 12b) resulted in higher capacity but also higher energy consumption as well as a lower purity of the recovery solution. The introduction of potassium ions in the system was necessary to maintain the electroneutrality, but their effects were quantifiable, and this electrode proved to be a cost‐effective and environmentally friendly alternative to Ag. This entropy concentration cell has been widely studied recently, focusing on minimizing the enthalpic terms and exploiting the entropic ones.^[^ ^242^ ^]^

In a different approach to balance the charge during the selective lithium removal step, Zhao et al. reported an electrolytic cell based on the rocking chair mechanism using LiFePO_4_/FePO_4_.^[^ ^253^ ^]^ The positive electrode was prepared following the procedure proposed in a previous study,^[^ ^254^ ^]^ and the negative one was obtained by electrochemically pretreating, i.e., extracting lithium ions, from the positive electrode. The cell was tested with brine from the West Taijinar Salt Lake (China, 220 mg_Li_ L^−1^), whereas 500 × 10^−3^ m NaCl was used as a recovery solution. The process involved the negative electrode adsorbing lithium ions from the brine, while the positive one desorbed lithium ions in the recovering solution. Afterward the electrodes were switched to perform again the same cycle (see Figure 12c). While working at 1.0 V for 600 min, a lithium removal capacity of 41 mg_Li_ g^−1^ was obtained, i.e., 93.7% of its theoretical value, while the recovery one was around 34 mg_Li_ g^−1^, i.e., 94.3% of its theoretical value. However, when increasing the magnesium content in the solution to a ratio of Mg:Li being 60, the removal capacity of lithium dropped to 28 mg_Li_ g^−1^.

Despite the lower lithium insertion potential and high theoretical capacity, electrodes based on LFP suffer from low stability upon cycling and thus, the recyclability of the electrodes is thus limited.^[^ ^255^ ^]^ Recently, Wang et al. analyzed the fast degradation of LFP electrodes when removing lithium.^[^ ^256^ ^]^ In their study, they proposed two strategies to improve the stability performance of their electrodes: flushing the brine with nitrogen to minimize the oxygen content of the electrolyte and coating the LFP particles with a carbon shell. Both methods showed an improvement in the capacity retention from ca. 50% to 70–80% while keeping a lithium extraction capacity of 21 mg_Li_ g^−1^ with an energy consumption of 3 Wh mol_Li_^−1^ from a diluted brine (5 × 10^−3^ m LiCl + 50 × 10^−3^ m NaCl).

##### LMO Electrode

Lithium manganates, LiMn_2_O_4_ or *λ*‐MnO_2_ (LMO), are not only widely used adsorbents but also cathode materials for the electrochemical recovery of lithium. This cathode material is generally preferred over the olivine one because of its better stability, higher capacity retention, and good selectivity against not only monovalent cations but also against Mg^2+^.^[^ ^47^, ^255^ ^]^ The theoretical maximum amount of lithium recovered from this kind of electrode is circa 39 mg_Li_ g^−1^ and its theoretical specific capacity 147 mAh g^−1^.^[^ ^232^, ^257^, ^258^ ^]^

In the electrochemical extraction of lithium, the intercalation of lithium occurs inside the tetrahedral positions of the *λ*‐MnO_2_ lattice during the reduction of manganese (Equation 2):

(2) $$Li_{1-x}{\mathrm{Mn}}_{1-x}^{\mathrm{III}}{\mathrm{Mn}}_{1+x}^{\mathrm{IV}}O_{4}+xLi^{+}+xe^{-}↔x\mathrm{LiM}n^{\mathrm{IV}}Mn^{\mathrm{III}}O_{4}$$

In that first work, Kanoh et al. proved that their setup based on *λ*‐Mn_2_O_4_/Pt could harvest lithium from LiCl solutions with concentrations higher than 10 × 10^−3^ m, while the intercalation in the electrode did not take place with concentrations lower than 0.1 × 10^−3^ m. However, by running intercalation/extraction cycles in geothermal water (0.75 × 10^−3^ m) under a scan rate of 0.1 mV s^−1^, the authors reported a extraction capacity of 11 mg_Li_ g^−1^, which is comparable to the adsorption capacities of some of the most common adsorbents in passive processes.^[^ ^238^, ^239^ ^]^ Several years later, in 2013 Lee et al. continued to shed light on the knowledge of positive electrodes made of LMO for the selective electrochemical removal of lithium.^[^ ^243^ ^]^ They reported a selectivity of ≈30% consuming 1 Wh mol_Li_^−1^ to produce a solution with a lithium purity of ca. 90%. Higher purities (ca. 96%), while maintaining remarkable values of lithium selectivity (e.g., 57 and 1600 with respect to Na^+^ and Mg^2+^, respectively), were achieved by optimizing the crystallinity and the purity of the LMO electrode.^[^ ^255^ ^]^ In 2020, Kim et al. investigated a *λ*‐MnO_2_ electrode during an electrochemical lithium recovery process with the objective to provide insights about the important aspects to enhance its overall performance. They highlighted the importance of increasing the LMO particles/electrolyte interface in those cases in which the lithium concentration is small, to improve the degree of use of the active material.^[^ ^259^ ^]^

In a complementary action, several attempts to modify and enhance the lithium recovery performance when using LMO as active material were centered on the modification of the electrode composition. Although the use of binder to load the active material in the lithium selective electrodes is widespread, there are studies reporting electrodes based on LMO performance without using any binder or conducting additive. In 2016, Marchini et al. reported the lithium selectivity of a LMO electrode synthesized via pulsed laser deposition (PLD) or thermal decomposition. Using these thin‐film electrodes, they were able to study the limitation of Li^+^ adsorption sites due to the co‐adsorption of Na^+^.^[^ ^245^, ^258^, ^260^, ^261^ ^]^ Recently, a spinel *λ*‐MnO_2_‐based electrode prepared through cathodic deposition was proposed by Xu et al.^[^ ^262^ ^]^ In this work, the lithium removal capacity was studied in an aqueous solution of 100 × 10^−3^ m LiCl and then, the deintercalation of lithium was performed in a 10 × 10^−3^ m LiCl. A lithium extraction efficiency higher than 80% was reported after 100 cycles in the case of *λ*‐MnO_2_ without any binder. However, the capacity reported was relatively low and difficult to compare with literature values due to the lack of mass loading data for this type of electrode. Complementary, they claimed higher selectivity towards lithium recovery for this binder‐free electrode by studying the electrochemical performance in a simulated brine of 30 × 10^−3^ m LiCl, NaCl, KCl, MgCl_2_ and CaCl_2_. In this multi‐ion electrolyte, they also calculated an energy consumption of 4.14 Wh mol_Li_^−1^. It should be noted that this equimolar electrolyte is not representative for real brines in an applied scenario (see Table 2).

Studies described above concerning LMO electrodes included systems in which the charge was compensated by using counter electrodes such as Pt, Ag, NiHCF, or PPy. A different approach was proposed by Zhao et al. who suggested an electrolytic system with the rocking chair mechanism of Li*_x_* Mn_2_O_4_/Li_1‐_ *_x_* Mn_2_O_4_ and an anion‐exchange membrane to separate the two half cells.^[^ ^257^ ^]^ In this case both electrodes were prepared by spreading a slurry of 80 wt% LiMn_2_O_4_ prepared by calcination, 10 wt% carbon black, and 10 wt% of polytetrafluoroethylene (PTFE) onto carbon cloth. The negative electrode was then electrochemically pretreated to have a lithium deficiency. The peculiarity of this setup lies in the switching of the two electrodes between the two respective operation steps (see Figure 12d). During the first step, Li^+^ was adsorbed from the source solution (50 × 10^−3^ m LiCl + 50 × 10^−3^ m KCl) on the negative electrode side through the application of a constant potential. Following, the electrodes were switched, and the ions were released into the recovery solution (50 × 10^−3^ m NaCl). During this process, the extraction capacity reached 34.31 mg_Li_ g^−1^ at 1.2 V, which corresponded to 92.68% of the theoretical capacity and a current efficiency of 97%. The extraction was also performed from a 1:10 diluted brine from Salar de Atacama and concentrated seawater at 0.6 V with 50 × 10^−3^ m KCl as recovery solution. Here, the lithium recovery capacity resulted to be respectively 22.0 and 21.0 mg_Li_ g^−1^ with a current efficiency of 89% and 86%. The lower recovery capacities, namely 22% and 14% were attributed to the presence of impurities slowing down the process which nevertheless resulted in highly selective process towards lithium.

Recently, the same research group proposed the use of the LMO rocking‐chair configuration but working in a different operational mode.^[^ ^263^ ^]^ In this study, they introduce a first step where the spontaneous lithium removal from the brine (50 × 10^−3^ m LiCl + 100 × 10^−3^ m MgCl_2_) was done by short‐circuiting the electrodes. Then, a constant voltage was applied to increase the removal capacity. Several configurations changing the time of both processes were analyzed to find the optimal combination of those steps. When operating under short‐circuit for 1 h followed by 2 h at 0.6 V, they reported a lithium removal capacity of 33.5 mg_Li_ g^−1^ after six cycles consuming 7.63 Wh mol^−1^.

Thanks to the growing interest in lithium recovery by electrochemical methods, there has been an extensive development of LMO‐based electrodes to selectively remove lithium. More information about the electrochemical lithium recovery studies exploring the use of LMO electrodes is gathered and discussed in the short review of Joo et al. published in 2020.^[^ ^264^ ^]^

##### Counter Electrodes

As discussed above, there are different ion‐pumping approaches depending on the mechanism involved to guarantee the electroneutrality of the process (Figure 12). In fact, these possible configurations can be additionally differentiated from each other by the type of counter electrode used.

Besides the use of platinum as the counter electrode involving the oxidation and reduction of water, Ag electrodes are the most widespread among electrochemical methods (Figure 12a).^[^ ^49^, ^232^, ^240^, ^241^, ^249^, ^264^ ^]^ During lithium capture, silver electrodes are responsible for the reversible capture of chloride ions via the following reaction (Equation 3):

(3) $$\mathrm{Ag}+Cl^{-}↔\mathrm{AgCl}+e^{-}$$

The Ag/AgCl conversion reaction has been explored for the following reasons: the stability of both Ag and AgCl in aqueous media, and under a certain potential. However, silver‐based electrodes present some substantial disadvantages: the considerable high price of Ag, the toxicity of nanoparticles of Ag and dissolved Ag^+^, the limited electronic conductivity of the AgCl and the kinetic limitation of the subsurface AgCl conversion to Ag, concurrent to the formation of the silver oxide (side reaction).^[^ ^265^, ^266^, ^267^, ^268^ ^]^

Also following a conversion mechanism, bismuth‐based electrodes have emerged recently as an alternative to Pt and Ag electrodes to promote the selective Cl^−^ uptake (Equation 4).

(4) $$\mathrm{Bi}+Cl^{-}+H_{2}O↔\text{BiOCl}+2H^{+}+3e^{-}$$

To the best of our knowledge, two systems operating with bismuth have been published in 2021.^[^ ^246^, ^247^ ^]^ Niu et al. combined a lithium removal electrode based on *λ*‐MnO_2_ with a BiOCl‐polypyrrole (PPy) electrode.^[^ ^247^ ^]^ The electrochemical cell based on these electrodes showed a lithium removal capacity of 11 mg g^−1^ consuming 1 Wh mol_Li_^−1^ when lithium was extracted from an electrolyte of 100 × 10^−3^ m LiCl + 2.5 m Na_2_SO_4_. In the case of the system proposed by Zhao et al., the counter electrode was based on nanocrystalline bismuth electrodeposited on a titanium sheet.^[^ ^246^ ^]^ The lithium recovery electrode synthesized was a layered‐spinel heterostructure lithium‐rich material (Li_1.16_Mn_0.6_Ni_0.12_Co_0.12_O_2_). In this study, they reported an energy consumption of 1.8–4.5 Wh mol_Li_^−1^ to remove 1.88 mmol g^−1^ from an electrolyte of 23.48 × 10^−3^ m. Despite the encouraging results of these two electrochemical cells, more studies are needed to explore in detail the performance, versatility, and viability of bismuth‐based electrodes working as counter electrodes in lithium recovery devices.

As an alternative to conversion reactions to balance the charge during the electrochemical recovery of lithium, Prussian Blue derivatives have emerged as promising counter electrodes (Figure 12b). These electrodes are known as lithium exclusion electrodes and work under the following reaction (see Equation 5):

(5) $$\mathrm{MKNi}\left[Fe^{\mathrm{II}}{\left(\mathrm{CN}\right)}_{6}\right]↔M^{+}+\mathrm{KNi}\left[Fe^{\mathrm{III}}{\left(\mathrm{CN}\right)}_{6}\right]+e^{-}$$

Another possible strategy, based on chemical reactions, comprises the rocking chair mechanism (Figure 12c,d). Following, these exemplary equations (Equations 6 and 7) for the lithium in and deintercalation in LFP and LMO electrodes in combination with Equations 1 and 2, respectively:

(6) $$\mathrm{LiFeP}O_{4}\to x\mathrm{FeP}O_{4}+\left(1-x\right)\mathrm{FeP}O_{4}+xLi^{+}+xe^{-}$$

(7) $$\mathrm{LiM}n_{2}O_{4}\to Li_{1-x}Mn_{2}O_{4}+xLi^{+}+xe^{-}$$

Besides the use of Pt, Ag, or ion exclusion electrodes, electroactive polymers such as polyaniline (PANI) or polypyrrole (PPy) have been reported in literature as counter electrodes for lithium recovery.^[^ ^244^, ^245^, ^258^, ^260^, ^269^ ^]^ In this case, the mechanism is based on the charge compensation of positive charges exhibited in the conductive polymer backbone or in the structure of an organic molecule depending on their oxidation state (i.e., on redox reactions, see Equation 8).

(8) $$\begin{array}{c} Li_{x}Mn_{2}O_{4}\;+\;\left(1\;-\;x\right)\mathrm{PANI}+\mathrm{LiCl}↔\mathrm{LiM}n_{2}O_{4}\;+\;\left(1\;-\;x\right)\mathrm{PAN}I^{+}Cl^{-} \end{array}$$

In recent years, the group of Calvo and co‐workers realized several studies with PPy as counter electrode to avoid chlorine evolution that would occur with Pt. In fact, Marchini et al. in 2016 reported surface and bulk investigations on the properties of Li*_x_* Mn_2_O_4_ as a lithium deficient intercalation electrode and PPy as a reversible chloride electrode.^[^ ^231^, ^258^ ^]^ The LMO/PPy cell only had one chamber, while the aqueous solution was switched after extraction from the brine to the recovery solution after being washed with deionized water. The electrodes were tested both in a natural brine solution from Salar de Olaroz (Argentina, 141–186 × 10^−3^ m) and synthetic electrolytes. The authors demonstrated that by keeping the voltage range of the study between 0.4 and 1.1 V versus Ag/AgCl (in KCl 3 m) the stoichiometry of the Li*_x_* Mn_2_O_4_ could be kept with 0 ≤ *x* ≤ 1, intercalating lithium ions only in the tetrahedral sites of the spinel framework. The behavior of these electrodes was also tested in a sodium‐based electrolyte to investigate whether there could be sodium intercalation, since in natural brines the ratio Na:Li can reach 25:1. The comparison of the characterizations both in LiNO_3_ and NaNO_3_ however, confirmed that there was no sodium intercalation. The absence of sodium intercalation in LMO electrodes was further confirmed and the presence of sodium was deemed responsible only for blocking some of the lithium adsorption sites but did not enter the structure itself. ^[^ ^260^ ^]^ Whereas the non‐separated electrochemical cell of this study used a PPy electrode made by electrochemical polymerization on a platinum mesh, the one used by Missoni et al. was instead deposited on carbon felt.^[^ ^269^ ^]^ In this study, galvanostatic analyses were conducted in the same cell configuration of the previous work and reported a recovery efficiency of 50% with respect to the anodic charge and excellent stability over 200 cycles. A third study reported the spontaneous extraction of LiCl from natural brine both in a LMO/PPy cell and in a double cell Li_1‐_ *_x_* Mn_2_O_4_/LiMn_2_O_4_ with an anion‐selective membrane.^[^ ^245^ ^]^

#### 3.1.2 Electrochemical Cell and Reactor Configuration

From the perspective of a future industrial development of electrochemical lithium recovery systems, some key points are the electrochemical reactor design as well as the operational parameters. For the last several years, the scientific community has mainly focused on the active electrode materials, and only a very few efforts have been made to optimize the cell design and operation modes. The different options regarding the flowing conditions of the electrolyte, the impact of the mass loading of the electrodes, together with the optimization of operational conditions such as flow rates or current densities, play an important role in the performance metrics of the lithium recovery. The most important performance metrics to consider are the capacity, lithium recovery rate, cyclability, and the hydraulic energy needed. These aspects were widely discussed by Battistel et al.^[^ ^231^ ^]^ Moreover, in their review, they pointed out that electrochemical processes require the use of different electrolytes (i.e., feed and recovery solutions), and therefore emphasized the importance of providing a method to perform the complete cycle without having to manually move the electrodes.

**Figure 13:**
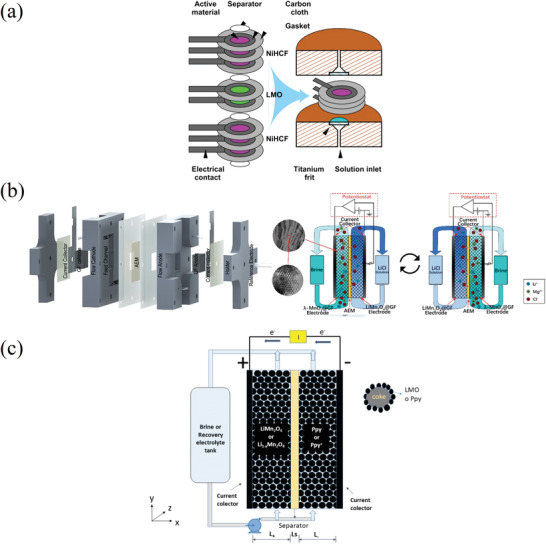
Schematic representation of different device configuration. Flow‐through cell based on a) carbon cloth electrodes and b) graphite felt electrodes. Reproduced with permission.^[^ ^230^ ^]^ Copyright 2019, Elsevier. Adapted with permission.^[^ ^273^ ^]^ Copyright 2020, Elsevier. c) Packed‐bed design. Reproduced with permission.^[^ ^276^ ^]^ Copyright 2021, IOP Publishing.

Depending on how the aqueous electrolyte flows into the cell, two different approaches have been reported: flow‐by and flow‐through. In the former case, the brine passes parallelly to the electrodes,^[^ ^271^ ^]^ whereas in the latter, the direction of the flow is perpendicular to the electrodes and thus passes through them.^[^ ^272^ ^]^ It can be generally stated that better lithium recovery performances could be achieved under the flow‐through configuration due to the mass transport improvement while reducing both the distance between the electrodes and the ohmic drop. Palagonia et al.^[^ ^230^ ^]^ reported a flow‐through‐electrode cell dedicated to the electrochemical ion pumping for lithium recovery, following a design first proposed by Trócoli et al.^[^ ^255^ ^]^ The electrodes involved in both cases were composed of LMO and NiHCF coated on carbon cloth as current collector (see **Figure** **13**a). The main difference with respect to their previous work consisted in the scalability and operational mode of the device proposed. The electrodes did not have to be moved from one cell for extraction to another for recovery, a simple change of the hydraulic connections was sufficient. In this case, the process involved a capture step by pumping the feed solution and applying −0.5 mA, followed by a cleaning step in which 120 × 10^−3^ m KCl was introduced into the chamber.^[^ ^230^ ^]^ The extraction with 120 × 10^−3^ m KCl under 1 mA of current was followed by a final cleaning of the cell by airflow. By pumping a feed solution of 1 × 10^−3^ m LiCl + 100 × 10^−3^ m NaCl for nine cycles, an overall coulombic efficiency of 75% and an extraction efficiency of 37% were reached together with a purity of 94%, recovering 3.45 mg of lithium from the original 9.3 mg of the source solution. The latter could be improved by lowering the current applied which limited the capture yield to 60% to reduce the time of the experiment. Thanks to the stability of the electrodes the system was also able to reach a final concentration of 100 × 10^−3^ m LiCl after nine cycles of performance.

Another reactor configuration based on flow‐through approach by using 3D electrodes was published in 2021.^[^ ^273^ ^]^ In this case, a graphite felt was impregnated with a standard electrode's slurry containing the active material, LMO (see Figure 13b). This procedure allowed to fabricate electrodes with high mass loadings (40 mg cm^−2^) that could also serve as turbulent promoters while guaranteeing the electrical connection thanks to the graphite felt structure. In this lithium recovery system, they calculated an energy consumption of 23 Wh mol^−1^ to remove lithium from a multicomponent brine (22 × 10^−3^ m LiCl + 41 × 10^−3^ m MgCl_2_ + 43 × 10^−3^ m NaCl + 26 × 10^−3^ m KCl), reporting a lithium removal rate of 75.06 mg_Li_ g^−1^ h^−1^ (24.02 mg_Li_ h^−1^). However, the energy consumption did not include the hydraulic energy for pumping the brine through the cell. For an adequate assessment of the energy required by this reactor using 3D electrodes, the hydraulic resistance of the cell must be provided.

In 2020, Joo et al. presented a pilot‐scale demonstration using *λ*‐MnO_2_/Ag electrodes to recover lithium from desalination concentrate.^[^ ^49^ ^]^ The system consisted of three steps, i.e., lithium adsorption, washing, and lithium desorption, and was directly connected to the desalination plant that could supply the desalination retentate (0.035 × 10^−3^ m Li) after processing it through reverse osmosis and membrane distillation. A first recovery step was followed by a secondary enrichment process. In that, the configuration with the stacks of electrodes moving between different solutions was switched in favor of a pair of stationary electrodes in a flow‐type reactor. Using 14 stacks of coupled electrodes the system enriched the lithium solution from 0.035 × 10^−3^ m to 62 × 10^−3^ m in the last step.

In addition to the reactor's configurations mentioned above and based on the previous work of Calvo et al., the challenges and key aspects of a flow reactor based on 3D porous packed bed electrodes (see Figure 13c) have been thoroughly studied by Romero et al. since 2018.^[^ ^274^, ^275^, ^276^, ^277^ ^]^ They developed and proposed two mathematical models (1D and 2D) including the effect of the diffusion and forced convection on the lithium extraction performance metrics, thus providing a tool to foresee the optimal operational parameters of their device. In their electrochemical reactor, the electrodes were based on petroleum coke particles coated with the two active materials already studied: LMO and PPy.^[^ ^274^, ^276^ ^]^ The reactor proposed, differed from usual hybrid systems (battery/supercapacitor electrode systems).^[^ ^274^ ^]^ In fact, it did not store any energy and the LiCl concentration in the electrolyte was not constant over the whole process. Instead, it would decrease during extraction and increase during recovery. They also claimed that this device could be powered by solar energy. The reactor was tested with a natural brine solution coming from Salar de Olaroz (Argentina) and 50 × 10^−3^ m LiCl was used as recovery solution. 1D mathematical models supporting the experimental results were developed under COMSOL environment. The concentration distributions of Li^+^, Cl^−^ and the nonintercalating ions were investigated. Additionally, the electrolyte potential and the solid‐electrolyte potential difference gradients were studied both in the separator and in the porous electrochemically active electrodes. Although the extraction resulted to be successful, the reactor presented several limitations with regard to a scale‐up of the setup. Namely, the PPy:LMO mass ratio proved to be a limiting factor to the charge of the device due to different specific charge capacities, together with a low extraction capacity of LMO (only 4%) and the ohmic drop of the electrolyte when the current density was increased. Pursuing this research, Romero et al. published in 2021 the second part of this study.^[^ ^276^ ^]^ In this work, they highlighted the importance of establishing a large PPy:LMO mass ratio, needed to maximize the efficiency of LiCl removal by balancing the capacity of both electrodes. It is worth mentioning that they developed a 2D mathematical model to describe lithium intercalation including forced convective flow conditions, diffusion, and migration of ions while operating in a natural diluted brine (Salar de Olaroz). Accordingly, voltage time evolution, concentration of all ions of the brine distribution, lithium ions concentration within LMO particles as well as electrical potential gradients evolution were also provided. They discussed the dependence of the extraction capacities on the flow conditions of their packed bed reactor showing flow rates ranging from zero to 54 mL min^−1^ that resulted in ca. 4 to 36 mg g^−1^_LMO_. Considering the main experimental and simulated results, they concluded that the optimal operation parameters were obtained under low current (matching the local insertion current density of the LMO) and that a high flow rate was needed to promote the diffusion of lithium within the porous structure. In their reactor, they showed that 1 mL min^−1^ was enough to overcome the diffusion concentration polarization.

They also recently investigated a packed bed reactor assembled with fully lithiated and partially de‐lithiated LMO (i.e., rocking chair mechanism) under two different flow configurations: flow‐through and flow‐by.^[^ ^275^, ^277^ ^]^ In these studies, the diluted natural brine employed was Salar de Hombre Muerto (Argentina). Potential and concentration profiles of ions as well as the time evolution of LiCl and ions were discussed. They found, experimentally and via COMSOL simulations, that the limiting factor in the lithium removal of these devices was the mass of the lithium deficient, Li_1‐_ *_x_* Mn_2_O_4_, electrode. Additionally, differences in the amount of LiCl extracted were complementary found: less LiCl was removed in the flow‐by configuration than in flow‐through. The worse performance of the flow‐by reactor was explained due to the smaller electroactive surface of the electrodes (i.e., in the flow‐by cell, the LMO was deposited as a thin film on the current collectors) that limited the amount of lithium extracted. They concluded that to promote lithium intercalation, a larger mass of active material and larger mass to volume of electrolyte ratio were required. Accordingly, the authors claimed that a flow‐through configuration, in which the electroactive surface of the electrodes is higher, was more suitable to extract lithium in a large scale.

### 3.2 Ion Electrosorption Methods for Li Recovery: CDI‐Based Systems

Capacitive deionization (CDI) is an electrochemical technique introduced in the 1960s to demineralize water using porous carbon electrodes. As explained by Blair and Murphy in their pioneering work,^[^ ^278^ ^]^ the process removes ions by electrosorption at low applied potentials, following an analogous process to that of regular supercapacitors. Its working principle is based on the accumulation of ions in the electrical double layer (EDL) at the surface of porous electrodes and their release when the polarization of the electrodes is reversed. Although being a desalination technique known for the past 60 years, CDI recently started to attract attention in the field of the selective removal of target contaminants from aqueous solutions and selective recovery of ions of interest (e.g., Li ions).^[^ ^279^ ^]^ CDI seems indeed to be an attractive candidate for this kind of operation, as its electrodes can be suitably tailored and membranes can be easily introduced to increase the selectivity of the setup. Furthermore, there is no need for high operating pressures and it shows a significant reversibility in the uptake of ions. However, its enormous potential requires increasing the ion removal capacity at low power and high energy efficiency. This performance metric depends on the processes taking place in the surface of the electrode material (i.e., electrolyte/electrode interface) and on the salinity range of the feed solution: currently CDI fails in its application for seawater desalination.

**Figure 14:**
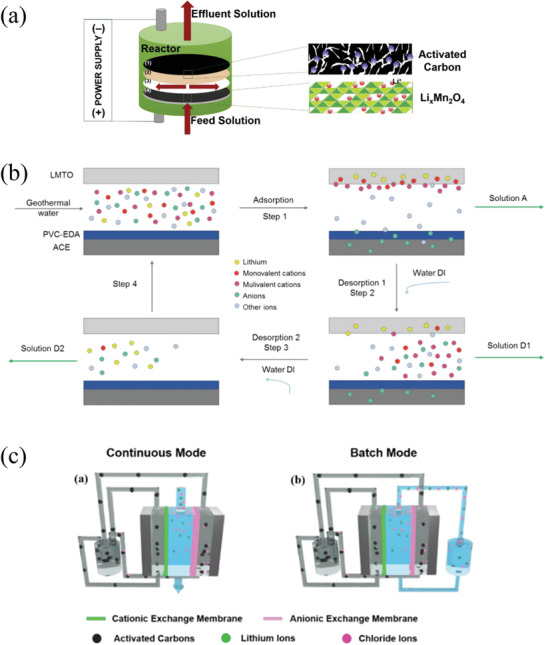
Schematic representation of electrochemical methods based on CDI. a) Diagram of the experimental setup of the hybrid supercapacitor system *λ*‐MnO_2_/activated carbon. Reproduced with permission.^[^ ^288^ ^]^ Copyright 2015, Elsevier. b) Operational mode of the HMCDI setup. Reproduced with permission.^[^ ^284^ ^]^ Copyright 2018, Elsevier. c) Schematic diagram of the flow electrode MCDI setup in (1) continuous and (2) batch mode operation. Reproduced under terms of the CC‐BY license.^[^ ^37^ ^]^ Copyright 2019, The Authors, published by MDPI.

The most common modifications of a CDI setup to improve its performance are 1) the use of ion‐selective electrodes (i.e., hybrid CDI, normally based on electrodes used in batteries and an activated carbon, AC)^[^ ^36^, ^37^, ^229^ ^]^ and/or 2) the use of an ion‐selective membrane (i.e., membrane CDI),^[^ ^279^, ^280^, ^281^, ^282^, ^283^, ^284^ ^]^ see **Figure** **14**a–c. While both strategies will be further analyzed in this review, a first comparison between the two of them was done by Bryjak et al., who proposed and studied two different CDI systems (HCDI and MCDI).^[^ ^227^ ^]^ In this work, a first CDI system was prepared with a battery‐liked electrode composed of 16 wt% *λ*‐MnO_2_ and 64 wt% AC, plus 15 wt% PVC and 5 wt% carbon black. The use of a conductive additive was meant to lower the resistance of the electrode and therefore to ultimately overcome the energy dissipation issues that were responsible for a lower adsorption capacity of LMO electrodes.^[^ ^229^ ^]^ The second system proposed was assembled with ion‐exchange membranes. These membranes were synthesized with a copolymer of acrylic acid‐co‐poly(glycidylmethacrylate) modified with hydroxyl‐methyl‐12‐crown‐4 ether. Both setups were tested in a 10 × 10^−3^ m LiCl solution and whereas the LMO electrode presented an adsorption capacity of 16.7 mg_Li_ g^−1^, the membrane showed a superior capacity of about 30 mg_Li_ g^−1^ because of the synergistic effects of the functional groups embedded in the membrane.^[^ ^227^ ^]^

To the best of our knowledge, there is only one publication which showed a CDI system removing lithium via electrostatic, i.e., without the need of membranes nor battery‐liked active materials.^[^ ^285^ ^]^ In this study, a lithium removal capacity between 10 and 37 mg_Li_ g^−1^ was reported from a 7 × 10^−3^ m electrolyte using electrodes based on oxygen vacancy‐rich CoP/Co_3_O_4_‐graphene aerogels.

#### 3.2.1 Hybrid CDI System: Battery Electrode/Activated Carbon

The use of hybrid CDI technology, HCDI, in which one of the electrodes captures ions by battery‐liked materials, can increase both the performance and selectivity of the CDI system.^[^ ^286^, ^287^ ^]^ In the practical implementation of HCDI for lithium recovery, this approach would imply on the one hand, the intercalation mechanism to remove lithium (i.e., lithium selective material like a mixed oxide for the positive electrode) and on the other hand, the formation of the electrical double layer (EDL) in a capacitive electrode to guarantee the electroneutrality of the process. This system therefore has a different approach compared to ion pumping processes regarding the counter electrodes used. In search of alternatives to Ag electrodes, HCDI usually employs a conventional counter electrode based on activated carbon (AC).

For instance, a *λ*‐MnO_2_/AC hybrid supercapacitor system was proposed by Kim et al.^[^ ^288^ ^]^ In this case, the positive electrode was a composite of 80 wt% LMO powder, 10 wt% carbon black and 10 wt% PTFE, whereas the negative one was 86 wt% AC, 7 wt% carbon black, and 7 wt% PTFE. In the reactor (Figure 14a) the negative electrode was composed of the AC and an anion exchange membrane, with a nylon spacer placed between the electrodes. The process consisted of two steps of 30 min each: during the discharge (−0.5 mA cm^−2^) lithium ions were adsorbed into the LMO electrode, whereas during the charge (0.5 mA cm^−2^) they were deintercalated. Several chloride solutions of 30 × 10^−3^ m were tested, and the lithium adsorption and extraction capacities were both over 90% of their theoretical value. When tested with a brine from Salar de Atacama (Chile) the system reported an efficiency comparable with that of silver‐electrode batteries, requiring 4.2 Wh mol_Li_^−1^.

Siekierka et al. reported a HCDI cell, in which the positive electrode was composed of 90 wt% LMTO and 10 wt% PVC and the negative one was a conventional 90 wt% AC with 10 wt% PVC one.^[^ ^36^ ^]^ The choice of adding 5 wt% titania to the LMO widely used in battery recovery systems was motivated by an improvement of the hydrophilicity of the electrode, and therefore an increased performance in aqueous solutions. The use of such an electrode proved not only to increase the adsorption capacity of lithium, which resulted to be 36.5 mg_LiCl_ g^−1^ from a 10 × 10^−3^ m LiCl solution, but also showed a relatively low energy consumption of approximately 8 Wh mol_Li_^−1^.

Recently, Shang et al. assembled a HCDI device with electrodes made by Li_3_VO_4_ on reduced graphene oxide (LVO/rGO) as the positive electrode, and AC as the negative electrode. They reported lithium removal capacities between 25 and 39 mg_LiCl_ g^−1^ from an 88 × 10^−3^ m solution, working with at constant voltage between 0.8 and 1.2 V applied for 1 h.^[^ ^289^ ^]^ Nevertheless, despite the importance of the energy assessment in CDI‐based systems, there were no energy calculations reported in their publication.

#### 3.2.2 Membrane‐CDI Systems

The use of membranes in CDI, MCDI, can greatly increase the efficiency of the system whether one talks about desalination processes or lithium‐ion removal/recovery.^[^ ^280^, ^290^, ^291^ ^]^ The presence of membranes adjacent to one or both electrodes enhances the transport of counter‐ions thus limiting the co‐ion repulsion effect that generally reduces the efficiency of the whole process.^[^ ^281^ ^]^ Several types of membranes, comprising ion exchange membranes, nanofiltration ones and also ion exchange resin coatings, have been used depending on the intended application.^[^ ^279^ ^]^ Mostly ion exchange membranes have been used for lithium recovery, as will be highlighted in the case studies considered in this review. Here we also discuss some cases in which anion exchange membranes are used together with a lithium selective electrode (Hybrid Membrane CDI, HMCDI),

A first example of HMCDI is the setup reported by Lee et al. in which the anion exchange membrane was placed in front of the negative electrode.^[^ ^282^ ^]^ In this case, the lithium selective electrode was a composite of inorganic spinel LMO prepared via solid‐state reaction, PVA, and glutaraldehyde, whereas the negative electrode comprised a mixture of AC and PVDF coated on a graphite sheet in addition with a commercial anion exchange membrane. The setup was studied to confirm the selectivity of the LMO electrode towards Li removal. The performance of the system was tested with a simulated brine from Salar de Atacama (Chile) both in the presence of an electric field and by means of pure physisorption. The former resulted to be seven times more efficient than the latter, recording an adsorption of 2.43 mg_Li_ g^−1^. However, the process proved to be more energy consuming than others, with 4.4 Wh g^−1^ during electrosorption and 23.3 Wh g^−1^ during desorption, most likely due to the use of DIW as electrolyte.

Another system in which the lithium selective electrode was a mixed oxide and an anion exchange membrane was coated on the cathode is the one proposed by Siekierka and co‐workers. In this work, lithium was extracted from geothermal water.^[^ ^37^ ^]^ The mixed oxide under examination in this setup was a LMTO of similar crystallinity to H_0.6_Li_0.08_Mn_1.72_O_4_, whereas the anode was a classic AC coated with the anion exchange membrane, here made of ethylene diamine‐modified PVC. After testing the electrochemical performances of the system with 2 m LiCl as electrolyte, the tests were further conducted in geothermal water from the Carpathian region (2.30 × 10^−3^ m, Poland), while using different configurations. The best selectivity was achieved when running the adsorption under constant voltage, the first desorption under zero charge voltage, and the second desorption under reversed voltage (see Figure 14b). Under these conditions, 73% of lithium recovery efficiency and a salt adsorption capacity of 131 mg_LiCl_ g^−1^ were reached. Furthermore, the energy consumption resulted to be approximately 8 Wh mol_Li_^−1^.

Whereas in the former cases the selectivity of the system was given by the lithium selective electrode, and the membrane aimed to improve the counter‐ions mobility, in the study by Shi et al. a cation exchange membrane was used to selectively separate lithium from magnesium in a MCDI device.^[^ ^283^ ^]^ Both electrodes were made of AC, carbon black and PVDF coated onto graphite paper and subsequently shielded by commercial ion‐exchange membranes. The use of a monovalent selective ion exchange membrane allowed to perform an efficient lithium recovery from a mixed solution of Li and Mg chlorides. Testing a mixed solution of 500 mg_Li_ L^−1^ (the concentration of cations was equimolar) for 10 min at 1.0 V and a flow rate of 30 mL min^−1^, the selectivity coefficient achieved was 2.95. Furthermore, this coefficient remained higher than one when increasing the Li/Mg ratio up to 60. Testing the setup in a large module, a removal rate of 38.4% was achieved for lithium and although its recovery rate decreased when increasing the initial concentration of lithium, the selectivity coefficient improved significantly. Furthermore, a smaller energy consumption than one of other technologies was recorded (≈1.8 Wh mol_Li_^−1^ for a large module in TDS of 500 mg L^−1^ and Mg/Li 1:1, vs ≈2.3 Wh mol_Li_^−1^for a small module), although higher than the previously reported MCDI processes (0.26 Wh g_salt_^−1 ^vs 0.18 Wh g_salt_^−1^ reported by Siekierka et al.^[^ ^37^ ^]^).

Ha et al. also used ion‐exchange membranes to coat both electrodes in a flow‐electrode‐based MCDI system.^[^ ^284^ ^]^ In this case, the electrodes were prepared as a slurry containing 20 wt% AC and 2.5 wt% of LiCl in DIW while they were continuously stirred throughout the whole operation (see Figure 14c). Commercial ion exchange membranes were also placed on the graphite current collectors, which included the electrode channels. Two different operational modes were tested, namely continuous and batch, using the same conditions. The desalination process was performed at a constant potential of 1.2 V. During adsorption, ions were adsorbed onto the electrodes and recombined in the tank while the feed solution passed into the nylon separator of the cell. A continuous recovery could be assured by changing the electrodes slurry when it is saturated. In this case, a larger size of the cell was found to improve the salt removal capacity. The highest efficiency was reached when using a 15 × 10^−3^ m feed solution, from which 91.7% of LiCl could be removed. The influence of the feed rate was also investigated, and the optimal conditions were found to be a low feed rate of around 3 mL min^−1^ coupled with high feed concentration. When operating in batch mode, instead, higher efficiencies could be reached by recirculating the feed solution several times.

In consonance with these strategies to enhance Li removal by electrochemical methods, Ryu et al. reported two attempts to modify the setup of MCDI. In this case, the adsorption was performed with ion sieves whereas the desorption did not use an acidic solution but an electrostatic field to lower the environmental impact of the process. In the study from 2013, the lithium selective adsorbent LiMn_2_O_4_ was coated on a current collector, whereas the other electrode consisted of a composite of PVDF and AC covered by an anion exchange membrane.^[^ ^228^ ^]^ The adsorption was conducted until equilibrium was reached then, after rinsing with DIW, a voltage was applied. The optimal voltage resulted to be 3.5 V at which 8.7 mg_Li_ g^−1^ were recovered from a 60 mg_Li_ L^−1^ LiOH solution. Although at that stage the method resulted to have an efficiency lower than the one of usual acid extractions, its main advantage was the absence of manganese ions, which normally were released. An upgrade of the setup was then reported in 2015.^[^ ^229^ ^]^ In this case, an electrostatic field was applied during both adsorption and desorption and the same pair of electrodes (LiMn_2_O_4_/PVA as the lithium selective electrode and AC as the capacitive electrode) were used separated by an anion exchange membrane were used. The adsorption was carried out at 1 V for 40 min to reach the electrode saturation, and the desorption was performed under the same conditions. The highest uptake was 1.36 mg_Li_ g^−1^ from an initial lithium concentration of 50 mg_Li_ L^−1^ at 1 V, proving an increased performance with respect to normal physisorption processes (ca. 0.5 mg_Li_ g^−1^).

More recently, in 2021, another MCDI cell was assembled using LMO as the positive electrode, and an AC electrode cover with an anion exchange membrane.^[^ ^292^ ^]^ Studying this device, Su et al. reported 51.8 mg_Li_ g^−1^ of lithium recovered when treating a brine with a high Na content (675 mg_Li+_ L^−1^ Na/Li ratio = 48.6), thanks to the use of carbon‐coated manganese oxide.

### 3.3 Electrodialysis

**Figure 15:**
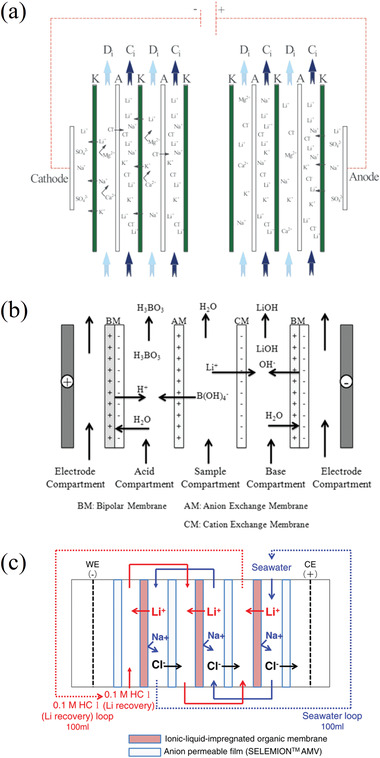
Schematic representation of electrochemical methods based on ED. a) Schematic principle of S‐ED, which anion exchange membranes (A), cation exchange membrane (K), desalting (D) and concentrating (C) compartments. Reproduced with permission.^[^ ^294^ ^]^ Copyright 2018, Elsevier. b) Schematic diagram of the BMED setup. Reproduced with permission.^[^ ^306^ ^]^ Copyright 2017, Elsevier. c) Schematic setup of the electrodialysis process with ionic‐liquid impregnated membrane. Reproduced with permission.^[^ ^301^ ^]^ Copyright 2013, Elsevier.

Electrodialysis, ED, is a membrane‐based technique in which ions of different size are separated under an applied electric field. Commonly used for desalination purposes, this electro‐driven technique can also be applied to enrich solutions of target ions when suitable membranes are used. Contrary to what happened in MCDI, in this case stacks of ion exchange membranes are arranged between the two electrodes by alternating cation‐exchange and anion‐exchange ones.^[^ ^233^ ^]^ That way, when feeding a solution into the cell, the ion transport will cause the formation of alternate compartments with concentrated and diluted solutions. In contrast to the CDI‐based approaches, the energy invested in the process cannot be recovered, thus the energy consumption is given by the thermodynamics associated with the lithium removal. ED architectures here explored include 1) monovalent selective membranes, 2) bipolar membranes and the 3) use of ionic liquids as additives in the membrane formulation (see **Figure** **15**a–c).

#### 3.3.1 Monovalent Selective Membranes

When using a monovalent selective ion exchange membrane, the process will be called selective‐ED (S‐ED, see Figure 15a) and divalent ions will be confined in the desalting compartment by means of either steric or electric repulsion.^[^ ^233^, ^294^, ^295^ ^]^ When S‐ED is aimed at recovering only one target ion such as lithium, further selectivity must be granted to the membrane. Although several studies mostly focus on the separation of lithium and magnesium, due to their similar radii in aqueous solutions, other monovalent ions such as sodium or potassium must also be considered. This has been attempted in different ways. Some authors report the use of chemically modified commercial membranes,^[^ ^296^, ^297^, ^298^, ^299^ ^]^ other use ionic liquids impregnated on a supporting membrane.^[^ ^300^, ^301^, ^302^ ^]^

As was previously discussed, a high ratio of magnesium over lithium ions (ranging between 1 and 65 and even 400 and 7600 in the case of the Mediterranean sea, see Table 2) is quite common in brines and constitutes one of the major problems to recover the monovalent ion from brine solutions. Several works aimed to address this issue. At first, most of the studies were made in binary mixtures before introducing other ions that would complicate the systems. This is the case of Nie et al. who investigated the efficiency of commercial monovalent selective ion exchange membranes in batch mode first in a binary solution and later in a multi‐ions one.^[^ ^298^ ^]^ By analyzing mixtures of 150 mg_Li_ L^−1^ and 10 to 60 g_Mg_ L^−1^, they observed that the best operative conditions were a mass ratio Mg:Li lower than 300, a low temperature (around 20 °C) and high flow rates to reduce the effect of diffusion. They reported a lithium recovery of ca. 90–95%. It was also concluded that in this kind of operation S‐ED was superior to nanofiltration membranes (NF) both in terms of efficiency and from an economical point of view. The process proved to be successful in recovering lithium in multi‐ions electrolytes, although showing a worse performance. The presence of potassium resulted to be particularly detrimental to the process. The same batch mode operation was used for further investigations.^[^ ^299^ ^]^ While under constant current the selectivity was improved but the energy consumption equally increased. The tests performed under constant voltage showed that better performances were obtained at higher voltages. This was most likely due to the decrease of the thickness of the EDL and therefore an increase in permselectivity toward lithium due to steric and electrical hindrance of magnesium ions. The system was then tested with a brine from the East Taijinar Lake (China) from which ca. 91% of lithium ions were successfully recovered with an energy consumption of 31 Wh mol_Li_^−1^.

The same correlation between permselectivity and voltage was also observed by Ji et al. who estimated 5 V as the optimal compromise between permselectivity and energy consumption.^[^ ^303^ ^]^ Under this condition, coupled with a Mg:Li ratio of 60, the authors reported an enrichment of a 0.148 g_Li_ L^−1^ solution to 0.210 g_Li_ L^−1^ after two hours of operation and a decrease of the Mg:Li ratio from 60 to 7. Conversely, operating at slightly higher voltages was deemed preferable when treating ternary mixtures because of the competitive migration of other ions, and they reported 76% of lithium recovered when prefractionating an East Taijinar brine at 10 V while consuming 660 Wh mol_Li_^−1^.^[^ ^297^, ^304^ ^]^ The effect of co‐existing ions was also investigated and it was found that among monovalent cations the presence of potassium was more detrimental than the one of sodium, whereas the presence of calcium could induce water splitting because of intense concentration polarization. Overall, the influence order of coexisting cations was found to be contrary to their hydrated radius sequence, underlining the effect of steric hindrance, similarly to what happens in NF. As far as counter‐ions were concerned, instead, the presence of sulfate ions could improve the lithium‐ion transport thus resulting beneficial to its recovery.^[^ ^294^, ^304^ ^]^

#### 3.3.2 Bipolar Membranes

Bipolar membranes (BP) have also been taken into consideration in the ED configuration. BP membranes are layered ion exchange membranes composed by two polymers carrying opposite charges. This way, no ions can move from one side of the membrane to the other one, but the disproportionation reaction of water would take place at the hydrophilic junction when a suitable electric potential is applied.^[^ ^305^ ^]^ The presence of a bipolar membrane in the ED setup entails the formation of hydroxyl groups and protons in the different compartments. Bipolar membranes have been mostly used to separate both lithium and boron ions from the same stream, seeing how both elements are of high importance in different industrial fields. In this case, lithium would be recovered as LiOH. Boron, as borate ion in seawater, would be recovered as boric acid. Accordingly, the feed solution must be predominantly alkaline, while divalent cations such as magnesium and calcium need to be removed previously to this process, as they easily form borate complexes.^[^ ^306^, ^307^, ^308^, ^309^ ^]^

The experimental setup proposed by Bunani et al. presented both conventional ion‐exchange membranes and BP membranes (Figure 15b).^[^ ^306^ ^]^ For this specific kind of operation, the feed solution was neither a natural nor a synthetic brine but Li_2_B_4_O_7_*5H_2_O (ca. 850 mg_B_ L^−1^ and 250 mg_Li_ L^−1^), and the acid and base solution 3 × 10^−3^ m HCl and NaOH respectively. By testing different voltages, a correlation between the applied electrical potential and the recovery efficiencies of both elements was found, although the recovery rates of lithium overcame the ones of boron. The effect of the sample volume and pH were also investigated, revealing that boron recovery was more influenced by these parameters than lithium. Further studies were conducted by varying the concentration of both elements in the feed solution and by adding sodium chloride to investigate the influence of co‐existing ions.^[^ ^307^ ^]^ Although the presence of sodium did not affect the transport of lithium ions into the base compartment, sodium was transported to the same compartment as well. When investigating the effect of the concentration of the acid and base solutions, higher concentrations were found to lead to increased ion transport in reason of higher conductivities.^[^ ^308^ ^]^ This of course entailed that, to achieve the same result, higher concentrations were required when choosing weaker acids/bases than with strong electrolytes. Results also showed the possibility of ion retention by the membranes causing a decrease of the recovery. Solutions to this problem could be longer runs or a different choice of the membranes used. The overall optimal operational conditions were found to be 50 × 10^−3^ m HCl and NaOH and an applied electrical field of 30 V that resulted in 50% of boron and 62% of lithium recovery with a specific power consumption of 7.9 kWh m^−3^.^[^ ^308^ ^]^ A more recent work by Jarma et al.^[^ ^309^ ^]^ reported higher recovery values of both boron (ca. 57%) and lithium (ca. 89%) at lower electrical potentials (20 V) and with the same solutions (50 × 10^−3^ m HCl and NaOH).

#### 3.3.3 Ionic Liquids in Electrodialysis

The possible lithium recovery by using ionic liquids, either in liquid–liquid extraction or in supported liquid membranes, has already been highlighted (see Section 2.5.2). Like other passive processes here discussed, the setup can be upgraded by applying an electrostatic field. It is the case of Hoshino, who in 2013 reported the use of a Gore‐Tex membrane impregnated with the ionic liquid [PP13][TFSI] (N‐methyl‐N‐propylpiperidium bis(trifluoromethylsulfonyl)imide) and covered it with a Nafion 324 overcoat.^[^ ^301^ ^]^ The membrane thus prepared was tested in an electrodialysis setup coupled with a commercial anion exchange membrane to selectively recover lithium from seawater (see Figure 15c). After running the process for 2 hours at 2 V, the concentration of lithium in the recovery solution resulted to be 37.7 mg_Li_ L^−1^ corresponding to a recovery ratio of 22%. A further step of 1 h at 18 V was then required to separate lithium ions from the chloride ones and showed an efficiency of 95%. A similar setup was further developed using a lithium‐ion conductive glass‐ceramic as the separation membrane. In that case, the cell worked like an ion concentration cell without the application of an external electric potential and in which the membrane had the role of salt bridge.^[^ ^310^ ^]^ There have been more records of novel membranes formulated with ionic liquids. As an example, using a conductive glass ceramic combined with diazo[2,2,2]bicyclooctane (DABCO)‐grafted polyepichlorohydrin membrane was proposed lately.^[^ ^311^ ^]^

More recently, a liquid membrane of RTIL was instead used by Liu et al. to recover lithium from brines with a high Mg:Li ratio.^[^ ^302^ ^]^ In this case, the liquid membrane of [C4mim][TFSI] (1‐butyl‐3‐methyl‐imidazolium bis(trifluoro‐methyl‐sulfonyl)imide) was sandwiched between two solid cation exchange membranes to contain it. The system presented not only the highest lithium migration rate among the RTIL systems studied, but also a higher current efficiency, 65%, and lower energy consumption, 16 Wh g^−1^ Li, than ordinary electrodialysis processes. After 12 h of operation the Mg:Li ratio dropped from 53 to 0.26 when treating a West Taijinar brine (2.21 g_Li_ L^−1^, 118 g_Mg_ L^−1^) also blocking other ions, thus proving to be a very efficient method to recover lithium.

An opposite approach from one of Liu was reported by Hoshino.^[^ ^300^ ^]^ In their study to recover lithium from seawater for tritium fuel production, a RTIL liquid membrane was used sandwiched between ion‐exchange membrane to process concentrated seawater, free of divalent ions. The particularity of the study resided in the choice of the RTIL, N,N,N‐trimethyl‐N‐propylammonium‐bis(trifluoromethanesulfonyl)imide (TMPA‐TFSI) which presented low lithium conductivity. During the 15 h process at 2–3 V, lithium ions were therefore confined in the anode compartment whereas other monovalent ions were transported into the cathode one filled with 100 × 10^−3^ m HCl. The recovery of lithium proved to be more efficient when magnesium was added to the feed solution previously containing only monovalent ions. This way, 63% of lithium ions could be retained in the anode compartment instead of only 38%.

**Figure 16:**
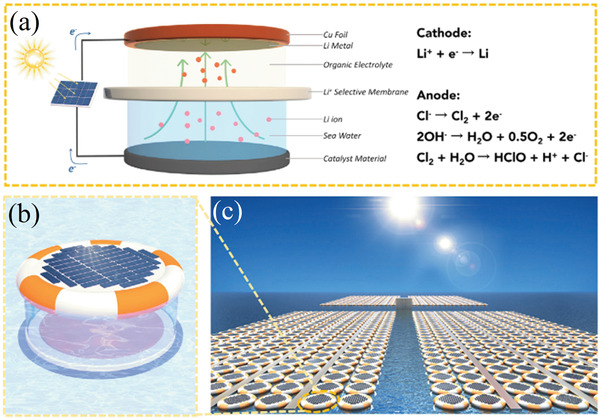
Schematic of the solar‐powered device. a) Working mechanism, b) single unit device, c) scale‐up of the device. Reproduced with permission.^[^ ^293^ ^]^ Copyright 2016, Elsevier.

Finally, we present a prototype of a device realized by Yang et al. able to recover metallic lithium from seawater.^[^ ^293^ ^]^ This electrolytic cell was built from two half‐cells separated by a NASICON‐type solid‐state electrolyte as a lithium ion selective membrane (**Figure** **16**a–c). The catholite contained an organic electrolyte (LiClO_4_‐propylene carbonate solution) whereas the anolite contained seawater. The cell was charged by a constant current from the solar panel during the electrolytic process, where lithium was able to pass from the anode compartment to the cathode one. There, it was reduced to its metallic form on the copper foil. After one hour the cell with 1 cm^2^ copper foil as lithium collector presented a production rate of 5.7 mg dm^−2^ h^−1^. Furthermore, they claimed that their design would be easy to scale‐up by floatable arrays in seawater.

## 4 Limitations and Challenges

A direct comparison of the different processes cannot be achieved without taking into account the impact of different key factors in the experimental practice on the reported results. In order to address this challenge, several figures of merit are here proposed.

**Figure 17:**
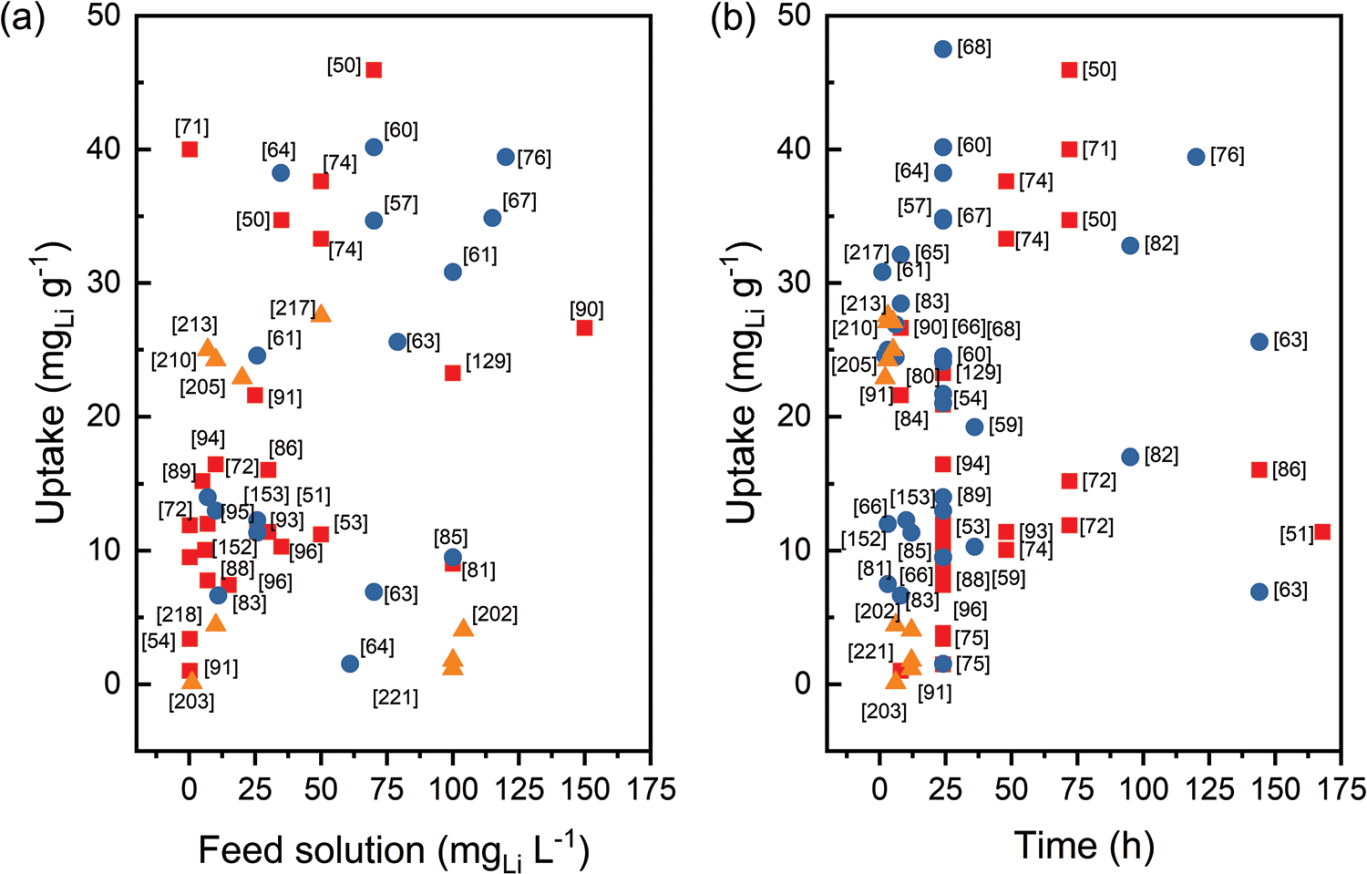
Uptake versus a) feed solution concentration and b) time of adsorption of the adsorbents analyzed. LMO adsorbents are represented by red squares, LTO by blue circles and crown ethers by orange triangles.

In the case of passive adsorption processes, the uptake of each adsorbent with respect to the concentration feed and the time to reach the thermodynamical equilibrium were chosen. **Figure** **17** shows an overview of these two figures of merit for the materials reported here. However, since the concentration of the feed solution presents an incredibly high dispersity in this field, an executive choice was here made and only realistic feeds with a concentration lower than 175 mg_Li_ L^−1^, i.e., 25 × 10^−3^ m, were taken into consideration. A table detailing all the different experimental conditions of the reported literature can be found in Table S1 (Supporting Information).

A quick glance at the data will suffice to see that adsorbents tested in solutions with higher initial concentrations can easily provide better results in terms of uptake. However, this does not mean that higher concentrations automatically imply higher uptakes nor that those can be scaled up or down in a linear way. This is particularly evident from studies that report the results of lithium uptake under different concentrations of the feed solution.^[^ ^50^, ^57^, ^64^, ^82^, ^86^ ^]^

**Figure 18:**
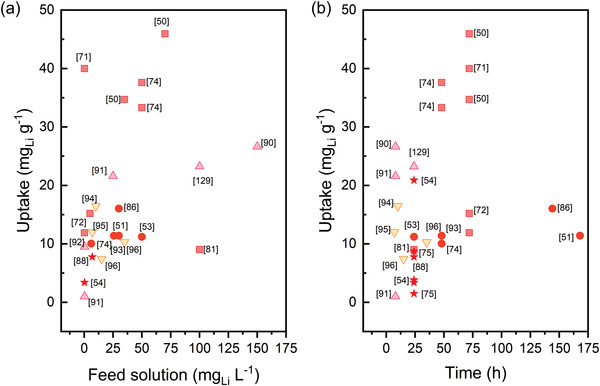
Uptakes of the LMO ion sieves versus a) the concentration of the feed solution and b) the time of adsorption. Red squares represent powder morphologies, red circles granulates, pink triangles membranes, red stars foams and yellow reverse triangles NF adsorbents.

**Figure 19:**
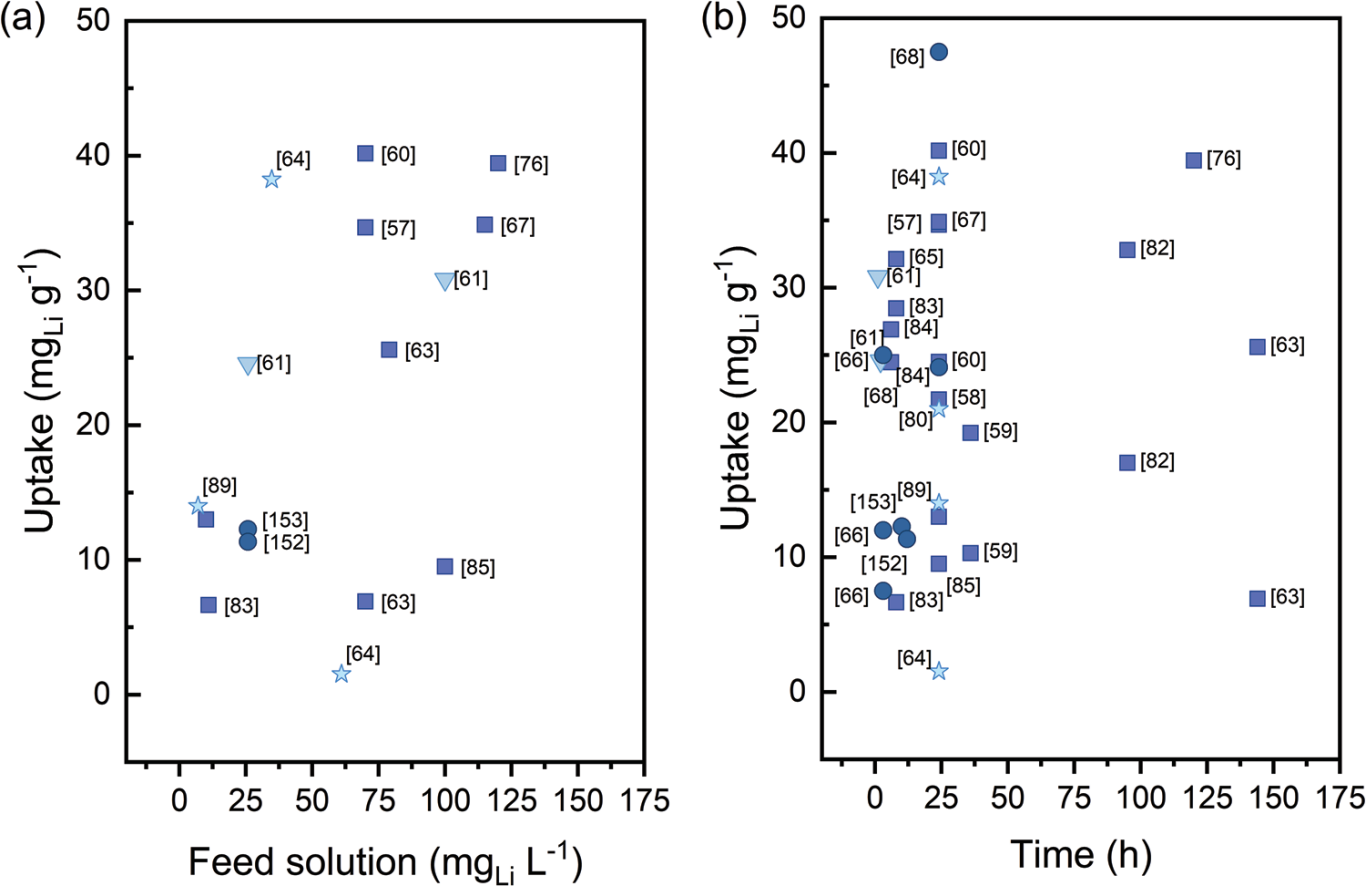
Uptakes of the LTO ion sieves versus a) the concentration of the feed solution and (b) the time of adsorption. Blue squares represent powder morphologies, blue circles granulates, blue stars foams and reverse triangles fibers.

The best adsorbents would need to be in the upper left area of both graphs of Figure 17. The adsorbents that fall in that area, in fact, would provide high uptakes at low feed concentrations in short times. By comparing different kind of adsorbents, one can see that the most suitable class of adsorbents would then be the LMO ion sieves and, successively, the LTO ion sieves. An inset of the different morphologies of LMO ion sieves can be found in **Figure** **18**, whereas those of LTO ion sieves are shown in **Figure** **19**.

As far as materials are concerned the best choice highly depends on the characteristics of the feed solution. The same adsorbent will not work equally well in seawater or in a brine, in an alkaline solution, or in an acidic one.^[^ ^75^, ^84^ ^]^ Depending on the acidity of the solution, for example, LMO ion sieves, which are generally considered to be the most performant, should be replaced with LTO ion sieves, which are more stable.^[^ ^88^, ^89^ ^]^ When taking into account the morphology, it is apparent that, as was stated in the previous paragraphs, the most performant adsorbents are still represented by powder materials. However, LMO‐loaded membranes and LTO‐loaded foams can be valid candidates for scalable technologies. To date, the scaling down of the adsorbents and the use of hybrid supported nanostructured materials appears to be the best option.

Although passive processes like adsorption can show high selectivity and good lithium recovery, they also need chemical treatments to ultimately recover the target ions from their structure. This postprocessing can be challenging from an environmental point of view, as strong acids are generally involved. Additionally, adsorption processes generally may need more time and a pre‐treatment of the brine to increase the efficiency of the recovery.

The high variety of experimental conditions highlights the need for standards when operating in this field. The main aspects that need to be regularized are the composition of the feed solution and time of adsorption, and the regeneration solution, both in terms of composition and concentration. The variability of the first two parameters can be partly assessed when looking at Figure 17, although the cases studied presented feed solutions with up to 7 g_Li_ L^−1^, i.e., 1000 × 10^−3^ m, which seems unrealistic for actual recovery trials. Regarding the regeneration solution, while the most used is generally HCl, its composition usually varies between 100 × 10^−3^ m and 1000 × 10^−3^ m and other acids are also used. Further aspects that should be considered are the number of cycles in the durability tests as depending on the works they can vary from just a couple to ten or more, and the presence or absence of stirring in batch experiments. Also, although the study of the active material is of capital importance, more attention should be paid to a possible scalability and automation of the setup. While batch adsorption is good for preliminary studies, it would not be practical with higher volumes of solution.

Active materials and configurations to recover lithium by means of electrochemical processes, as well as evaluation parameters, have been principally adapted from the battery research field. The Coulombic efficiency, used to indicate the amount of lithium that can be intercalated in the electrode and the reversibility of the process, is an example of the shared ground between these two fields.^[^ ^231^ ^]^ When reviewing the materials of lithium intercalation electrodes used in electrochemical processes, LMO electrodes have proven to be more stable than LFP ones. Moreover, depending on the brine composition (i.e., ratio between the Li vs different cations), the choice between LFP or LMO could have a great impact on the lithium recovery. Here, LFP shows a better performance regarding the lithium selectivity in those brines with higher content of Na^+^ or K^+^. In the case of LMO, the higher selectivity of lithium toward Mg^2+^ allows the use of this type of active material in the extraction of Li from brines such as Salar Uyuni (see Table 2).^[^ ^255^ ^]^ Finally, regarding the type of counter electrodes, the best candidates seem to be lithium exclusion electrodes such as NiHCF due to their low cost, high stability, and superior performances in terms of purity and efficiency of lithium removal.^[^ ^255^ ^]^

**Figure 20:**
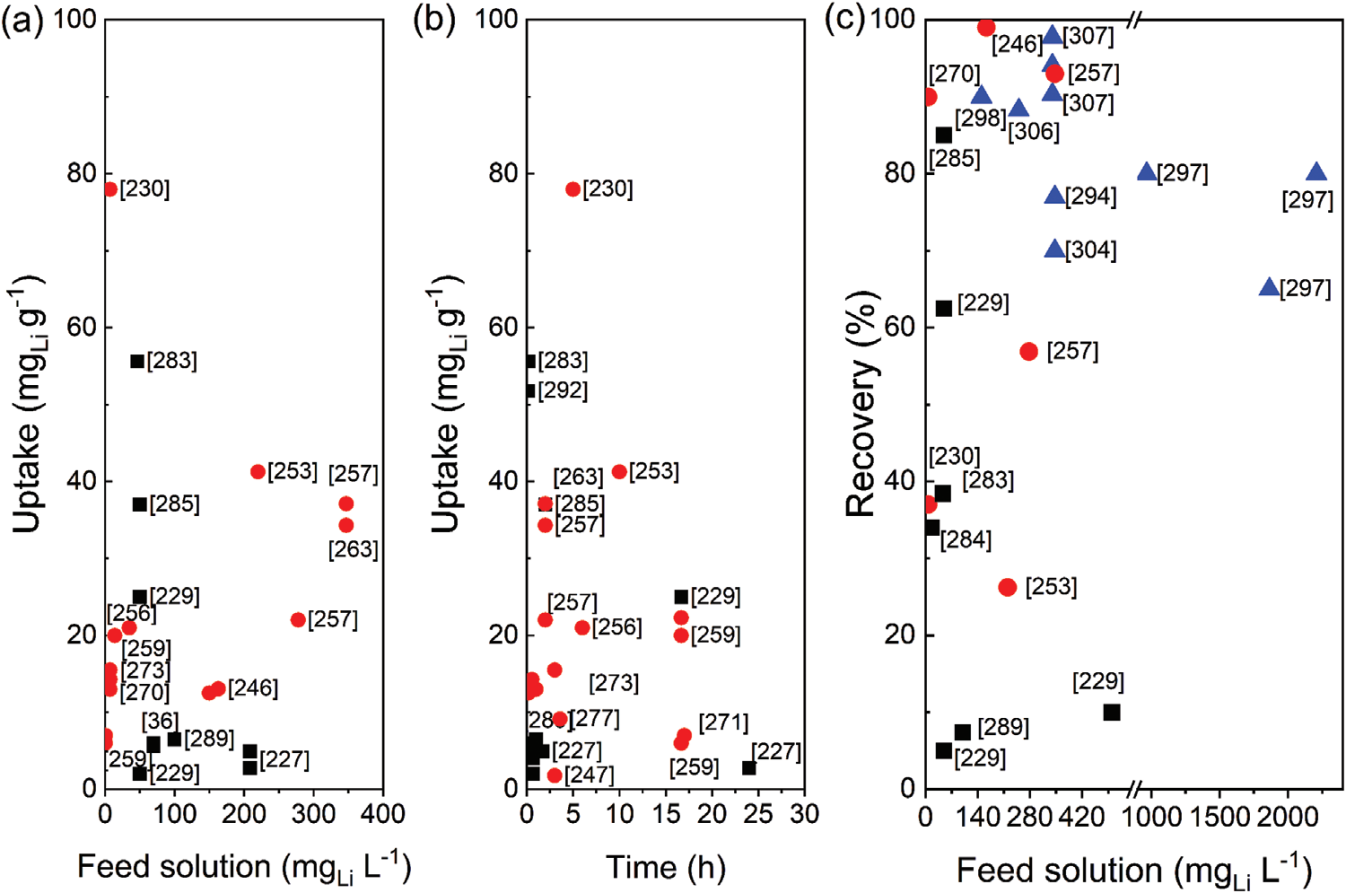
Uptakes of the ion‐pumping and MHCDI technologies versus a) the concentration of the feed solution and b) the time of electroadsorption. c) Recovery versus concentration of the feed solution. MHCDI technologies are represented by black squares, ion‐pumping by red circles and ED‐based by blue triangles.

One of the main challenges that rise when comparing the emerging electrochemical lithium recovery technologies is the lack of a standardized protocol and set of parameters to refer to. This is probably because of the relatively recent development of this sector and the large variety of alternative untapped lithium sources.^[^ ^231^ ^]^ As in the case of passive processes (see Figure 17), two figures of merit are here proposed, namely the uptake of lithium versus its concentration in the feed solution and versus the time over which lithium is removed. Additionally, the recovery as a function of the feed concentration has also been plotted to compare these electrochemical methods (**Figure** **20**).

In general, the lithium uptake is higher than 25 mg_Li_ g^−1^ (Figure 20a). However, CDI‐based technologies show the higher the feed solution concentration, the less lithium is taken up. Most importantly, Figure 20b shows that these considerably high values of lithium uptake can be achieved in operational modes that do not require long lithium removal time periods in comparison to passive processes. Thus, electrochemical processes may increase the lithium recovery rate, reaching on average 170 mg_Li_ g^−1^ d^−1^, versus ≈10 mg_Li_ g^−1^ d^−1^ achieved in passive processes.

Additionally, based on literature, Figure 20c depicts recovery data to compare different electrochemical methods. While the lithium recovery for ED‐based studies is higher than 75% and seems to be independent of the feed solution, a lithium recovery ranging from 70% to 98% has been reported for approximately the same feed concentration. This variation in the results might be explained by the dependence of the recovery on the operational conditions, being the cell voltage the one with more impact on the lithium uptake by using ED‐based methods (see Table S2 in the Supporting Information). In contrast, in MHCDI and ion‐pumping technologies, the recovery tends to decrease upon the lithium concentration in the feed. Nevertheless, compared to ED approaches, higher selectivity results have been reported for ion‐pumping studies (see Table S2 in the Supporting Information). In terms of technology comparisons, we encourage authors to provide an estimation of the lithium production (g_Li_ h^−1^ m^−2^) to allow a more complete evaluation among electrochemical methods.

**Figure 21:**
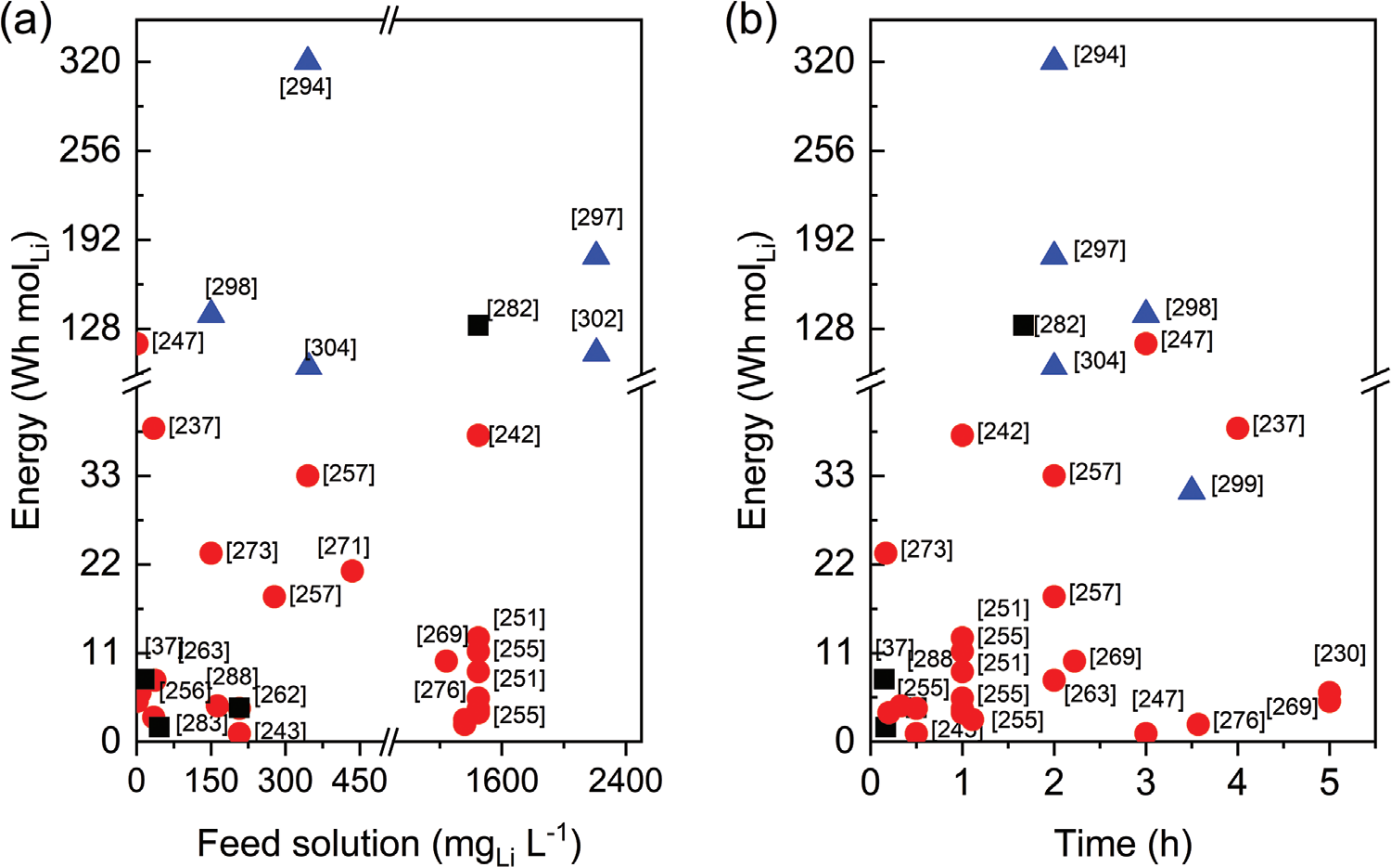
Electrochemical‐based lithium recovery technologies comparison in terms of energy consumption versus a) concentration of the feed solution and b) versus the time of electroadsorption. MHCDI technologies are represented by black squares, ion‐pumping by red circles and ED‐based by blue triangles.

Overall, the former comparisons highlight how the lithium removal performance of the electrochemical methods depends on the operational modes, and therefore affects the net energy consumption of the whole process. From an energetic point of view (see **Figure** **21**), capacitive deionization processes seem to be the most effective methods, but often show lower capacity values of recovered lithium in comparison to ion pumping techniques. Among these electrochemical methods, it is worth mentioning that ED‐based technologies seem to be the most unattractive in terms of net energy consumption. The removal of lithium ions by means of ion‐pumping methods provides a more stable operation and higher specific capturing rates covering a wider range of lithium concentrations in the feed solution. In addition, having no need for membranes, high operating pressures or thermal energy inputs, ion‐pumping strategies are superior to the other electrochemical technologies, such as MHCDI or ED, or the passive processes, in terms of operation as well as maintenance costs.

It should also be noted that, despite reporting the energy consumption, the amount of lithium recovered, or the selectivity of the process is quite common, rarely all of them are given in the same study (see Table S2 in the Supporting Information). The same applies to operational protocols, the characteristics and nature of the electrode and the reactor design, which can have major influences on the recovery of lithium. To facilitate future objective evaluations of different lithium removal scenarios, we encourage to clearly report essential data such as mass loading, the total mass of the active material and the electrode's surface area as well as the number of membranes used and their dimensions (when applicable). In addition, the number of electrodes, the separator thickness, and the operational conditions such as flow rate and pump energy consumption will help to evaluate different techniques in a better way.

Accordingly and independently from the electrochemical technology studied, we propose not only to use a standard feed solution (i.e., Atacama brine), but also to report the following metrics: amount of lithium removed (to facilitate the estimation of the lithium recovery and the capacity of the electrodes), duration of the experiment (including the duration of the single steps, i.e., lithium removal, release, and washing) selectivity, purity of the final solution produced and the net energy consumption (i.e., Wh mol_Li_^−1^ and Wh g_Li2CO3_^−1^).

## 5 Conclusions and Future Perspectives

The ever‐increasing importance of lithium‐ion batteries in today's economy, coupled with the use of lithium in several other fields such as glass and ceramics production and aluminum refinery process, makes lithium supply a topic of capital interest in the subject of global mineral commodities availability. After having reviewed part of the available resources and taking into consideration the ongoing depletion of solid lithium resources, it is reasonable to focus on water resources, which account for two‐thirds of its global availability. Currently, the global production of lithium from brines is by lime‐soda evaporation, which is intensely time and water consuming. In contrast, the alternative production methods/approaches herein considered are all based on the mobility of a minority phase (i.e., lithium ions) in a majority phase (i.e., the water resource).

In this context, we decided to focus our study on reviewing first passive approaches (e.g., evaporation or adsorption) and then setups that have been implemented by adding an external electric field. This approach was chosen because many materials that have been first studied and developed as pure adsorbents have later on been used as electrode materials (see the paragraphs on LMO‐type LIS and the electrodes of battery‐based ion pumping techniques).

The preparation method of the adsorbents/electrodes plays a crucial role in its lithium recovery capacity (see **Table** **4**). The composition and preparation of this kind of materials are generally meticulously described, as different preparation modes entail different superficial defects and impurities which may have a significant impact when the material is working in passive processes and in electrodes working under an electrical field.

**Table 4:** Summary of the LIS studied

Type of LIS	Stoichiometry	Structure studied in	Synthesis method	Sub‐method	Refs.
LMO	*λ*‐MnO_2_ (LiMn_2_O_4_ in batteries)	^[^ ^119^, ^121^ ^]^	Solid state	Calcination	^[^ ^52^, ^87^, ^124^, ^227^, ^228^, ^229^, ^238^, ^239^, ^257^, ^258^, ^282^ ^]^
				Microwave combustion	^[^ ^48^ ^]^
			Soft chemical	Hydrothermal	^[^ ^71^ ^]^
				Sol‐gel	^[^ ^75^ ^]^
	*β*‐MnO_2_ (Li_4_Mn_5_O_12_)		Solid state	Calcination	^[^ ^55^ ^]^
			Soft chemical	Hydrothermal	^[^ ^50^, ^53^, ^129^ ^]^
	H_1,6_Mn_1,6_O_4_		Solid state	Calcination	^[^ ^88^, ^94^ ^]^
			Soft chemical	Hydrothermal	^[^ ^90^, ^95^, ^96^ ^]^
				Sol‐gel	^[^ ^74^ ^]^
	H_1,33_Mn_1,67_O_4_		Solid state	Calcination	^[^ ^51^, ^54^, ^56^, ^81^, ^86^, ^91^ ^]^
			Soft chemical	Hydrothermal	^[^ ^73^ ^]^
LTO	H_2_TiO_3_ layered	^[^ ^121^, ^146^ ^]^	Solid state	Calcination	^[^ ^57^, ^58^, ^59^, ^60^, ^62^, ^63^, ^65^, ^66^, ^67^, ^69^, ^77^, ^84^, ^89^ ^]^
			Soft chemical	Hydrothermal	^[^ ^82^ ^]^
				Sol‐gel	^[^ ^80^, ^151^ ^]^
	H_4_Ti_5_O_12_ spinel		Soft chemical	Hydrothermal	^[^ ^64^, ^76^, ^78^, ^79^, ^83^ ^]^
	LiTiO_2_	^[^ ^141^, ^144^ ^]^			
	LiTi_2_O4	^[^ ^142^, ^145^ ^]^	Solid state	Calcination	^[^ ^85^ ^]^
LMTO			Solid state	Calcination	^[^ ^36^, ^37^, ^70^ ^]^
LFP	LiFePO_4_	^[^ ^121^ ^]^	Solid state		^[^ ^248^ ^]^
			Solid liquid		^[^ ^253^, ^254^ ^]^

One of the topics that still needs considerable research instead is the manufacturing process of the active material. In fact, in the case of pure adsorption processes, powder materials often provide the highest efficiencies, but they are of difficult handling. The need for suitable technologies that can either granulate of produce membranes and foams while not decreasing their adsorption properties is therefore a crucial point. Lastly, a possible integration of nanostructured materials, which have proven to be the most performing electrodes, in more complete setups would also be advantageous. This integration could either come in the form of pretreated feed solutions, thus removing problematic elements like magnesium, and reducing the amount of strong acids used as eluent, or in the case of electrochemical processes the use of energy from renewable sources to power the reactor, thus lowering the fingerprint of the whole process.

So far, no standard protocol to measure the selectivity of a process nor its recovery efficiency has been adopted. Several equations have been used for the former, but they depend on the kind of solutions used for both feed and recovery, which can greatly differ from one study to another and have a high influence on the final recovery rates. This difference is encountered both in composition and concentration and whereas pure adsorption studies often use HCl as eluent, although in quite a wide range of concentration, electrical field‐enhanced setups limit the use of strong acids. In order to go in the direction of a more systematized research environment, Battistel et al. proposed to use a solution reflecting the composition of the Atacama brine as the standard feed solution, and the washing of the setup with deionized water between the use of the different solutions in order to avoid contaminations.^[^ ^231^ ^]^ Complementary, we propose to clearly define and report in detail parameters such as the amount of active material, cell/device characteristics, and operational conditions.

In summary, the main challenge encountered in attempting to perform this comparative study was the inhomogeneity of parameters used in the different fields. Parameters such as removal capacity, energy consumption, selectivity, duration of the experiment and durability tests are usually reported both in passive and electrochemical processes. Nevertheless, several important features such as the temperature and the presence of a static bath or stirring in the case of adsorption processes are often overlooked. The lithium recovery performance of adsorbents/electrodes is also often used as key parameter but does not account for the selectivity of the material. We therefore encourage to report at least the following basic performance metrics and indicators, independently from the nature of the process examined: 1) external conditions of the experiment (at least temperature and experiment duration, possibly static/stirring); 2) stoichiometry and synthesis of the active material (if not commercially available); 3) cell and setup characteristics (total amount of active material used, projected area of membranes and/or electrodes); 4) composition of both feed and regeneration solution, and of the washing solution, the use of which is strongly recommended; 5) performance metrics: amount of lithium recovered (capacity, lithium removal rate and % of lithium removed, or lithium concentration reduction, from the feed solution as well as the concentration increase in the recovery solution), selectivity and purity as well as energy consumption (when applicable).

Here we note the importance of reporting the purity of the lithium recovered solution produced. With a future industrial application in view to produce Li_2_CO_3_, the final product demanded by the marked, the lithium enriched solution produced (LiCl) should present the highest purity (at least 99%) to be easily converted in Li_2_CO_3_ through precipitation with Na_2_CO_3_.

Considering a short‐term scenario with a possible scale‐up of the processes, an extensive optimization of the design of the reactors would be needed, both for pure adsorption and for electrically‐enhanced processes. For the former, extensive studies that do not only focus on the material but also on a scalable and automated setup, like a cell that would allow to flush the different solutions without having to manually remove the adsorbents would be necessary. The same would be needed in the case of electrically enhanced reactors, for which designs with only one cell instead of two would be beneficial and the flushing of a washing solution between the processes could limit the influence of the dead volume.

## Conflict of Interest

The authors declare no conflict of interest.

## Supporting information

Supporting Information
