# Supplementary material for: Recent Advances in the Lithium Recovery from Water Resources: From Passive to Electrochemical Methods
Source: Adv Sci (Weinh). 2022 Jul 27;9(27):2201380. doi: 10.1002/advs.202201380 (PMC9507372; doi:10.1002/advs.202201380)
Supplement: Supplementary file 1 — Supporting Information [file ADVS-9-2201380-s001.pdf]

Supporting Information

Recent advances in the lithium recovery from water resources: from passive to electrochemical methods

Luisa Baudino[a,b,]*, Cleis Santos[c,]*, Candido F. Pirri[a,b], Fabio La Mantia [c,⁑] and Andrea Lamberti[a,b ⁑]

[a] *DISAT Dipartimento di Scienza Applicata e Tecnologia, Politecnico di Torino, corso Duca degli Abruzzi 24, 10129, Torino, Italy*

[b] *Istituto Italiano di Tecnologia, Center for Sustainable Future Technologies, Via Livorno 60, 10144, Torino, Italy*

[c] *Universität Bremen, Energiespeicher- und Energiewandlersysteme, Bibliothekstraße 1, 28359 Bremen, Germany*

**These authors contributed equally to this work.*

⁑Corresponding author. E-mail addresses: lamantia@uni-bremen.de; andrea.lamberti@polito.it

Table S1. Summary table of the uptake from the passive methodologies and cases studied. N.B. The feed solution and uptake quantities are $mg_{Li}\ g_{adsorbent}^{-1}$ or $mg_{Li}\ L^{-1}$ of solution.

TYPE	METHODOLOGY	FEED SOLUTION	EXPERIMENTAL CONDITIONS	UPTAKE	REGENERATION SOLUTION	CYCLES	REF
**PVA/GO+12C4E NF membrane**	Supramolecular system	1000 $mg_{Li}\ L^{-1}$	24 h at RT	97.23 $mg_{Li}\ g^{-1}$	0.5 M HCl	5	[1]
**HZrTO powder**	Ion sieve	1800 $mg_{Li}\ L^{-1}$ LiOH<br>Qaidam brine (1560 $mg_{Li}\ L^{-1}$)	24 h at RT	93.20 $mg_{Li}\ g^{-1}$<br>51.80 $mg_{Li}\ g^{-1}$	0.25 M HCl	3	[2]
**Li_2CO_3+TiO_2 thermally treated powder**	Ion sieve	7000 $mg_{Li}\ L^{-1}$ LiOH<br>70 $mg_{Li}\ L^{-1}$<br>10 $mg_{Li}\ L^{-1}$	175 rpm, 24 h at 303K	94.50 $mg_{Li}\ g^{-1}$<br>34.67 $mg_{Li}\ g^{-1}$<br>13.00 $mg_{Li}\ g^{-1}$	0.2 M HCl	5	[3]
**Granulated LMO/19.4 wt% chitosan**	Ion sieve	360 $mg_{Li}\ L^{-1}$ LiCl<br>30 $mg_{Li}\ L^{-1}$ LiCl	0.9 L min^{-1}, 6 days at RT	62.94 $mg_{Li}\ g^{-1}$ 16.04 $mg_{Li}\ g^{-1}$	0.2 M H_2SO_4	3	[4]
**Calix[4]arene Li-IIM**	Supramolecular system	200 $mg_{Li}\ L^{-1}$	in incubator 3 h at RT	50.87 $mg_{Li}\ g^{-1}$	0.2 M EDTA	4	[5]
**HZrTO granulated with epoxy resin**	Ion sieve	1800 $mg_{Li}\ L^{-1}$ LiOH<br>Qaidam brine (1560 $mg_{Li}\ L^{-1}$)	24 h at RT	47.50 $mg_{Li}\ g^{-1}$<br>24.1 $mg_{Li}\ g^{-1}$	0.25 M HCl	3	[2]
**Nanorods β-MnO_2**	Ion sieve	69.4 $mg_{Li}\ L^{-1}$<br>34.7 $mg_{Li}\ L^{-1}$	150 rpm, 72 h at 303K	45.94 $mg_{Li}\ g^{-1}$<br>34.70 $mg_{Li}\ g^{-1}$	0.1 M HCl	N.A.	[6]
**H_2TiO_3 powder**	Ion sieve	70 $mg_{Li}\ L^{-1}$<br>West Taijinar brine (669 $mg_{Li}\ L^{-1}$)	24 h at 303 K	40.16 $mg_{Li}\ g^{-1}$<br>24.5 $mg_{Li}\ g^{-1}$	0.18 M HCl	5	[7]
**$H_{1.6}Mn_{1.6}O_4$ powder**	Ion sieve	Seawater	3 days	40.00 $mg_{Li}\ g^{-1}$	0.5 M HCl	2	[8]
**TiO_2 nanotubes**	Ion sieve	120 $mg_{Li}\ L^{-1}$ LiCl	120 h at RT	39.43 $mg_{Li}\ g^{-1}$	0.5 M HCl	N.A.	[9]
**Anatase Li_2TiO_3 powder**	Ion sieve	2000 $mg_{Li}\ L^{-1}$ LiOH	N.A.	39.20 $mg_{Li}\ g^{-1}$	0.25 M HCl	N.A.	[10]

TYPE	METHODOLOGY	FEED SOLUTION	EXPERIMENTAL CONDITIONS	UPTAKE	REGENERATION SOLUTION	CYCLES	REF
**Macroporous/mesoporous 3DM - $Li_4Ti_5O_{12}$**	Ion sieve	34.7 mg_{Li} L^{-1} LiOH<br>61 mg_{Li} L^{-1}	24 h at RT	38.24 mg_{Li} g^{-1}<br>1.53 mg_{Li} g^{-1}	0.1 M HCl	6	[11]
**LTO from sludge**	Ion sieve	115 mg_{Li} L^{-1} LiOH	24 h at RT	34.88 mg_{Li} g^{-1}	0.2 M HCl	4	[12]
**$H_{1.36}Li_{0.07}Mn_{1.65}O_4$<br>$H_{1.41}Li_{0.01}Mn_{1.65}O_4$**	Ion sieve	50 mg_{Li} L^{-1} LiCl<br>(pH 12)	48 h at RT	33.30 mg_{Li} g^{-1}<br>37.60 mg_{Li} g^{-1}	0.5 M HCl	N.A.	[13]
**Powder H_xTiO_3**	Ion sieve	1560 mg_{Li} L^{-1}<br>200 mg_{Li} L^{-1}	300 rpm, 95 h at 303K	32.80 mg_{Li} g^{-1}<br>17.00 mg_{Li} g^{-1}	0.5 M HCl	6	[14]
**Al-H_2TiO_3**	Ion sieve	252 mg_{Li} L^{-1}	8 h at RT	32.12 mg_{Li} g^{-1}	0.2 M HCl	5	[15]
**H_2TiO_3 + PSf fibers**	ion sieve	100 mg_{Li} L^{-1} LiCl<br>geothermal brine 25.78 mg_{Li} L^{-1}	1 h at RT<br>2 h at RT	30.83 mg_{Li} g^{-1}<br>24.57 mg_{Li} g^{-1}	0.2 M HCl	5	[16]
**H_2TiO_3 from amorphous TiO_2**	Ion sieve	300 mg_{Li} L^{-1}	24 h at RT	30.03 mg_{Li} g^{-1}	0.6 M $K_2S_2O_8$	5	[17]
**Yolk-shell structured C@$Li_4Ti_5O_{12}$ microspheres**	Ion sieve	347 mg_{Li} L^{-1} LiOH<br>11.13 mg_{Li} L^{-1}	8 h at RT	28.46 mg_{Li} g^{-1}<br>6.65 mg_{Li} g^{-1}	0.05 M HCl	N.A.	[18]
**Li-imprinted membranes (Li-IIMs)**	Supramolecular system	50 mg_{Li} L^{-1}	3 h at RT	27.55 mg_{Li} g^{-1}	1 M HCl	5	[19]
**PVDF/GO+pDA+12C4E membrane**	Supramolecular system	200 mg_{Li} L^{-1} LiCl	5 h at 298K	27.10 mg_{Li} g^{-1}	1 M HCl	6	[20]
**PVDF+pDA+12C4E membrane**	Supramolecular system	200 mg_{Li} L^{-1} LiCl	3 h at 298K	27.10 mg_{Li} g^{-1}	0.5 M HCl	6	[21]
**Li_2CO_3+TiO_2 thermally treated powder**	Ion sieve	694 mg_{Li} L^{-1} LiOH+LiCl<br>Taijinar brine 1950 mg_{Li} L^{-1}	6 h at RT	26.90 mg_{Li} g^{-1}<br>24.46 mg_{Li} g^{-1}	0.8 M $Na_2S_2O_8$	3	[22]
**PVC + $H_{1.6}Mn_{1.6}O_4$ membrane**	Ion sieve	150 mg_{Li} L^{-1}	8 h at RT	26.64 mg_{Li} g^{-1}	0.5 M HCl	8	[23]
**TiO_2 nanoribbons**	Ion sieve	79 mg_{Li} L^{-1} LiCl<br>Mixed Li 69.4 mg_{Li} L^{-1}	130 rpm, 144 h at 303K	25.60 mg_{Li} g^{-1}<br>6.91 mg_{Li} g^{-1}	0.1 M HCl	N.A.	[24]
**Granulated LTO/PVB**	Ion sieve	200 mg_{Li} L^{-1} LiOH<br>200 mg_{Li} L^{-1} brine pH 9<br>200 mg_{Li} L^{-1} brine pH 7	180 rpm, 3h at 303K	25.00 mg_{Li} g^{-1}<br>12.00 mg_{Li} g^{-1}<br>7.50 mg_{Li} g^{-1}	0.2 M HCl	15	[25]
**GO+12C4E membrane**	Supramolecular system	10 mg_{Li} L^{-1} LiCl	3 h at RT	24.25 mg_{Li} g^{-1}	0.5 M HCl	10	[26]
**PVA/CAM-$H_4Mn_5O_{12}$**	Ion sieve	100 mg_{Li} L^{-1}	24 h at RT	23.26 mg_{Li} g^{-1}	0.5 M HCl	8	[27]
**IIP-GO/Fe_3O_4@C**	Supramolecular system	20 mg_{Li} L^{-1}	2h at RT	22.90 mg_{Li} g^{-1}	0.5 M HNO_3	6	[28]
**Layered H_2TiO_3 powder**	Ion sieve	Uyuni Salar 1630 mg_{Li} L^{-1}	24 h at RT	21.70 mg_{Li} g^{-1}	0.2 M HCl	N.A.	[29]

TYPE	METHODOLOGY	FEED SOLUTION	EXPERIMENTAL CONDITIONS	UPTAKE	REGENERATION SOLUTION	CYCLES	REF
**Cellulose/50 wt% $H_{1.33}Mn_{1.67}O_4$ film**	Ion sieve	25 mg_{Li} L^{-1} LiCl spiked seawater (0.21 mg_{Li} L^{-1})	48 h at RT	21.60 mg_{Li} g^{-1} 1.00 mg_{Li} g^{-1}	0.5 M HCl	8	[30]
**Ceramic foam LTO**	Ion sieve	2000 mg_{Li} L^{-1} LiOH	24 h at 298K	21.00 mg_{Li} g^{-1}	0.25 M HCl	N.A.	[31]
**Spherical ion-sieve foams (SIFs)**	Ion sieve	694 mg_{Li} L^{-1} LiOH Seawater	24 h at 298K	20.90 mg_{Li} g^{-1} 3.40 mg_{Li} g^{-1}	0.5 M HCl	5	[32]
**LTO powder**	Ion sieve	adjusted West Taijinar brines (615 mg_{Li} L^{-1}) West Taijinar brines (784 mg_{Li} L^{-1})	36 h at 303 K	19.22 mg_{Li} g^{-1} 10.30 mg_{Li} g^{-1}	0.09 M HCl	5	[33]
**PAN/90 wt% $H_{1.6}Mn_{1.6}O_4$ NF**	Ion sieve	spiked seawater 10 mg_{Li} L^{-1}	24 h at 298K	16.46 mg_{Li} g^{-1} (average of 14.73 mg_{Li} g^{-1})	0.5 M HCl	10	[34]
**Mixed LMTO**	Ion sieve	spiked seawater 60 mg_{Li} L^{-1}	4 days at RT	15.80 mg_{Li} g^{-1}	0.3 M HCl	5	[35]
**LMO powder**	Ion sieve	Wastewater 5 mg_{Li} L^{-1} Seawater	120 rpm, 72 h at 298K	15.20 mg_{Li} g^{-1} 11.90 mg_{Li} g^{-1}	0.5 M HCl	4	[36]
**PVA/200 wt% LTO foam**	Ion sieve	7 mg_{Li} L^{-1} LiOH+LiCl	24 h at 303K	14.00 mg_{Li} g^{-1}	0.2 M HCl	5	[37]
**LTO granulated with agar**	Ion sieve	geothermal water 25.8 mg_{Li} L^{-1}	10 h at 333K	12.29 mg_{Li} g^{-1}	0.25 M HCl	5	[38]
**PSf/38 wt% $H_{1.6}Mn_{1.6}O_4$ NF**	Ion sieve	spiked seawater 7 mg_{Li} L^{-1}	200-350 rpm 24 h at 298K	12.00 mg_{Li} g^{-1}	0.5 M HCl	5	[39]
**Granulated LMO/19.4 wt% chitosan**	Ion sieve	spiked seawater 30 mg_{Li} L^{-1}	7 days at 298K	11.40 mg_{Li} g^{-1}	0.2 M H_2SO_4	N.A.	[40]
**Granulated LMO/chitosan**	Ion sieve	Geothermal water 25.78 mg_{Li} L^{-1}	48 h at 333K	11.40 mg_{Li} g^{-1}	0.25 M HCl	6	[41]
**LTO granulated with PVC**	Ion sieve	geothermal water 25.8 mg_{Li} L^{-1}	12 h at 318K	11.35 mg_{Li} g^{-1}	0.25 M HCl	5	[42]
**Hollow spheres Si modified APTES-LMO**	Ion sieve	50 mg_{Li} L^{-1} LiCl	24 h at 298K	11.22 mg_{Li} g^{-1}	0.3 M HCl	N.A.	[43]
**PAN/60 wt%$H_{1.6}Mn_{1.6}O_4$ NF**	Ion sieve	35 mg_{Li} L^{-1} LiOH+LiCl Desal retentate (15.34 mg_{Li} L^{-1})	200 rpm, 24 h at 303K	10.30 mg_{Li} g^{-1} 7.43 mg_{Li} g^{-1}	0.5 M HCl	10	[44]
**Nanowires granulated $MnO_2 \cdot 0.5H_2O$**	Ion sieve	Spiked seawater 6.08 mg_{Li} L^{-1}	150 rpm, 48 h at 298K	10.05 mg_{Li} g^{-1}	0.5 M HCl	N.A.	[45]
**PVC + $H_{1.33}Mn_{1.67}O_4$ membrane**	Ion sieve	Seawater	20 days at 303K	9.50 mg_{Li} g^{-1}	0.75 M HCl	N.A.	[46]
**$H_{0.23}Li_{0.77}Ti_2O_4$ powder**	Ion sieve	Spiked seawater 100 mg_{Li} L^{-1}	100 rpm, 24 h at 298K	9.50 mg_{Li} g^{-1}	0.3 M HCl	3	[47]

TYPE	METHODOLOGY	FEED SOLUTION	EXPERIMENTAL CONDITIONS	UPTAKE	REGENERATION SOLUTION	CYCLES	REF
**Polymeric membrane reservoir (Kimtex + $H_{1.33}Mn_{1.67}O_4$)**	Ion sieve	100 mg_{Li} L^{-1}	24 h at RT	9.02 mg_{Li} g^{-1}	0.5 M HCl	N.A.	[48]
**LMO foam prepared with polyurethane template**	Ion sieve	694 mg_{Li} L^{-1} LiOH<br>694 mg_{Li} L^{-1} LiCl<br>brine 237 mg_{Li} L^{-1}	24 h at 298K	8.73 mg_{Li} g^{-1}<br>3.83 mg_{Li} g^{-1}<br>1.49 mg_{Li} g^{-1}	0.5 M HCl	N.A.	[49]
**PVA/$H_{1.6}Mn_{1.6}O_4$ foam**	Ion sieve	7 mg_{Li} L^{-1} LiOH+LiCl	175 rpm, 24 h at 298K	7.77 mg_{Li} g^{-1}	0.5 M HCl	5	[50]
**12C4E polymer brushes**	Supramolecular system	10 mg_{Li} L^{-1}	6 h at 298K	4.43 mg_{Li} g^{-1}	HNO_3 4%	5	[51]
**Fe_3O_4@SiO_2@IIPs with 12C4E**	Supramolecular system	104 mg_{Li} L^{-1}	12 h at 298K	4.06 mg_{Li} g^{-1}	0.5 M HCl	5	[52]
**HPS+12C4E (5.74 wt%)**	Supramolecular system	100 mg_{Li} L^{-1} LiCl<br>100 mg_{Li} L^{-1} LiCl + others	12 h at 298K	1.79 mg_{Li} g^{-1} 1.16 mg_{Li} g^{-1}	1 M HNO_3	5	[53]
**Fe_3O_4@SiO_2@IIPs with 12C4E**	Supramolecular system	1 mg_{Li} L^{-1}	6 h at 298K	0.125 mg_{Li} g^{-1}	1 M HNO_3	5	[54]

Table S2. Summary table of the electrochemical processes and cases studied.

TYPE	METHODOLOGY	FEED SOLUTION	ELECTROCHEM. CONDITIONS	UPTAKE, RECOVERY (R)	RECOVERY SOLUTION	CYCLES	PURITY (P) SELECTIVITY (S)	ENERGY CONSUMPTION	REF
**λ-MnO_2/Pt**	1st paper	LiCl solutions	CV @0.1 mV s^{-1}	11 mg_{Li} g^{-1}	LiCl solutions	N.A.	N.A.	N.A.	[55]
**LFP/Ag**	ion-pumping	Na:Li = 100:1	±0.5 mA cm^{-2} @2 h	Na:Li = 1:5	50 mM KCl	N.A.	99.9% (P)	144 Wh kg_{Li}^{-1}	[56]
**LFP/NiHCF**	ion-pumping	Atacama brine	1C:1C	$[Li]_{final}$= 92 mM	Atacama	N.A.	11.2% (P)	8.7 Wh mol_{Li}^{-1}	[57]
			1C:1C	$[Li]_{final}$ = 50 mM	Sea Water	N.A.	10.6% (P)	11.2 Wh mol_{Li}^{-1}	
			1C:1C	$[Li]_{final}$ 66 mM	90 mM KCl	N.A.	74% (P)	12.9 Wh mol_{Li}^{-1}	
**LFP/NiHCF**	ion-pumping	Atacama Brine	±0.5 mA cm^{-2} @1 h	$[Li]_{final}$= 80 mM	50 mM KCl	N.A.	N.A.	38 Wh mol_{Li}^{-1}	[58]
**$LiFePO_4/FePO_4$**	ion-pumping: Rocking chair	220 mg_{Li} L^{-1}	1.0 V @ 10 h	41.26 mg_{Li} g^{-1}	0.5 M NaCl	N.A.	N.A.	N.A.	[59]
		West China brine (220 mg_{Li} L^{-1}, Mg:Li=60)		28.65 mg_{Li} g^{-1} (final Mg:Li= 0.4)		N.A.	N.A.	N.A.	
**LMO/Ag microparticles**	ion-pumping	30 mM LiCl	0.5 mA cm^{-2} @30 min	Δ[Li]= 18 mM	30 mM LiCl	4	90% (P) 30%(S)	1 Wh mol_{Li}^{-1}	[60]
**LMO/Ag**	ion-pumping	Atacama Brine	1C:1C	$[Li]_{final}$ =71 mM	91 mM $CaCl_2$	7	96%(P) Li:Mg 7100 (S)	4.1 Wh mol_{Li}^{-1}	[61]
**LMO/NiHCF**	ion-pumping	Atacama Brine	1C:1C	$[Li]_{final}$ = 98 mM	156.3 mM KCl	7	61.3%(P) Li:Mg 1633 (S)	3.6 Wh mol_{Li}^{-1}	[61]
			5C:1C	$[Li]_{final}$ = 41 mM			25%(P) Li:Mg 103 (S)	3.6 Wh mol_{Li}^{-1}	
			1C:5C	$[Li]_{final}$ = 86 mM			54%(P) Li:Mg 286 (S)	5.4 Wh mol_{Li}^{-1}	
			1C:10C	$[Li]_{final}$ = 85 mM			53%(P) Li:Mg 212 (S)	11.5 Wh mol_{Li}^{-1}	
**LMO/Ag composite**	ion-pumping	50-200 mM LiCl + 100 mM KCl	±5-5000 mAg^{-1} @0.2-1.2 V	2.5-22.5 mg_{Li} g_{LMO}^{-1}	N.A.	N.A.	N.A.	N.A.	[62]
**LMO/PPy**	ion-pumping	0.1 M $LiNO_3$, Salar de Olaroz brine	CV @2 mV s^{-1}	Theoretical value: 39 mg_{Li} g^{-1}	N.A.	N.A.	N.A.	N.A.	[63]

TYPE	METHODOLOGY	FEED SOLUTION	ELECTROCHEM. CONDITIONS	UPTAKE, RECOVERY (R)	RECOVERY SOLUTION	CYCLES	PURITY (P) SELECTIVITY (S)	ENERGY CONSUMPTION	REF
**LMO/PPy**	ion-pumping	Salar de Olaroz brine	±2.5 mA cm^{-2} @0-1 V	2 mg_{Li} g^{-1} (R ca. 50%)	25 mM LiCl brine	200	N.A.	10 Wh mol_{Li}^{-1}	[64]
**LMO/PPy and LMO/LMO**	ion-pumping	Salar de Olaroz brine	25 µA cm^{-2} @0-0.7 V	N.A.	0.1 M LiCl	N.A.	N.A.	spontaneous process	[65]
**LMO/Ag**	ion-pumping	100 mM LiCl<br>30 mM LiCl	±50 mA g^{-1} @ 30 min	Δ[Li]= 25 mM<br>Δ[Li]= 10 mM	10 mM LiCl<br>N.A.	100<br>40	N.A.<br>N.A.	N.A.<br>4.14 Wh mol_{Li}^{-1}	[66]
**$Li_xMn_2O_4$/ $Li_{1-x}Mn_2O_4$**	ion-pumping: Rocking chair	0.05 M LiCl, KCl<br>Simulated brine and concentrated seawater	1.2 V @ 120 min<br>0.6 V @ 120 min	34 mg_{Li} g^{-1} (93%)<br>22 mg_{Li} g^{-1}	0.05 M NaCl<br>0.05 M KCl	5<br>5	N.A.<br>Li:Mg ~72 (S)	33 Wh mol_{Li}^{-1}<br>18 Wh mol_{Li}^{-1}	[67]
**$Li_xMn_2O_4$/ $Li_{1-x}Mn_2O_4$**	ion-pumping: Rocking chair Porous packed bed electrodes	Natural brine from Salar de Hombre Muerto	1.43 mA·cm^{-2}	Δ[Li]= 30 mM	0.1 M KCl	N.A.	N.A.	2.76 Wh mol_{Li}^{-1}	[68]
**$Li_xMn_2O_4$/ $Li_{1-x}Mn_2O_4$**	ion-pumping: Rocking chair Flow-by reactor	Natural brine from Salar de Hombre Muerto	20 mA @ 3.57h	9.14 mg_{Li} g^{-1}	0.1 M KCl	N.A.	N.A.	2.16 Wh mol_{Li}^{-1}	[69]
**$Li_xMn_2O_4$/ $Li_{1-x}Mn_2O_4$**	ion-pumping: Rocking chair	0.05 M LiCl, 0.1 M $MgCl_2$	Short-circuit + 0.6 V @ 3h	33.5 mg_{Li} g^{-1}	0.05 M KCl	6	Mg:Li ~0.018 (S)	7.63 Wh mol^{-1}	[70]
**λ-MnO_2/$LiMn_2O_4$ on Graphite felt**	ion-pumping: Rocking chair	22 mM LiCl, 41 mM $MgCl_2$, 43 mM NaCl 26 mM KCl	0.2 V – 0.9 V @ 10 min	24.02 mg_{Li} h^{-1}<br>75.06 mg_{Li} g^{-1} h^{-1}	14 mM LiCl	5	αLi-Mg = 45.56	23.4 Wh mol_{Li}^{-1}	[71]
**$LiFePO_4$/C**	ion-pumping: Rocking chair	5 mM LiCl, 50 mM NaCl	-0.5 V to 0.5 V @ 0.1 A g^{-1}	21 mg_{Li} g^{-1}	5 mM LiCl, 50 mM NaCl	10	N.A.	3.03 Wh mol_{Li}^{-1}	[72]
**LMO/Ag**	ion-pumping	Synthetic brine 63 mM Li	-10 µA cm^{-2} 0.2 V @17 h<br>0.2 mA cm^{-2} 0.9 V @21 min	7 mg_{Li} g^{-1}<br>10.1 mg g^{-1} day^{-1}	30 mM of KCl	N.A.	99%(P)	3.07 Wh g_{Li}^{-1}	[73]
**LMO/Ag**	ion-pumping: pilot-scale demonstration	Desalination retentate 0.035 mM Li	−10 µA cm^{-2} @0.2 V<br>+50 µA cm^{-2} @1.2 V	62 mM Li	N.A.	5	88%(P)<br>Li:Mg > 20 (S)	N.A.	[74]

**Type**	**Methodology**	**Feed solution**	**Electrochem. Conditions**	**Uptake, Recovery (R)**	**Recovery solution**	**Cycles**	**Purity (P) Selectivity (S)**	**Energy Consumption**	**Ref**
**LMO/NiHCF**	ion-pumping	1-10 mM LiCl / Atacama brine (40 mM)	±1 mA @ ±0.6 V	Δ[Li]= 10 mM	120 mM KCl	10	N.A.	N.A.	[75]
**LMO/NiHCF**	ion-pumping: flow through	1 mM LiCl + 100 mM NaCl	-0,5 - 1 mA cm^{-2} @ 0 - 0.8 V	78 mg_{Li} g_{LMO}^{-1} $\eta_{extraction}$=37%	120 mM KCl	9	94%(P)	6.1 Wh mol_{Li}^{-1} $\eta_{coulombic}$=74%	[76]
**$Li_{1.16}Mn_{0.6}Ni_{0.12}Co_{0.12}O_2$ / Bi**	ion-pumping	23.48 mM Li^+	± 0.50 - 1.25 mA @ 20min	1.88 mmol g^{-1}	10 mM LiCl	15	92%(P)	1.8 - 4.46 Wh mol_{Li}^{-1}	[77]
**λ-MnO_2 / BiOCl@PPy**	ion-pumping	100 mM LiCl + 2.5 M Na_2SO_4	Short circuit @ 3h Regeneration 0.4 – 1.2V @ 10min	10.88 mg g^{-1}	10 mM HNO_3	100	N.A.	1 Wh mol_{LiCl}^{-1}	[78]
**Redox couple of ferri-/ferrocyanide with adsorbent (λ-MnO_2)**	Redox-mediated **$Fe^{(III)}(CN)_6^{3-}$ $Fe^{(II)}(CN)_6^{4}$**	5 mM LiCl + 5mM NaCl 100 mM NaCl + 100 mM $Na_4Fe(CN)_6$	0.6 V – 1.2 V @ 4h	$[Li]_{final}$ = 17 – 37 mM	10 mM KCl		αLi-Na 57 (with presence of LMO)	57 - 140 kJ mol_{Li}^{-1}	[79]
**LMO/AC**	MHCDI	60 $mg_{Li}L^{-1}$ LiOH	3.5 V @70 h	8.7 mg_{Li} g_t^{-1}	DIW	N.A	N.A.	N.A	[80]
**LMO/AC**	MHCDI	50 mg_{Li} L^{-1} LiOH 500 mg_{Li} L^{-1} LiOH	1 V @40 min	2 mg_{Li} g^{-1} 4 mg_{Li} g^{-1}	DIW	5	N.A.	N.A	[81]
		50 mg_{Li} L^{-1} LiOH	1 V @1000 min	24 mg_{Li} g^{-1}		1	N.A.	N.A	
**LMO/AC**	MHCDI	30 mM LiCl	±0.5 mA cm^{-2} 0 V and 1.1 V @30 min	Δ[Li] ~6 mM	N.A.	50	N.A.	4.2 Wh mol_{Li}^{-1}	[82]
		simulated brine Salar de Atacama		Δ[Li] ~19.1 mM	N.A.	3	N.A.	N.A.	
**LMO/AC**	HCDI MCDI	10 mM LiCl	1V @24 h 1V @100 min	17mg_{LiCl} g^{-1} 30mg_{LiCl} g_{AC}^{-1}	N.A.	N.A.	N.A.	N.A.	[83]
**HMO/AC**	MHCDI	simulated brine Salar de Atacama	-1 V 3.5 V @40 min	0.35 $mmol_{Li}$ $g_{adsorbent}^{-1}$	DIW	5	N.A.	W_{ads}=4.4 Wh g_{Li}^{-1} W_{des}=23.3 Wh g_{Li}^{-1}	[84]

TYPE	METHODOLOGY	FEED SOLUTION	ELECTROCHEM. CONDITIONS	UPTAKE, RECOVERY (R)	RECOVERY SOLUTION	CYCLES	PURITY (P) SELECTIVITY (S)	ENERGY CONSUMPTION	REF
**Li-TiO_2-MnO_2/AC**	HCDI	10 mM LiCl, NaCl and KCl	0.25 - 1.5 V @1 min	36 mg_{LiCl} g^{-1} min^{-1}	N.A.	50	N.A.	0.15 kWh m^{-3} adsorbed salt	[85]
**LMTO /AC**	HCDI	geothermal water 15.7 mg_{Li} L^{-1}	±2 V, 0 V	800 mg_{LiCl} g^{-1}	DIW	N.A.	N.A.	0.183 Wh g^{-1} adsorbed salt	[86]
**AC/AC**	MCDI	500 mg_{TDS} L^{-1} , LiCl:$MgCl_2$ 1:1	1 V @ 10 min	38.4% Li<br>19.2% Mg	N.A.	N.A.	Li:Mg 2.95 (S)	0.0018 kWh mol^{-1}	[87]
**Nanoporous AC**	Flow-electrode-CDI	100 mg L^{-1} LiCl	1.2 V @ 2h	376 mg h^{-1}<br>215.06 µmol/m^{-2} s^{-1}<br>$\eta_{salt\ removal}$ =34%	N.A.	24h stability	N.A.	N.A.	[88]
**CoP/Co_3O_4-graphene aerogel**	CDI	50 mg L^{-1} Li^+	0.2 – 0.6 V @ 2h	10 - 37 mg_{Li^+} g^{-1}<br>70% Li	N.A.	10	N.A.	N.A.	[89]
**LiO-FeO-Mn_2O_3 /AC**	HCDI	10 mM LiCl	1 V @ 5min<br>0.07 – 0.19 mA cm^{-2} @ 5-20min	Average 32 mg_{LiCl} g^{-1}<br>(10 mM LiCl<br>0.07 mA cm^{-2})<br>Ca. 45% Li<br>(Geothermal water)	DIW	30	Na:K:Li from 227:1.1:1 to 2.9:0:1 (Geothermal water)	ca. 0.1 – 0.25 Wh g_{salt}^{-1}	[90]
**Li_3VO_4 on rGO / AC**	HCDI	610 mg L^{-1} LiCl	0.8 – 1.2 V @ 1h	25 - 39 mg_{LiCl} g^{-1}	LiCl	20	N.A.	N.A.	[91]
**MnOx / AC**	MHCDI	675 mg_{Li+} L^{-1}<br>Na/Li ratio = 48.6	1.2 V @ 550s	51.8 mg g^{-1}	DW	8	Final Na/Li ratio = 4.6	N.A.	[92]
**SELEMION™ CMV/TMPA–TFSI ED**	ED with IL	Concentrated seawater (anode side), 0.1 M HCl (cathode side)	2–3 V up to 15 h	Δ[Li] in anode side= 63% @ 2V 38% @ 3V	N.A.	N.A.	N.A.	N.A.	[93]
**GoreTex, PP13-TFSI, nafion membrane ED**	ED with membrane IL	seawater	2 V @2 h	Δ[Li]= 22.2%	HCl	N.A.	N.A.	N.A.	[94]

TYPE	METHODOLOGY	FEED SOLUTION	ELECTROCHEM. CONDITIONS	UPTAKE, RECOVERY (R)	RECOVERY SOLUTION	CYCLES	PURITY (P) SELECTIVITY (S)	ENERGY CONSUMPTION	REF
**Li conductive glass ED**	ED	seawater	2 V @24 h	Δ(R)= 7% after 72 h with no applied electric voltage	0.1 M HCl	N.A.	N.A.	N.A.	[95]
**ion exchange membrane ED**	ED	Na-contaminated LiBr (3.97 M Li^+)	7 V @ 6 h	14% (R)	2.46 mM NaOH, 0.1416 mM LiOH	N.A.	Li:Na=3.5 (S)	N.A.	[96]
**BPED**	bipolar membrane ED	340 mg_{Li} L^{-1} 1000 mg_B L^{-1}	25-35 V @ 2 h	R > 90%	0.1 M HCl, 0.1 M NaOH	N.A.	N.A.	N.A.	[97]
**BPED**	bipolar membrane ED	$Li_2B_4O_7.5H_2O$ (ca. 850 mg_B L^{-1} and 250 $mg_{Li}L^{-1}$)	15 V @ 2 h	Li: 99.6% and 88.3%(R) B: 72.3% and 70.8%(R)	3 mM HCl, 3 mM NaOH	N.A.	N.A.	N.A.	[98]
**S-ED**	selective membranes ED	0.15 g_{Li} L^{-1} and 10–60 g_{Mg} L^{-1} (Mg:Li = 66.7– 400)	5.9 Am^{-2} @ 3h	Mg:Li (<10), >90%(R) when Mg:Li= 150	NaCl (3 wt%) Na_2SO_3 (3 wt%)	N.A.	Li:Mg=20.2–33(S)	0.02 kWh g_{Li}^{-1}	[99]
**S-ED**	monovalent selective membranes ED	East-Taijiner brine (5.67 $g_{Li}L^{-1}$)	20 V @ 3.5 h	90.5%(R). Mg:Li from 20.7 to 2.07	NaCl (3 wt%) Na_2SO_3 (3 wt%)	N.A.	Li:Mg=9,89 (S)	0.0045 $kWhg_{Li}^{-1}$	[100]
**S-ED**	selective-ED	50 mM Li, Mg:Li=1-30	5 V @ 2 h	60-80%(R)	N.A.	N.A.	Mg:Li=5-60 (S)	0.026-0.13 kWh mol_{Li}^{-1}	[101]
**S-ED**	monovalent selective membranes ED	artificial brine Mg:Li = 1:20, 50 mM Li	3-8 V @ 2 h	15-70%(R)	N.A.	N.A.	Mg:Li=1-8 (S)	0.10-0.32 kWh mol_{Li}^{-1}	[102]

TYPE	METHODOLOGY	FEED SOLUTION	ELECTROCHEM. CONDITIONS	UPTAKE, RECOVERY (R)	RECOVERY SOLUTION	CYCLES	PURITY (P) SELECTIVITY (S)	ENERGY CONSUMPTION	REF
S-ED	monovalent selective membranes ED	seawater 0.14 g_{Li} L^{-1}, (Mg:Li 16.07)	3-9 V @ 2 h	10-80%(R)	NaCl	N.A.	N.A.	0.5-1.75 kWh mol_{Li}^{-1}	[103]
		East Taijinar brine	6-10 V @ 3 h	30-75%(R)	NaCl	N.A.	N.A.	0.5-0.66 kWh mol_{Li}^{-1}	
		West Taijinar brine	10 V @ 3 h	65%(R)	NaCl	N.A.	N.A.	4.25 kWh mol_{Li}^{-1}	
		Brine (Mg:Li = 65-90)	5 V @ 2 h	72%(R)	N.A.	N.A.	Mg:Li= 12.5 (S)	0.18 kWh mol_{Li}^{-1}	
CEM + (TBP) and RTIL membrane	IL-membrane ED	Brine (Mg:Li = 50)	4.4 Am^{-2} @12 h	Mg:Li= 0.5	HCl	N.A.	N.A.	16 Wh·g_{Li}^{-1}	[104]
		West Taijinair (Mg:Li= 53)	6.2 Am^{-2} @12 h	Mg:Li= 0.26	HCl	N.A.	N.A.	N.A.	

REFERENCES

[1] X. Zheng, A. Li, J. Hua, Y. Zhang, Z. Li, *Nanomaterials* **2021**, *11*, 2668.

[2] S. Zhou, X. Guo, X. Yan, Y. Chen, W. Lang, *Particuology* **2022**, *69*, 100.

[3] C. P. Lawagon, G. M. Nisola, J. Mun, A. Tron, R. E. C. Torrejos, J. G. Seo, H. Kim, W.-J. Chung, *J. Ind. Eng. Chem.* **2016**, *35*, 347.

[4] T. Ryu, Y. Haldorai, A. Rengaraj, J. Shin, H.-J. Hong, G.-W. Lee, Y.-K. Han, Y. S. Huh, K.-S. Chung, *Ind. Eng. Chem. Res.* **2016**, *55*, 7218.

[5] C. Yu, J. Lu, J. Dai, Z. Dong, X. Lin, W. Xing, Y. Wu, Z. Ma, *J. Colloid Interface Sci.* **2020**, *572*, 340.

[6] Q. H. Zhang, S. P. Li, S. Y. Sun, X. S. Yin, J. G. Yu, *Adv. Powder Technol.* **2009**, *20*, 432.

[7] D. Gu, W. Sun, G. Han, Q. Cui, H. Wang, *Chem. Eng. J.* **2018**, *350*, 474.

[8] R. Chitrakar, H. Kanoh, Y. Miyai, K. Ooi, *Ind. Eng. Chem. Res.* **2001**, *40*, 2054.

[9] M. Moazeni, H. Hajipour, M. Askari, M. Nusheh, *Mater. Res. Bull.* **2015**, *61*, 70.

[10] L. Zhang, D. Zhou, G. He, F. Wang, J. Zhou, *Mater. Lett.* **2014**, *135*, 206.

[11] N. Li, K. Gan, D. Lu, J. Zhang, L. Wang, *Res. Chem. Intermed.* **2018**, *44*, 1105.

[12] S. M. Hossain, I. Ibrahim, Y. Choo, A. Razmjou, G. Naidu, L. Tijing, J.-H. Kim, H. K. Shon, *Desalination* **2022**, *525*, 115491.

[13] L. Wang, C. G. Meng, W. Ma, *Colloids Surfaces A Physicochem. Eng. Asp.* **2009**, *334*, 34.

[14] S. Wang, P. Li, X. Zhang, S. Zheng, Y. Zhang, *Hydrometallurgy* **2017**, *174*, 21.

[15] X. Dai, H. Zhan, Z. Qian, J. Li, Z. Liu, Z. Wu, *RSC Adv.* **2021**, *11*, 34988.

[16] K. Zhao, B. Tong, X. Yu, Y. Guo, Y. Xie, T. Deng, *Chem. Eng. J.* **2022**, *430*, 131423.

[17] X. Li, L. Chen, Y. Chao, W. Chen, J. Luo, J. Xiong, F. Zhu, X. Chu, H. Li, W. Zhu, *Chem. Eng. Technol.* **2020**, *43*, 1784.

[18] N. Li, D. Lu, J. Zhang, L. Wang, *J. Colloid Interface Sci.* **2018**, *520*, 33.

[19] J. Lu, Y. Qin, Q. Zhang, Y. Wu, J. Cui, C. Li, L. Wang, Y. Yan, *Appl. Surf. Sci.* **2018**, *427*, 931.

[20] J. Cui, Y. Zhang, Y. Wang, J. Ding, P. Yu, Y. Yan, C. Li, Z. Zhou, *New J. Chem.* **2018**, *42*, 118.

[21] D. Sun, Y. Zhu, M. Meng, Y. Qiao, Y. Yan, C. Li, *Sep. Purif. Technol.* **2017**, *175*, 19.

[22] Z.-Y. Ji, F.-J. Yang, Y.-Y. Zhao, J. Liu, N. Wang, J.-S. Yuan, *Chem. Eng. J.* **2017**, *328*, 768.

[23] G. Zhu, P. Wang, P. Qi, C. Gao, *Chem. Eng. J.* **2014**, *235*, 340.

[24] Q. H. Zhang, S. P. Li, S. Y. Sun, X. S. Yin, J. G. Yu, *Chem. Eng. Sci.* **2010**, *65*, 165.

[25] Y. Zhang, J. Liu, Y. Yang, S. Lin, P. Li, *Sep. Purif. Technol.* **2021**, *267*, 118613.

[26] D. Sun, M. Meng, Y. Lu, B. Hu, Y. Yan, C. Li, *New J. Chem.* **2018**, *42*, 4432.

[27] Z. Qiu, M. Wang, Y. Chen, T. Zhang, D. Yang, F. Qiu, *Desalination* **2021**, *506*, 115003.

[28] H. Zhao, Q. Liang, Y. Yang, W. Liu, X. Liu, *Sep. Purif. Technol.* **2021**, *265*, 118513.

[29] R. Chitrakar, Y. Makita, K. Ooi, A. Sonoda, *Dalt. Trans.* **2014**, *43*, 8933.

[30] L. Tang, S. Huang, Y. Wang, D. Liang, Y. Li, J. Li, Y. Wang, Y. Xie, W. Wang, *ACS Appl. Mater. Interfaces* **2020**, *12*, 9775.

[31] L. Zhang, D. Zhou, G. He, Q. Yao, F. Wang, J. Zhou, *Mater. Lett.* **2015**, *145*, 351.

[32] Y. Han, H. Kim, J. Park, *Chem. Eng. J.* **2012**, *210*, 482.

[33] X. Zhu, H. Yue, W. Sun, L. Zhang, Q. Cui, H. Wang, *Sep. Purif. Technol.* **2021**, *274*, 119099.

[34] W.-J. Chung, R. E. C. Torrejos, M. J. Park, E. L. Vivas, L. A. Limjuco, C. P. Lawagon, K. J. Parohinog, S.-P. Lee, H. K. Shon, H. Kim, G. M. Nisola, *Chem. Eng. J.* **2017**, *309*, 49.

[35] T. Ryu, J. Shin, S. M. Ghoreishian, K.-S. Chung, Y. S. Huh, *Hydrometallurgy* **2019**, *184*, 22.

[36] H. J. Park, N. Singhal, E. H. Jho, *Water Res.* **2015**, *87*, 320.

[37] L. A. Limjuco, G. M. Nisola, C. P. Lawagon, S. P. Lee, J. G. Seo, H. Kim, W.-J. Chung, *Colloids Surfaces A Physicochem. Eng. Asp.* **2016**, *504*, 267.

[38] S. Chen, Z. Chen, Z. Wei, J. Hu, Y. Guo, T. Deng, *Chem. Eng. J.* **2021**, *410*, 128320.

[39] M. J. Park, G. M. Nisola, E. L. Vivas, L. A. Limjuco, C. P. Lawagon, J. G. Seo, H. Kim, H. K. Shon, W.-J. Chung, *J. Memb. Sci.* **2016**, *510*, 141.

[40] H. J. Hong, I. S. Park, T. Ryu, J. Ryu, B. G. Kim, K.-S. Chung, *Chem. Eng. J.* **2013**, *234*, 16.

[41] W. Ding, J. Zhang, Y. Liu, Y. Guo, T. Deng, X. Yu, *Chem. Eng. J.* **2021**, *426*, 131689.

[42] H. Lin, X. Yu, M. Li, J. Duo, Y. Guo, T. Deng, *ACS Appl. Mater. Interfaces* **2019**, *11*, 26364.

[43] Y. Wang, J. Xu, X. Xu, D. Yang, X. Zheng, J. Pan, T. Zhang, F. Qiu, C. Li, *Appl. Organomet. Chem.* **2018**, *32*, 1.

[44] M. J. Park, G. M. Nisola, A. B. Beltran, R. E. C. Torrejos, J. G. Seo, S.-P. Lee, H. Kim, W.-J. Chung, *Chem. Eng. J.* **2014**, *254*, 73.

[45] L. Liu, H. Zhang, Y. Zhang, D. Cao, X. Zhao, *Colloids Surfaces A Physicochem. Eng. Asp.* **2015**, *468*, 280.

[46] A. Umeno, Y. Miyai, N. Takagi, R. Chitrakar, K. Sakane, K. Ooi, *Ind. Eng. Chem. Res.* **2002**, *41*, 4281.

[47] C.-W. Chen, P.-A. Chen, C.-J. Wei, H.-L. Huang, C.-J. Jou, Y.-L. Wei, H. P. Wang, *Mar. Pollut. Bull.* **2017**, *124*, 1106.

[48] K. Chung, J. Lee, W. Kim, S. Kim, K. Cho, *J. Memb. Sci.* **2008**, *325*, 503.

[49] L.-W. Ma, B.-Z. Chen, Y. Chen, X.-C. Shi, *Microporous Mesoporous Mater.* **2011**, *142*, 147.

[50] G. M. Nisola, L. A. Limjuco, E. L. Vivas, C. P. Lawagon, M. J. Park, H. K. Shon, N. Mittal, I. W. Nah, H. Kim, W.-J. Chung, *Chem. Eng. J.* **2015**, *280*, 536.

[51] X. Bai, J. Dai, Y. Ma, W. Bian, J. Pan, *Chem. Eng. J.* **2020**, *380*, 122386.

[52] X. Luo, B. Guo, J. Luo, F. Deng, S. Zhang, S. Luo, J. Crittenden, *ACS Sustain. Chem. Eng.* **2015**, *3*, 460.

[53] X. Xu, F. Qiu, D. Yang, X. Zheng, Y. Wang, J. Pan, T. Zhang, J. Xu, C. Li, *Appl. Organomet. Chem.* **2018**, *32*, 1.

[54] J. Xu, Z. Pu, X. Xu, Y. Wang, D. Yang, T. Zhang, F. Qiu, *Appl. Organomet. Chem.* **2019**, *33*, e4778.

[55] H. Kanoh, K. Ooi, Y. Miyai, S. Katoh, *Langmuir* **1991**, *7*, 1841.

[56] M. Pasta, A. Battistel, F. La Mantia, *Energy Environ. Sci.* **2012**, *5*, 9487.

[57] R. Trócoli, A. Battistel, F. La Mantia, *ChemSusChem* **2015**, *8*, 2514.

[58] R. Trócoli, G. K. Bidhendi, F. La Mantia, *J. Phys. Condens. Matter* **2016**, *28*, 114005.

[59] Z. Zhao, X. Si, X. Liu, L. He, X. Liang, *Hydrometallurgy* **2013**, *133*, 75.

[60] J. Lee, S.-H. Yu, C. Kim, Y.-E. Sung, J. Yoon, *Phys. Chem. Chem. Phys.* **2013**, *15*, 7690.

[61] R. Trócoli, C. Erinmwingbovo, F. La Mantia, *ChemElectroChem* **2017**, *4*, 143.

[62] S. Kim, J. S. Kang, H. Joo, Y.-E. Sung, J. Yoon, *Environ. Sci. Technol.* **2020**, *54,* 9044.

[63] F. Marchini, D. Rubi, M. Del Pozo, F. J. Williams, E. J. Calvo, *J. Phys. Chem. C* **2016**, *120*, 15875.

[64] L. L. Missoni, F. Marchini, M. del Pozo, E. J. Calvo, *J. Electrochem. Soc.* **2016**, *163*, A1898.

[65] F. Marchini, F. J. Williams, E. J. Calvo, *J. Electrochem. Soc.* **2018**, *165*, A3292.

[66] X. Xu, Y. Zhou, Z. Feng, N. U. Kahn, Z. U. Haq Khan, Y. Tang, Y. Sun, P. Wan, Y. Chen, M. Fan, *Chempluschem* **2018**, *83,* 521.

[67] M.-Y. Zhao, Z.-Y. Ji, Y.-G. Zhang, Z.-Y. Guo, Y.-Y. Zhao, J. Liu, J.-S. Yuan, *Electrochim. Acta* **2017**, *252*, 350.

[68] V. C. E. Romero, D. S. Putrino, M. Tagliazucchi, V. Flexer, E. J. Calvo, *J. Electrochem. Soc.* **2020**, *167*, 120522.

[69] V. C. E. Romero, K. Llano, E. J. Calvo, *Electrochem. commun.* **2021**, *125*, 106980.

[70] Z.-Y. Guo, Z.-Y. Ji, J. Wang, H.-Y. Chen, J. Liu, Y.-Y. Zhao, F. Li, J.-S. Yuan, *Sep. Purif. Technol.* **2021**, *259*, 118154.

[71] Y. Mu, C. Zhang, W. Zhang, Y. Wang, *Desalination* **2021**, *511*, 115112.

[72] L. Wang, K. Frisella, P. Srimuk, O. Janka, G. Kickelbick, V. Presser, *Sustain. Energy Fuels* **2021**, *5*, 3124.

[73] S. Kim, H. Joo, T. Moon, S.-H. Kim, J. Yoon, *Environ. Sci. Process. Impacts* **2019**, *21*, 667.

[74] H. Joo, S. Kim, S. Kim, M. Choi, S.-H. Kim, J. Yoon, *Environ. Sci. Water Res. Technol.* **2020**, *6,* 290.

[75] M. S. Palagonia, D. Brogioli, F. La Mantia, *J. Electrochem. Soc.* **2017**, *164*, E586.

[76] M. S. Palagonia, D. Brogioli, F. La Mantia, *Desalination* **2020**, *475*, 114192.

[77] X. Zhao, H. Yang, Y. Wang, L. Yang, L. Zhu, *Sep. Purif. Technol.* **2021**, *274*, 119078.

[78] J. Niu, W. Yan, X. Song, W. Ji, Z. Wang, X. Hao, G. Guan, *Sep. Purif. Technol.* **2021**, *274*, 118995.

[79] N. Kim, X. Su, C. Kim, *Chem. Eng. J.* **2021**, *420*, 127715.

[80] T. Ryu, J. C. Ryu, J. Shin, D. H. Lee, Y. H. Kim, K.-S. Chung, *Ind. Eng. Chem. Res.* **2013**, *52*, 13738.

[81] T. Ryu, D.-H. Lee, J. C. Ryu, J. Shin, K.-S. Chung, Y. H. Kim, *Hydrometallurgy* **2015**, *151*, 78.

[82] S. Kim, J. Lee, J. S. Kang, K. Jo, S. Kim, Y. E. Sung, J. Yoon, *Chemosphere* **2015**, *125*, 50.

[83] M. Bryjak, A. Siekierka, J. Kujawski, K. ìatarzyn. Smolińska-Kempisty, W. Kujawski, *J. Membr. Sep. Technol.* **2015**, *4*, 110.

[84] D. H. Lee, T. Ryu, J. Shin, J. C. Ryu, K.-S. Chung, Y. H. Kim, *Hydrometallurgy* **2017**, *173*, 283.

[85] A. Siekierka, J. Kujawa, W. Kujawski, M. Bryjak, *Sep. Purif. Technol.* **2018**, *194*, 231.

[86] A. Siekierka, B. Tomaszewska, M. Bryjak, *Desalination* **2018**, *436*, 8.

[87] W. Shi, X. Liu, C. Ye, X. Cao, C. Gao, J. Shen, *Sep. Purif. Technol.* **2019**, *210*, 885.

[88] Y. Ha, H. Bin Jung, H. Lim, P. S. Jo, H. Yoon, C.-Y. Yoo, T. K. Pham, W. Ahn, Y. Cho, *Energies* **2019**, *12*, 2913.

[89] W. Jin, M. Hu, Z. Sun, C.-H. Huang, H. Zhao, *Chem. Eng. J.* **2021**, *420*, 127661.

[90] A. Siekierka, *Sep. Purif. Technol.* **2020**, *236*, 116234.

[91] X. Shang, Z. Liu, W. Ji, H. Li, *Sep. Purif. Technol.* **2021**, *262*, 118294.

[92] Y. Sun, Y. Wang, Y. Liu, X. Xiang, *ACS Sustain. Chem. Eng.* **2021**, *9*, 11022.

[93] T. Hoshino, *Fusion Eng. Des.* **2013**, *88*, 2956.

[94] T. Hoshino, *Desalination* **2013**, *317*, 11.

[95] T. Hoshino, *Desalination* **2015**, *359*, 59.

[96] N. Parsa, A. Moheb, A. Mehrabani-Zeinabad, M. A. Masigol, *Chem. Eng. Res. Des.* **2015**, *98*, 81.

[97] S. Bunani, K. Yoshizuka, S. Nishihama, M. Arda, N. Kabay, *Desalination* **2017**, *424*, 37.

[98] S. Bunani, N. Kabay, S. Bunani, M. Arda, K. Yoshizuka, S. Nishihama, S. Bunani, *Desalination* **2017**, *416*, 10.

[99] X. Y. Nie, S. Y. Sun, Z. Sun, X. Song, J. G. Yu, *Desalination* **2017**, *403*, 128.

[100] X. Y. Nie, S. Y. Sun, X. Song, J. G. Yu, *J. Memb. Sci.* **2017**, *530*, 185.

[101] Q.-B. Chen, Z.-Y. Ji, J. Liu, Y.-Y. Zhao, S.-Z. Wang, J.-S. Yuan, *J. Memb. Sci.* **2018**, *548*, 408.

[102] P.-Y. Ji, Z.-Y. Ji, Q.-B. Chen, J. Liu, Y.-Y. Zhao, S.-Z. Wang, F. Li, J.-S. Yuan, *Sep. Purif. Technol.* **2018**, *207*, 1.

[103] Z.-Y. Guo, Z.-Y. Ji, Q.-B. Chen, J. Liu, Y.-Y. Zhao, F. Li, Z.-Y. Liu, J.-S. Yuan, *J. Clean. Prod.* **2018**, *193*, 338.

[104] G. Liu, Z. Zhao, L. He, *Desalination* **2020**, *474*, 114185.
